# [^18^F]Fluoroethyltriazolyl Monocyclam Derivatives as Imaging Probes for the Chemokine Receptor CXCR4

**DOI:** 10.3390/molecules24081612

**Published:** 2019-04-24

**Authors:** Alejandro Amor-Coarasa, James M. Kelly, Pradeep K. Singh, Shashikanth Ponnala, Anastasia Nikolopoulou, Clarence Williams, Yogindra Vedvyas, Moonsoo M. Jin, J. David Warren, John W. Babich

**Affiliations:** 1 Division of Radiopharmaceutical Sciences, Department of Radiology, Weill Cornell Medicine, New York, NY 10065, USA; alejandro.amor@einstein.yu.edu (A.A.-C.); jak2046@med.cornell.edu (J.M.K.); shp2018@med.cornell.edu (S.P.); ann2010@med.cornell.edu (A.N.); clw2012@med.cornell.edu (C.W.J.); 2 Molecular Imaging Innovations Institute (MI3), Department of Radiology, Weill Cornell Medicine, New York, NY 10065, USA; yov2002@med.cornell.edu (Y.V.); moj2005@med.cornell.edu (M.M.J.); 3 Milstein Chemistry Core Facility, Weill Cornell Medicine, New York, NY 10065, USA; prs2016@med.cornell.edu (P.K.S.); jdw2003@med.cornell.edu (J.D.W.); 4 Department of Biochemistry, Weill Cornell Medicine, New York, NY 10065, USA; 5 Citigroup Biomedical Imaging Center, Weill Cornell Medicine, New York, NY 10065, USA; 6 Sandra and Edward Meyer Cancer Center, Weill Cornell Medicine, New York, NY 10065, USA

**Keywords:** chemokine receptor, CXCR4, positron emission tomography, molecular modeling, drug discovery

## Abstract

Determining chemokine receptor CXCR4 expression is significant in multiple diseases due to its role in promoting inflammation, cell migration and tumorigenesis. [^68^Ga]Pentixafor is a promising ligand for imaging CXCR4 expression in multiple tumor types, but its utility is limited by the physical properties of ^68^Ga. We screened a library of >200 fluorine-containing structural derivatives of AMD-3465 to identify promising candidates for in vivo imaging of CXCR4 expression by positron emission tomography (PET). Compounds containing fluoroethyltriazoles consistently achieved higher docking scores. Six of these higher scoring compounds were radiolabeled by click chemistry and evaluated in PC3-CXCR4 cells and BALB/c mice bearing bilateral PC3-WT and PC3-CXCR4 xenograft tumors. The apparent CXCR4 affinity of the ligands was relatively low, but tumor uptake was CXCR4-specific. The tumor uptake of [^18^F]RPS-534 (7.2 ± 0.3 %ID/g) and [^18^F]RPS-547 (3.1 ± 0.5 %ID/g) at 1 h p.i. was highest, leading to high tumor-to-blood, tumor-to-muscle, and tumor-to-lung ratios. Total cell-associated activity better predicted in vivo tumor uptake than did the docking score or apparent CXCR4 affinity. By this metric, and on the basis of their high yielding radiosynthesis, high tumor uptake, and good contrast to background, [^18^F]RPS-547, and especially [^18^F]RPS-534, are promising ^18^F-labeled candidates for imaging CXCR4 expression.

## 1. Introduction

The transmembrane G-protein coupled receptor C-X-C chemokine receptor type 4 (CXCR4), which specifically binds to the chemokine CXCL12, is expressed on many cell types, including various types of cancer cells. Activation of CXCR4 by its natural ligand stromal cell-derived factor 1 (SDF-1) is known to be involved in a number of vital physiological processes such as inflammation [1], cell migration [2], and tumorigenesis [3]. CXCR4 plays a key role in organ-specific metastasis, directing the migration of malignant cells expressing this receptor toward microenvironments where the cognate ligand is secreted [4,5,6,7,8,9,10].

To date, there is no clinically approved imaging agent to assess CXCR4 expression in patients. The radiotracer, [^68^Ga]Pentixafor, a cyclic pentapeptide incorporating the chelate, DOTA, is under clinical investigation for imaging CXCR4 expression in Europe [11,12,13,14,15,16,17,18]. Although [^68^Ga]Pentixafor accumulates in CXCR4-positive tumors with acceptable tumor avidity and contrast, the short half-life of ^68^Ga (t_1/2_ = 68 min) limits its utility. In addition, as ^68^Ga is obtained from the ^68^Ge/^68^Ga generator the maximum produced activity of ^68^Ga is typically low (1.85 GBq) [19] precluding large-scale production for regional distribution.

The DOTA moiety in Pentixafor was added to conserve the presence of the high affinity cyclam ring [20] found in the small molecule CXCR4 antagonists AMD-3100 and AMD-3465. However, the CXCR4 affinity of the ^68^Ga complex was much greater than the complexes of other PET or SPECT radiometals such as ^111^In, precluding the substitution of longer-lived isotopes for ^68^Ga. Bicyclam-containing [^64^Cu]AMD-3100 was taken up in tumors in a CXCR4-dependent manner, but high signal in liver and bone resulted in modest tumor-to-background ratios [21]. The higher affinity radioligand, [^64^Cu]AMD-3465, accumulated in tumors to a greater extent than [^64^Cu]AMD-3100, although liver and kidney uptake remained significant and persistent [22]. Nevertheless, this early study supported the monocyclam structure of AMD-3465 as a scaffold for the design of novel CXCR4-targeting radioligands.

The positron emitting radionuclide, ^18^F (t_1/2_ = 109 min), can be produced in large quantity (>185 GBq) and at high molar activities, making it a more practical choice for CXCR4 probes if higher volume clinical use is to be achieved [23]. Recently we introduced, [^18^F]RPS-544, a derivative of the monocyclam AMD-3465, which demonstrated CXCR-dependent tumor uptake. However, accumulation of [^18^F]RPS-544 in the liver and intestines was substantial [24]. We hypothesized that further modification of the monocyclam structure may lead to a tracer with improved tumor uptake and/or normal tissue kinetics compared with [^18^F]RPS-544.

Our strategy was to create a library of fluorine-containing derivatives of AMD-3465, and select leading candidates for synthesis and in vitro and in vivo evaluation on the basis of an in silico screen. To generate structural diversity, we considered modifications and substitutions of the phenyl and pyridyl rings of the parent compound. We present herein a novel series of [^18^F]fluoroethyltriazolyl-containing monocyclam derivatives and highlight in particular [^18^F]RPS-534 and [^18^F]RPS-547, which demonstrate improved CXCR4-dependent tumor uptake and tumor-to-background ratios over [^18^F]RPS-544.

## 2. Results

### 2.1. Structural Design and Molecular Modeling

**Figure 1:**
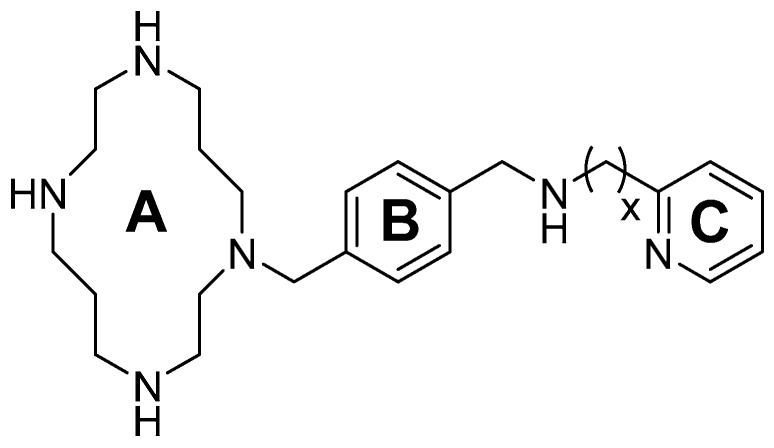
AMD-3465, the parent structure for derivatization. Fluorine was introduced by addition or substitutions of chemical moieties at rings B and C. The resulting library of structures were screened in silico against human CXCR4 (PDB ID: 3ODU) using Schrodinger.

AMD-3465 contains three rings in its structure: Ring A is a cyclam, ring B is a phenyl ring, and ring C is a pyridine ring (Figure 1). We elected to retain the cyclam moiety, and therefore considered the possibility of fluorination of rings B and C. Nucleophilic radiofluorination of pyridine rings is feasible only at the 2- and 4-positions. Therefore we considered other nitrogen-containing heteroaromatic rings at ring C as a method of introducing fluorine into the structure. We also examined the effect of transferring the fluorine label from the pyridine ring to ring B. This could be achieved either by direct fluorination or by the derivatization of ring B with an appropriate prosthetic group. The length of the linker between the rings B and C was also varied by insertion or deletion of a methylene group (X, Figure 1).

We created an initial library of over 200 fluorine-containing monocyclam derivatives and performed an in silico screen to identify those compounds with the highest predicted binding affinity. To give context to the docking scores, we included AMD-3100, AMD-3465, RPS-544, and 1c, a fluorobenzamide analogue of Pentixafor, in the in silico screen (Table 1). Pentixafor was synthesized by solution phase chemistry (Supplemental Data S002) to serve as a comparator for the monocyclam derivatives. The affinity of these ligands for CXCR4 ranges from approximately 5 nM to 650 nM [20,24], although some variation in affinity based on assay conditions is evident. Among the new structures we screened, those containing the 2-fluoroethyltriazole moiety consistently improved docking scores relative to other fluorine-containing prosthetic groups or direct incorporation of fluorine onto aromatic rings (Supplemental Data, Table S1). Interactions with residues Glu-32, Asp-87, Trp-94, His-113, Asp-187, and Arg-188 most contributed to high docking scores.

The 2-fluoroethyltriazole group was either directly conjugated to ring B at the C2 (RPS-534 and RPS-545) or C3 (RPS-533) positions, or used as ring C, as in RPS-546, RPS-547, and RPS-552 (Table 1). The docking scores of RPS-533 (−8.08 kcal/mol) and RPS-534 (−8.17 kcal/mol) were nearly identical to RPS-544 (−8.18 kcal/mol), but substantially higher than AMD-3465 (−7.01 kcal/mol). Relative to the strongest binders, the docking scores of ligands with the triazole as ring C, such as RPS-547 (−7.95 kcal/mol), were lower. Insertion (RPS-552; x = 2; −7.18 kcal/mol) or deletion (RPS-546; x = 0; −6.22 kcal/mol) of a methylene group induced larger decreases in docking score.

### 2.2. Radiosynthesis

Radiosynthesis of the candidate ligands was achieved by a Cu(I)-catalyzed click reaction between 2-[^18^F]fluoroethyl azide and the corresponding alkyne precursors. The precursors and ^19^F standards were prepared in multistep syntheses from commercially available building blocks. A full description of the synthesis and structural characterizations of these compounds is available in the Supplemental Data (S002).

2-[^18^F]fluoroethyl azide was synthesized and distilled in 0.5 mL DMSO with a decay-corrected radiochemical yield of 45.8 ± 15.4% (*n* = 5). A change in solvent to 0.1 mL MeCN in 0.4 mL DMSO increased the yield to 51.7 ± 9.5 % (*n* = 20) with concomitant transfer of a maximum of 100 µL MeCN to the second reaction vial. The radiochemical purity of the distillate was 91.9 ± 4.3%, with the only radiochemical impurity being a substance that co-eluted with the solvent front. No chemical impurities were evident by UV-Vis HPLC. The distillation was completed within 10 min and the rate was independent of the solvent system used. The average decay-corrected radiochemical yield of the click reaction was 85.3 ± 5.2% (*n* = 20), as determined by radioHPLC. The yield of [^18^F]RPS-533 was typically lower than the other ligands. Deprotection of the radiolabeled precursors was quantitative (>98%). Consequently, overall decay-corrected radiochemical yield was 38.6 ± 6.1% (*n* = 20), and total synthesis time was 2 h. Chemical and radiochemical purity exceeded 97% and molar activity was greater than 185 GBq/µmol.

### 2.3. In Vitro Experiments

Contrary to our expectations, the candidate ligands showed lower potency for binding CXCR4 than the relative docking scores would have suggested. The affinities of these ligands were in the range 200–1500 nM, representing a decrease of approximately 100-fold relative to RPS-544 (Table 2 and Supplemental Data Figure S7). To control for the possibility that the interaction between the ligands and [^68^Ga]Pentixafor is not a simple competitive one, possibly due to the peptide occupying a different part of the binding pocket, competition binding assays were also performed for RPS-533, RPS-534, and RPS-545 against [^18^F]RPS-544. The IC_50_ values determined in this assay were not significantly different from the values obtained against [^68^Ga]Pentixafor (*p* = 0.85). The binding curves were fitted to Hill plots with a slope of 1 (Supplemental Data, Table S2), with the exception of [^18^F]RPS-545, whose slope of 1.75 ± 0.33 suggests a positively cooperative binding interaction. K_d_ values were determined in a separate saturation binding experiment. The dissociation constants obtained were in the range 150–1700 nM (Supplemental Data, Figure S8), and the Hill slopes were consistent with those derived in the competition binding assay (Supplemental Data, Table S3).

The cell-associated activity of the compounds was generally low, with the exception of [^18^F]RPS-547 (6.7 ± 2.8 percent initial activity [%IA]), which was comparable to [^18^F]RPS-544, and [^18^F]RPS-534 (18.5 ± 3.7%IA), which was two-fold greater. In comparison, the cell-associated activity of [^68^Ga]Pentixafor was 39.7 ± 8.0 %IA. Negligible internalization of the radioligands was observed after 60 min incubation, and approximately 7% of the total cell-associated activity of [^18^F]RPS-534, corresponding to approximately 2%IA, was internalized after 135 min incubation (Supplemental Data, Figure S9). No correlation was observed between affinity for CXCR4 and uptake in PC3-CXCR4 cells in this group of ligands representing a narrow band of docking scores.

### 2.4. In Vivo Imaging and Biodistribution Studies

**Figure 2:**
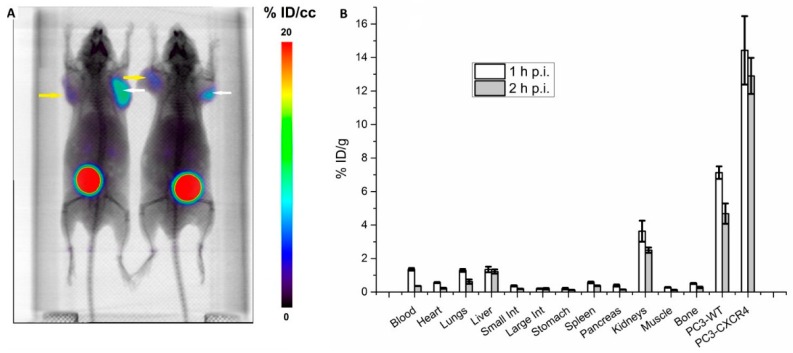
Tissue distribution of [^68^Ga]Pentixafor in male BALB/C nu/nu mice bearing subcutaneous bilateral PC3-WT (yellow arrow) and PC3-CXCR4 (white arrow) tumors. (**A**) Imaging of CXCR4 expression by microPET/CT. Mice were injected intravenously with 5.5 MBq [^68^Ga]Pentixafor and imaged at 1 h p.i. (**B**) Biodistribution of [^68^Ga]Pentixafor. Mice were injected with 1.85 MBq and sacrificed at 1 h (*n* = 5) and 2 h (*n* = 5) p.i.

[^68^Ga]Pentixafor accumulation was primarily evident in PC3-CXCR4 tumors and bladders at 1 h p.i. (Figure 2), with weaker signal also observed in PC3-WT tumors and kidneys. These findings were confirmed by our biodistribution studies. Maximum PC3-CXCR4 tumor activity was 14.6 ± 2.7 at 1 h p.i., with activity also most evident in PC3-WT tumors and kidneys. The difference in uptake between the PC3-CXCR4 and PC3-WT tumors was statistically significant (*p* < 0.001), resulting in a PC3-CXCR4-to-PC3-WT tumor uptake ratio of 2.0 ± 0.2 at 1 h p.i. and 2.8 ± 0.1 at 2 h p.i. By 2 h p.i. the PC3-CXCR4-to-blood ratio was 31.1 ± 0.1, and the ratio to all normal tissue except kidneys (5.3 ± 0.1) was greater than 10.

**Figure 3:**
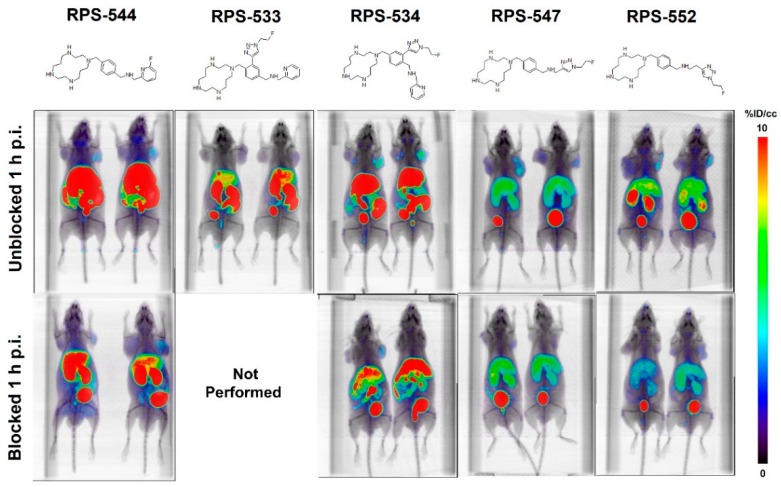
Maximum intensity projection µPET/CT images of male BALB/C nu/nu mice bearing PC3-WT xenograft tumors (left shoulder) and PC3-CXCR4 xenograft tumors (right shoulder). Images were prepared using Amide, and are horizontally inverted relative to the imaging position. The mice were administered intravenously with 7.4 MBq of each radioligand and imaged at 1 h p.i. Blocking was performed by co-injection of AMD-3100 (5 mg/kg).

The PET images of [^18^F]RPS-534, [^18^F]RPS-547, and [^18^F]RPS-552 at 1 p.i. were most promising, based on high uptake in PC3-CXCR4 tumors, low uptake in PC3-WT tumors, and good contrast (Figure 3). Co-injection of AMD-3100 confirmed CXCR4-specificity by reducing the tumor uptake of these ligands without substantially influencing clearance. Low level uptake in PC3-WT tumors is consistent with basal expression of CXCR4 in these cells [24]. The hepatic uptake of [^18^F]RPS-547 was lower than [^18^F]RPS-544. [^18^F]RPS-547 and [^18^F]RPS-552 cleared through the kidneys, leading to a reduction in liver uptake. Uptake of [^18^F]RPS-533 in the liver and intestines was lower than its regioisomer [^18^F]RPS-534, but tumor uptake also decreased. [^18^F]RPS-545 and [^18^F]RPS-546 were excluded from the animal studies due to low cell-associated activity, which reinforced the low affinities determined in vitro.

**Figure 4:**
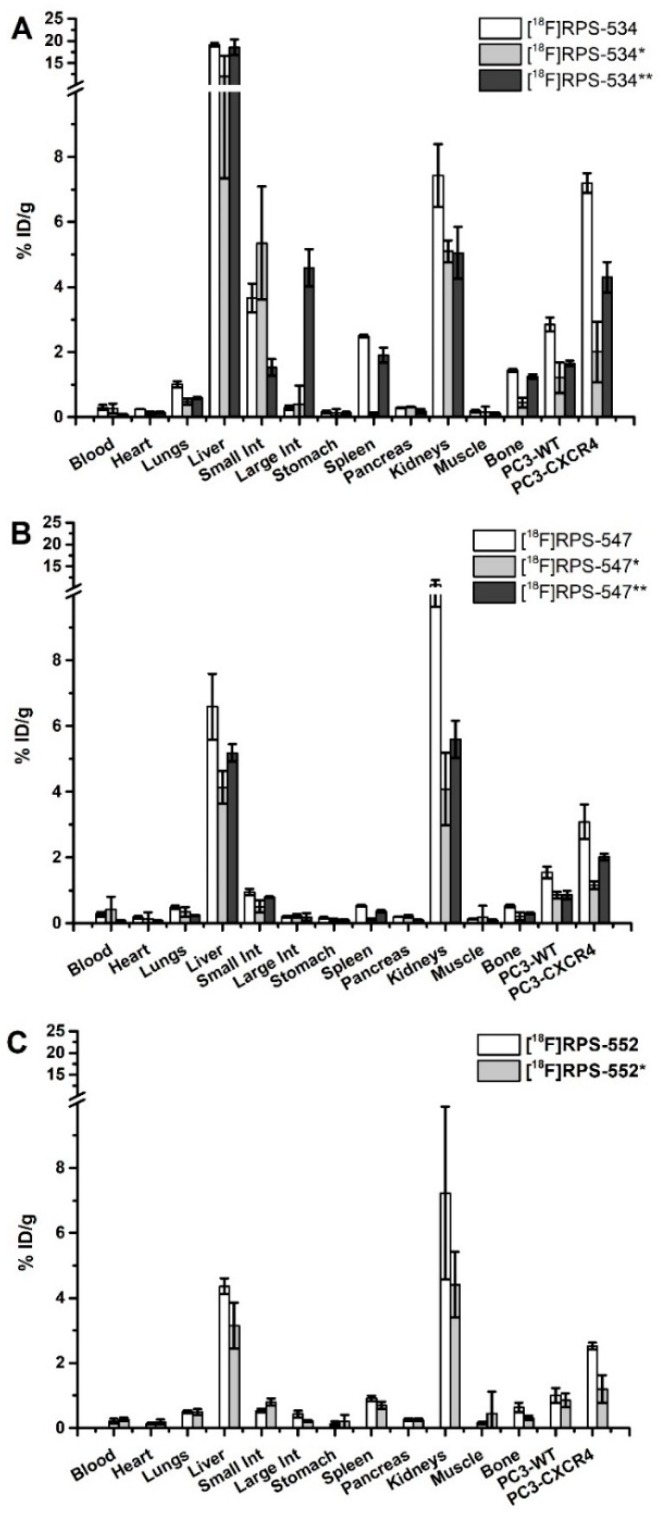
Biodistribution of (**A**) [^18^F]RPS-534, (**B**) [^18^F]RPS-547, and (**C**) [^18^F]RPS-552 at 1 h p.i. and 2 h (**) p.i. in male BALB/C mice bearing bilateral PC3-WT/PC3-CXCR4 xenograft tumors. Blocking (*) was performed at 1 h p.i. by co-injection of 5 mg/kg AMD-3100.

We observed significantly greater (*p* < 0.01) uptake of [^18^F]RPS-534, [^18^F]RPS-547, [^18^F]RPS-552 (Figure 4), and [^18^F]RPS-533 (Supplemental Data, Table S2) in PC3-CXCR4 tumors than PC3-WT tumors in the biodistribution studies at 1 h p.i. [^18^F]RPS-533 (1.93 ± 0.18 %ID/g) and [^18^F]RPS-552 (2.52 ± 0.11 %ID/g) did not reach the levels in PC3-CXCR4 tumors reported for [^18^F]RPS-544 [24]. The greatest uptake was observed for [^18^F]RPS-534 (7.20 ± 0.30 %ID/g), while uptake of [^18^F]RPS-547 (3.09 ± 0.52 %ID/g) was comparable to [^18^F]RPS-544. Uptake of [^18^F]RPS-534 and [^18^F]RPS-547 in PC3-CXCR4 tumors at 2 h p.i. decreased but remained significant (*p* < 0.001) with respect to PC3-WT tumors (Figure 4). The difference in uptake between PC3-CXCR4 and PC3-WT tumors was not significant at 1 h p.i. following co-administration of AMD-3100. Full tables of biodistribution values are available in the Supplemental Data (S006).

Although [^18^F]RPS-534 accumulated in the liver at 1 h p.i. (19.14 ± 0.42 %ID/g), leading to a lower tumor-to-liver ratio than [^18^F]RPS-533, [^18^F]RPS-547, and [^18^F]RPS-552, contrast to other tissues was greater (Table 3). Liver uptake decreased to 12.01 ± 4.67 %ID/g upon co-injection of AMD-3100, suggesting a possible component was related to CXCR4, but a similar degree of decrease in liver activity was not observed when [^18^F]RPS-547 was blocked with AMD-3100. As a consequence of decreased uptake in both PC3-WT and PC3-CXCR4 tumors following blocking, tumor-to-blood and tumor-to-muscle ratios decreased. These ratios were not significantly different for [^18^F]RPS-534, [^18^F]RPS-547 and [^18^F]RPS-552 following blocking (*p* > 0.38). [^18^F]RPS-533, [^18^F]RPS-547, and [^18^F]RPS-552 cleared primarily through kidneys (12.17 ± 0.78 %ID/g, 10.73 ± 1.10 %ID/g, and 7.23 ± 2.66 %ID/g, respectively). Therefore, tumor-to-kidney ratios for these ligands were one order of magnitude lower than for [^18^F]RPS-534. We also note the tumor-to-blood, tumor-to-muscle, and tumor-to-lung ratios of [^18^F]RPS-534 and [^18^F]RPS-547, which are considerably larger than those of [^18^F]RPS-544. Washout of [^18^F]RPS-534 and [^18^F]RPS-547 from PC3-CXCR4 tumors at 2 h p.i. ensured that these ratios did not change substantially, with the exception of tumor-to-blood, which continued to increase.

**Figure 5:**
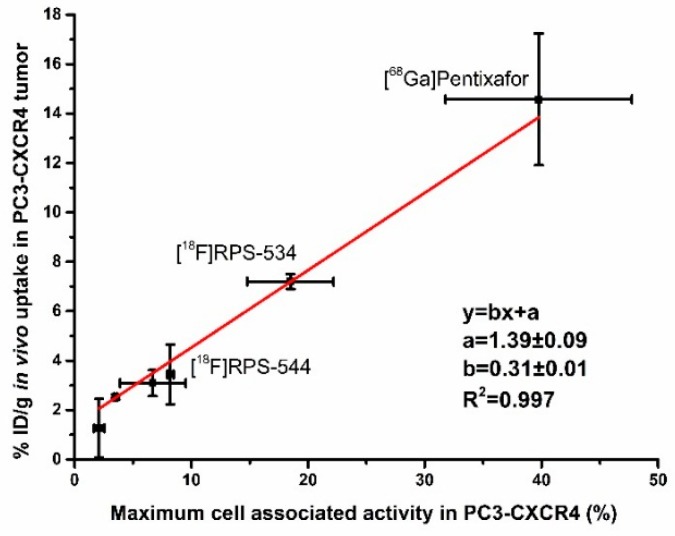
Correlation plot of the PC3-CXCR4 tumor uptake in vivo versus the PC3-CXCR4 cell-associated activity of the radioligands in vitro. Maximum cell-associated activity was calculated by subtracting the cell uptake at the highest non-radioactive ligand concentration of 10 µM from the cell uptake at the lowest non-radioactive ligand concentration of 10^−5^ µM. The equation of the linear fit and the correlation coefficient are reported.

**Figure 6:**
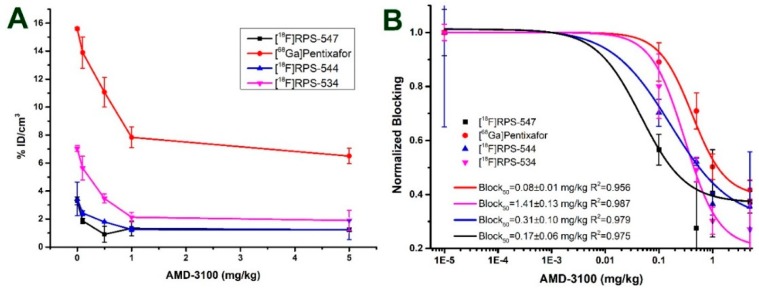
The effect of co-injection of 0.1, 0.5, 1, or 5 mg/kg of AMD-3100 on uptake of [^18^F]RPS-534, [^18^F]RPS-544, [^18^F]RPS-547, and [^68^Ga]Pentixafor in PC3-CXCR4 xenograft tumors. (**A**) Absolute tumor uptake as a function of the mass of AMD-3100. Uptake was quantified from µPET/CT images; (**B**) block_50_ values determined by binding curves fitted to the in vivo data. For fitting, negligible blocking was assumed for all compounds at 1 μg/kg AMD-3100. Based on the molar activity of the radioligands, the average amount of non-radioactive compound in the radiopharmaceutical injection was estimated to be 10 ng/kg.

Affinity for CXCR4 in vitro did not correlate to uptake of the radioligands in PC3-CXCR4 xenograft tumors. However, we found a linear correlation (R^2^ > 0.99) between total cell-associated activity and tumor uptake at 1 h p.i. (Figure 5). [^68^Ga]Pentixafor also fitted this relationship, suggesting that it is not specific to AMD-3465 and its structural derivatives. To create an alternative parameter for CXCR4 affinity that more closely correlated to tumor uptake in vivo, we titrated the tumor uptake of [^68^Ga]Pentixafor, [^18^F]RPS-534, [^18^F]RPS-544, and [^18^F]RPS-547 in µPET/CT images by co-injection of increasing masses of AMD-3100. Blocking doses of 0.08 ± 0.01 mg/kg and 0.17 ± 0.06 mg/kg of AMD-3100 reduced the uptake of [^18^F]RPS-547 and [^18^F]RPS-544, respectively, by 50% in PC3-CXCR4 tumors (Figure 6). However, 1.41 ± 0.13 mg/kg of AMD-3100 was necessary to reduce [^68^Ga]Pentixafor binding by the same amount. [^18^F]RPS-534 uptake was reduced by 50% with 0.31 ± 0.10 mg/kg of AMD-3100. By this measure, affinity for CXCR4 was in the order [^68^Ga]Pentixafor > [^18^F]RPS-534 > [^18^F]RPS-544 > [^18^F]RPS-547, and the correlation between PC3-CXCR4 tumor uptake and mass of AMD-3100 required to reduce uptake by 50% was linear (R^2^ > 0.95).

## 3. Discussion

The novel fluoroethyltriazole-containing derivatives of AMD-3465, [^18^F]RPS-534 and [^18^F]RPS-547, show high and specific uptake in CXCR4-positive tumors, which might be predicted based on their binding to PC3-CXCR4 cells. While we acknowledge the compounds prepared as part of this work represent a narrow chemical space, we observed a surprising lack of correlation between the docking score, CXCR4 affinity, and CXCR4 binding. On the basis of the docking score alone, [^18^F]RPS-544 (−8.18 kcal/mol) and [^18^F]RPS-534 (−8.17 kcal/mol) would be expected to exhibit similar CXCR4 affinities. However, the apparent affinity of [^18^F]RPS-534 is 30-fold lower. The Hill plots of both of these ligands support a one site, independent binding model, though we cannot rule out the possibility that multiple molecules of [^18^F]RPS-534 can bind CXCR4 independently. This might explain why, notwithstanding its weaker affinity, uptake of [^18^F]RPS-534 in PC3-CXCR4 xenograft tumors is 2-fold higher than [^18^F]RPS-544. Similarly, although both ligands can be partially displaced from PC3-CXCR4 tumors by co-injection of AMD-3100, the mass of AMD-3100 required to displace [^18^F]RPS-534 is greater than the mass required to displace [^18^F]RPS-544. An alternative explanation is that [^18^F]RPS-534 may also interact with related chemokine receptors such as CXCR7 to a greater extent than [^18^F]RPS-544. The affinity or binding interaction of [^18^F]RPS-534 for CXCR7 might differ to AMD-3100, which is an allosteric agonist [25], leading to incomplete blocking of tumor uptake. We are currently developing methods for determining the affinity of [^18^F]RPS-534, [^18^F]RPS-547, and [^18^F]RPS-552 for other chemokine receptors.

When we fitted a curve of the docking score versus IC_50_, we derived linear plot with R^2^ = 0.76. Our choice of AMD-3465 as a parent structure restricted our ligands to a relatively narrow band of docking scores. Within this narrow band, the docking score proved to have little predictive value. However, inclusion of RPS-510 (docking score = −2.13 kcal/mol; IC_50_ >10,000 nM in our assay) [24] as a negative control in the plot improved the fitting (R^2^ = 0.89; Figure S10). In this context, perhaps the greatest value of the in silico screen is to exclude structures with extremely poor affinity. Our experience suggests that structures whose docking score against CXCR4 exceeds −7.0 kcal/mol should be synthesized and comprehensively evaluated. We acknowledge a potential bias in favor of triazole-containing structures in our choice of candidate CXCR4 ligands due to our familiarity with synthetic and radiosynthetic strategies to producing these compounds. It is possible that some of the other monocyclam-containing analogues that we screened in silico might prove to be superior ligands in vivo. It is apparent that there is a lack of correlation between affinity and tumor uptake which cannot be attributed solely to compound pharmacokinetics. Given the PC3-CXCR4 tumor uptake of the radioligands in our bilateral tumor model, we found that the most relevant metric of evaluating these CXCR4 ligands in vitro is to measure the total cell associated activity (%IA) over 1 h.

The first reported ^18^F-labeled analogue of [^68^Ga]Pentixafor, [^18^F]AlF-NOTA-pentixather, was limited by relatively high plasma protein binding and in vivo defluorination [26]. The effect of these properties was to reduce the tumor-to-background contrast. To our knowledge, no ^18^F-labeled analogue of pentapeptide 1c [20] has been developed, perhaps due to the challenges of synthesizing the 4-[^18^F]fluorobenzoic acid synthon and selectively coupling it to the ornithine side chain. Therefore, small molecule CXCR4 ligands based around *para*-xylyl groups, such as AMD-3100, AMD-3465, and WZ811 [27], have served as the primary platforms for the derivatives of new fluorine-containing antagonists. [^18^F]-3 [28], alternatively reported as [^18^F]RPS-510 [24], a pyrimidine-pyridine amine compound, had low, but CXCR4-specific tumor uptake. [^18^F]RPS-544 showed improved tumor uptake compared to [^18^F]-3, but failed to address its pharmacokinetic deficiencies.

The tumor-to-background ratios of [^18^F]RPS-534 and [^18^F]RPS-547 are noticeably higher than those of [^18^F]RPS-544, but generally lower than those of [^68^Ga]Pentixafor. One exception is the tumor-to-blood ratio of [^18^F]RPS-534, 27.5 ± 0.4 at 1 h p.i., which is more than double the ratio of [^68^Ga]Pentixafor. Rapid clearance of [^18^F]RPS-534 from the blood is matched by its rapid clearance from the heart, which is captured by a tumor-to-heart ratio of 28.8 ± 0.3. These properties may be suitable for PET/CT imaging of myocardial infarction, an emerging application of CXCR4 molecular imaging [29]. Uptake in spleen and bone was evident for [^18^F]RPS-534 but not observed to the same extent with [^68^Ga]Pentixafor, [^18^F]RPS-547, or [^18^F]RPS-552. A blocking dose of 5 mg/kg AMD-3100 nearly eliminated radioactivity in these tissues, but only reduced activity in the tumor by approximately 70%. This may indicate that [^18^F]RPS-534 has a higher affinity than the other radioligands for native murine CXCR4, which is highly expressed in the bone and spleen [19]. These observations lead us to suggest that, at a minimum, the tumor-to-spleen and tumor-to-bone ratios of [^18^F]RPS-534 could improve upon translation to humans.

## 4. Materials and Methods

### 4.1. Schrodinger Molecular Modeling

All molecular modeling was performed using Schrodinger v. 2014-3 (Schrodinger, New York, NY, USA). Structures were docked against the available protein structure of the human CXCR4 chemokine receptor in complex with small molecule antagonist (3ODU). The small molecule antagonist was used to define the binding pocket and generate the docking grid for the modeling experiments. An Extra Precision (XP) docking study was performed on >200 fluorine-containing compounds structurally derived from AMD-3465. To calibrate the resulting docking scores, known CXCR4 ligands including AMD-3100, AMD-3465, cyclic pentapeptide 1c [20], and RPS-544 [24] were included in the modeling. The compounds were ranked by the docking score, reported in kcal/mol.

### 4.2. Synthesis of Compounds and Precursors

All precursors and non-radioactive standards were synthesized from commercially available building blocks purchased from Sigma Aldrich (St. Louis, MO, USA), unless otherwise indicated. Compounds were characterized by mass spectrometry and nuclear magnetic resonance (NMR) and were greater than 98% pure. A full description of synthetic procedures and compound characterizations is available in the Supplemental Data (S002).

### 4.3. Radiochemistry

#### 4.3.1. General Methods

Reactions were monitored by HPLC and radioHPLC using a PRP polymeric column (50 mm, Hamilton Company, Reno, NV, USA) and a Symmetry C18, 4.6 × 50 mm column (Waters Corporation, Milford, MA, USA). Chemical and radiochemical purity of the final compounds were determined by analytical (radio)HPLC. Compound identity was confirmed by co-injection with non-radioactive standards. Analytical and semi-preparative HPLC were performed on a dual pump Varian Dynamax HPLC (Agilent Technologies, Santa Clara, CA, USA) fitted with an Agilent ProStar 325 Dual Wavelength UV-Vis Detector, and radiochemical purity was determined using a NaI(Tl) flow count detector (Bioscan, Poway, CA, USA). UV absorption was monitored at 220 nm and 280 nm. A binary solvent system was used, with solvent A comprising H_2_O + 0.1% TFA and solvent B consisting of 90% *v*/*v* MeCN/H_2_O + 0.1% TFA. For analytical HPLC the following gradient was used at a flow of 2 mL/min: 0–2 min: 0% B; 2–8 min: 0–100% B; 8–9 min: 100% B; 9–10 min: 100–0% B. Semi-prep HPLC was performed using a μBondapak^®^ C18, 10 μm, 125 Å, 7.8 × 300 mm column (Waters, Milford, MA, USA). The following gradient was used at a flow of 10 mL/min: 0–1 min: 0% B; 1–16 min: 0–30% B; 16–18 min: 30–100% B; 18–19 min: 100% B; 19–20 min: 100–0% B.

#### 4.3.2. Radiosynthesis of [^68^Ga]Pentixafor

An ITG ^68^Ge/^68^Ga Generator was eluted using 4 mL 0.05M HCl and collected in a sterile 15 mL centrifuge tube. To this eluate, containing 555–1110 MBq (15–30 mCi) of ^68^GaCl_3_, was added 25 µL of a 1 mg/ml solution of pentixafor in DMSO. The pH was adjusted to pH 4–5 by addition of 80 µL of a 3N NaOAc solution. The reaction was then placed in an Eppendorf ThermoMixer^®^ (ThermoFisher Scientific, Waltham, MA, USA) at 95 °C and 600 rpm shaking for 15 min. A sample of the reaction was taken and analyzed by radio HPLC to confirm the labeling. Immediately after labeling was confirmed, the reaction was diluted with 9 mL H_2_O and the product was trapped on a Sep-Pak C18 Plus Lite (Waters, Milford, MA, USA) cartridge. The cartridge was washed with 5 mL normal saline solution (VWR International, Radner, PA, USA) and the product was eluted with 200 µL of a 50 % EtOH/saline solution, followed by 900 µL saline to produce a final injectable solution in 9% EtOH. A final QC HPLC was performed to assess purity.

#### 4.3.3. Radiosynthesis of ^18^F-Labeled Monocyclam Derivatives

No-carrier-added ^18^F^−^ was obtained from a (^18^O(p,n)^18^F) reaction by irradiating a liquid H_2_^18^O target in a 19 MeV EBCO Cyclotron (Vancouver, BC, Canada). H_2_^18^O containing [^18^F]F^−^ was passed through a Sep-Pak^®^ Light Waters Accell™ Plus QMA cartridge (Waters, Milford, MA, USA). The [^18^F]F^−^ was eluted using a solution of 10 mg kryptofix (K_222_) and 5 mg K_2_CO_3_ in 80% *v*/*v* MeCN/H_2_O. The eluate was dried at 100 °C under vacuum (25 mmHg) and a constant flow of N_2_ (99.999%, 30 mL/min flow). Drying was completed in approximately 20 min following two azeotropic cycles with MeCN (0.5 mL). To the dried residue was added 0.5 mL of a 10 mg/mL solution of 2-azidoethyltosylate in 20% *v*/*v* MeCN/DMSO, and the reaction was heated for 10 min at 80 °C. The reaction mixture was purified by distillation at 120 °C for 10 min. The distillate was collected in a clean V-shaped reaction vial containing 300 µL of DMF cooled to 0 °C. To this vial was added 100 µL of a 10 mg/mL solution of alkyne precursor in DMSO, followed by a pre-mixed solution of reduced Cu^+^ consisting of 50 µL of a 0.5 M aqueous CuSO_4_ solution, 50 µL of a 1.5 M aqueous sodium ascorbate solution, and 100 µL DMF. The reaction vial was heated for 10 min at 100 °C before it was diluted with H_2_O (10 mL) and passed through a pre-activated Sep-Pak^®^ Plus C18 Lite cartridge (Waters, Milford, MA, USA). The cartridge was washed with H_2_O (10 mL) and eluted with TFA (1 mL) into a clean V-shaped reaction vial. Deprotection was completed in 10 min at 100 °C, and the remaining TFA was evaporated under N_2_ flow. The dried residue was re-dissolved in 200 µL of 1M NH_4_OAc solution for final purification via semi-prep HPLC. The peak corresponding to the product was collected and diluted with H_2_O (20 mL). The product was trapped on a pre-activated Sep-Pak^®^ C18 Plus cartridge (Waters, Milford, MA, USA) and eluted with 0.5 mL EtOH (190 proof) followed by 5 mL of isotonic saline. The final product solution contained 9% *v*/*v* EtOH and a radioactivity concentration of 185–370 MBq/mL.

### 4.4. Cell Lines

The human prostate cancer cell line PC3-WT and the stably transduced human PC3-CXCR4 cell line were cultured as previously described [24].

### 4.5. Cell-Based Assays

#### 4.5.1. Determination of IC_50_ by Competition Binding Assay

PC3-CXCR4 cells were plated in 12-well plates at a density of 500,000 cells per well and incubated for 48 h at 37 °C/5% CO_2_. Prior to the binding assay the cells were incubated in fresh media and 370–555 kBq of [^68^Ga]Pentixafor or [^18^F]RPS-544 was added to each well. The corresponding concentration of [^68^Ga]Pentixafor in each well was 100 nM, and the concentration of [^18^F]RPS-544 was approximately 5 pM. Then non-radioactive ligands were added to give a final ligand concentration in the range 0.001–10,000 nM. A total of eight concentrations were studied, and each concentration was tested in triplicate. The cells were incubated for 60 min at 37 °C/5% CO_2_. The media was removed under suction, the cells were washed with cold PBS 1X and detached and transferred to tubes for counting in 1 M NaOH. The cells were counted in a Wizard^2^ Gamma Counter (PerkinElmer, Waltham, MA, USA). Non-specific binding was subtracted, and the IC_50_ values were determined by assuming a simple competitive binding interaction and fitting the data to a sigmoidal Hill plot in Origin 9.0 (OriginLab, Northampton, MA, USA). Potencies are expressed as IC_50_ ± standard deviation (Supplemental Data, Figure S7).

#### 4.5.2. Determination of Kd by Saturation Binding Assay

To determine the dissociation constants, saturation binding assays were performed in the PC3-CXCR4 cells. The cells were plated as described above, and approximately 5 nM of the radioligand was added to each well. Then the corresponding non-radioactive ligand was added to give a final concentration in the range 0.001–10,000 nM. A total of eight concentrations were tested, and each concentration was tested in triplicate. The samples were washed and counted as described above. Non-specific binding was subtracted, and Kd values were determined by performing a nonlinear one site binding curve fit with equation y = (B_max_ · x)/(Kd + x). Values are expressed as Kd ± standard deviation (Supplemental Data, Figure S8).

#### 4.5.3. Determination of Cell Binding and Internalization

Internalization studies were performed for [^18^F]RPS-534 and [^18^F]RPS-547 in a time dependent manner. PC3-CXCR4 cells were plated as described above in 12-well plates and incubated for 48 h prior to the experiment. The media was exchanged and [^18^F]RPS-534 or [^18^F]RPS-547 (10^−5^ μM; approximately 400 kBq) was added to each well. All experiments were performed in triplicate. The plates were incubated at 4 °C or 37 °C for 15, 30, 60, and 120 min. Then the media was removed under suction at the corresponding temperature, and the cells washed with 1 mL PBS. Cell surface-bound activity was collected by two successive 5 min incubations of 0.5 mL of a 50 mM glycine buffer (pH = 2.8) performed at 4 °C or 37 °C. The cells were then washed with 1 mL ice-cold PBS and detached with 1 mL ice cold 1 M NaOH. Detached cells were collected and counted along with the washes. An aliquot of the administered radioactivity (10% IA) was also counted and used for quantification. The activity found in the NaOH fractions of the 4 °C plates was subtracted from the activities of the fractions at 37 °C to correct for non-specific binding. Internalized activity was expressed as the fraction of activity in the NaOH fraction relative to the total activity added.

#### 4.5.4. Determination of Cell-Associated Activity

Maximum cell-associated activity was calculated by subtracting the cell uptake of the radioactive ligand at the highest non-radioactive ligand concentration of 10 µM (which we considered to be non-specific uptake/binding) from the cell uptake at the lowest non-radioactive ligand concentration of 10^−5^ µM.

### 4.6. Animal Studies

All animal studies were approved by the Institutional Animal Care and Use Committee of Weill Cornell Medicine (Protocol Number: 2015-0066) and were undertaken in accordance with the guidelines set forth by the USPHS Policy on Humane Care and Use of Laboratory Animals. Animals were housed under standard conditions in approved facilities with 12 h light/dark cycles. Food and water was provided ad libitum throughout the course of the studies. Hairless 6-to-8-week old male BALB/c inbred athymic nude mice (Jackson Laboratory, Bar Harbor, ME, USA) were inoculated bilaterally with 0.2 mL of a 1:1 mixture of 10^7^ PC3-CXCR4 cells in PBS and Matrigel (Corning, Tewksbury, MA, USA) subcutaneously in the left shoulder and 0.2 mL of a 1:1 mixture of 10^7^ PC3-WT cells in PBS and Matrigel subcutaneously in the right shoulder. Imaging and biodistribution studies were performed three weeks post-implantation, when tumors were approximately 100–300 mm^3^.

For the imaging studies, the tumor-bearing mice (*n* = 2/compound) were injected intravenously with 100 μL of a 9% *v*/*v* EtOH/saline solution containing approximately 3.7 MBq of the radioligand. Blocking of CXCR4-dependent tissue uptake of [^18^F]RPS-534, [^18^F]RPS-547, [^18^F]RPS-544, and [^68^Ga]Pentixafor was evaluated by co-injection of 0.1, 0.5, 1, or 5 mg/kg of AMD-3100 (*n* = 2 per compound per mass of AMD-3100). The animals were imaged under isoflurane at from 60–90 min p.i. in an Inveon μPET/CT (Siemens Medical Solutions, Malvern, PA, USA). Total image acquisition time was 30 min, and a CT scan was performed directly before the PET imaging for anatomical co-registration and attenuation correction. Images were reconstructed using the software provided by the vendor and post-reconstruction analysis, including decay correction, was performed using AMIDE (Open Source Software) prior to comparison. The images are laterally inverted relative to the imaging position, such that the PC3-CXCR4 tumor appears on the right shoulder and the PC3-WT tumor on the left shoulder. Tumor uptake was quantified by the following procedure: CT-aided regions of interest were drawn, and tissue concentration was determined by comparison with a 3 %ID/cm^3^ standard included in the field of view. Uptake was expressed as percent injected dose per centimeter cubed (%ID/cm^3^) ± standard deviation.

For the biodistribution studies, tumor-bearing animals (*n* = 5/compound/time point) were injected intravenously with 50–100 μL of a 9% *v*/*v* EtOH/saline solution containing approximately 1.1 MBq of the radioligand. Co-administration of AMD-3100 (5 mg/kg) was used to confirm CXCR4-dependent uptake of [^18^F]RPS-534, [^18^F]RPS-547, and [^18^F]RPS-552. The mice were euthanized at 1 or 2 h p.i. and tissues including blood, heart, lungs, liver, small intestine, large intestine, stomach (with contents), spleen, pancreas, kidneys, muscle, bone, PC3-WT tumors, PC3-CXCR4 tumors, and tail were collected. All organs were weighed and placed in test tubes for counting in a Wizard^2^ Gamma Counter (PerkinElmer, Waltham, MA, USA). For comparison, 1% injected activity standards were prepared and counted along with the tissue samples. The tails were counted to correct for any dose extravasation. Activity in each tissue was corrected for decay and for injected activity and expressed as percent injected dose per gram of tissue (%ID/g) ± standard error.

## 5. Conclusions

As predicted by their binding to PC3-CXCR4 cells, the fluoroethyltriazole-containing derivatives of AMD-3465, [^18^F]RPS-534, and [^18^F]RPS-547 showed high and specific uptake in CXCR4-positive tumors. Uptake of [^18^F]RPS-547 was comparable to [^18^F]RPS-544, a first-generation AMD-3465 derivative, but the improved clearance profile of [^18^F]RPS-547 led to higher tumor-to-normal tissue ratios. The tumor-to-normal tissue ratios of [^18^F]RPS-534, particularly tumor-to-blood, tumor-to-muscle, and tumor-to-lung, were even greater and comparable to [^68^Ga]Pentixafor. The chemical and pharmacokinetic properties of [^18^F]RPS-547 and especially [^18^F]RPS-534, including high yielding radiosynthesis, high tumor uptake, and good contrast to background, render these radiotracers promising candidates for imaging CXCR4 expression by PET.

## Figures and Tables

**Table 1:** Comparison of docking scores of six candidate CXCR4 ligands with known ligands with high affinity. Docking score was determined in an extra precision screen against human CXCR4 (PDB ID: 3ODU) using Schrodinger. The highest docking score is reported for each compound.

Name	Structure	Docking Score (kcal/mol)
RPS-545	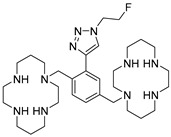	−8.51
AMD-3100	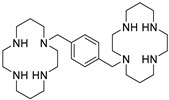	−8.50
1c	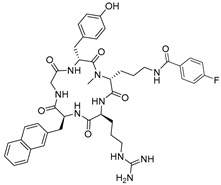	−8.29
RPS-544	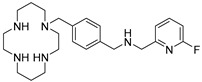	−8.18
RPS-534	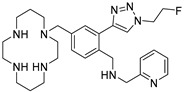	−8.17
RPS-533	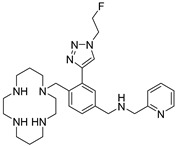	−8.08
RPS-547	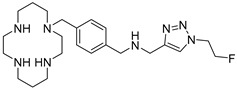	−7.95
RPS-552	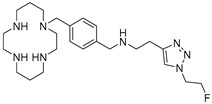	−7.18
AMD-3465	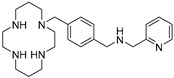	−7.01
RPS-546	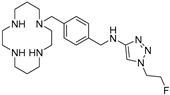	−6.22

**Table 2:** Comparison of key parameters in PC3-CXCR4 cells and PC3-CXCR4 xenograft tumors. IC_50_ was determined by competition binding against [^68^Ga]Pentixafor. PC3-CXCR4 cell and tumor uptake was determined at 1 h p.i. Cell-associated activity is defined as the percentage of initial activity (%IA) remaining after removal of media and washing of cells. Values are expressed as mean ± SEM.

Compound	IC_50_ (nM)	K_d_ (nM)	Cell-Associated Activity (%IA)	PC3-CXCR4 Tumor Uptake (%ID/g)
RPS-544	6.3 ± 0.8 (*n* = 6)	5.1 ± 1.0	8.2 ± 0.3	3.4 ± 1.2 *
RPS-533	356 ± 100 (*n* = 9)	366 ± 70	2.1 ± 0.4	1.93 ± 0.18
RPS-534	218 ± 38 (*n* = 18)	176 ± 30	18.5 ± 3.7	7.20 ± 0.30
RPS-545	398 ± 41 (*n* = 9)	515 ± 68	0.8 ± 0.1	n.d.
RPS-546	≈ 1500 (*n* = 3)	≈ 1700	1.2 ± 0.1	n.d.
RPS-547	601 ± 118 (*n* = 6)	261 ± 22	6.7 ± 2.8	3.09 ± 0.52
RPS-552	515 ± 68 (*n* = 6)	314 ± 48	3.5 ± 0.1	2.52 ± 0.11

* = Previously published data [24]. n.d. = Not determined.

**Table 3:** Tumor-to-tissue ratios for candidate CXCR4 ligands at 1 h p.i. (top) and 2 h p.i. (bottom). [^18^F]RPS-544 [24] and [^68^Ga]Pentixafor are included for comparison. The highest values for the [^18^F]fluorinated compounds are shown in bold for clarity.

1 h p.i.							
Compound	*PC3-CXCR4 Tumor-to-Tissue Ratio*						
	PC3-WT	Liver	Kidneys	Blood	Muscle	Bone	Lungs
*Pentixafor*	*2.0* ± *0.2*	*10.8* ± *0.2*	*4.0* ± *0.2*	*11.4* ± *0.2*	*56.1* ± *0.2*	*28.7* ± *0.2*	*11.8* ± *0.2*
RPS-544	**3.3 ± 1.3**	0.1 ± 0.1	0.1 ± 0.1	2.5 ± 0.4	11.1 ± 0.4	4.2 ± 1.2	2.0 ± 1.2
RPS-533	2.0 ± 0.4	0.5 ± 0.1	0.2 ± 0.1	6.9 ± 0.2	2.2 ± 0.3	1.4 ± 0.7	3.3 ± 0.4
RPS-534	**2.5 ± 0.4**	0.4 ± 0.2	**1.1 ± 0.4**	**27.5 ± 0.4**	**42.4 ± 0.1**	**5.7 ± 0.4**	**8.0 ± 0.4**
RPS-547	1.9 ± 0.3	**0.5 ± 0.2**	0.3 ± 0.2	10.1 ± 0.3	20.3 ± 0.3	**5.9 ± 0.8**	6.2 ± 0.8
RPS-552	**2.5 ± 0.3**	**0.6 ± 0.1**	0.3 ± 0.3	11.9 ± 0.1	16.7 ± 0.1	4.0 ± 0.2	5.0 ± 0.1
**2 h p.i.**							
*Pentixafor*	*2.8* ± *0.1*	*10.8* ± *0.1*	*5.3* ± *0.1*	*32.1* ± *0.1*	*101.2* ± *0.1*	*47.2* ± *0.1*	*17.1* ± *0.1*
RPS-544	**2.6 ± 0.3**	0.1 ± 0.1	0.1 ± 0.1	4.8 ± 0.1	6.7 ± 0.2	2.9 ± 0.3	1.7 ± 0.3
RPS-534	**2.5 ± 0.2**	0.2 ± 0.1	**0.9 ± 0.2**	**51.1 ± 0.2**	**38.8 ± 0.2**	3.5 ± 0.2	7.1 ± 0.2
RPS-547	2.2 ± 0.2	**0.4 ± 0.2**	0.3 ± 0.1	24.0 ± 0.2	11.0 ± 0.3	**6.3 ± 0.4**	**8.2 ± 0.4**

## Supplementary Materials

The following are available online: a selected summary of ligands included in the in silico screen (S001), and a full description of the synthesis of precursors and non-radioactive fluorinated standards (S002). Additional data are presented in Table S1: Fluorine-containing derivatives of AMD-3465, Table S2: Table of tissue activity values from biodistribution studies conducted in male BALB/c mice bearing bilateral PC3-WT/PC3-CXCR4 tumors; Figure S1: Synthesis of p-RPS-533 and RPS-533, Figure S2: Synthesis of p-RPS-545 and RPS-545, Figure S3: Synthesis of p-RPS-534 and RPS-534, Figure S4: Synthesis of p-RPS-547 and RPS-547, Figure S5: Synthesis of p-RPS-552 and RPS-552, Figure S6: Synthesis of p-RPS-546 and RPS-546, Figure S7: Determination of IC_50_ in a competition assay vs [^68^Ga]Pentixafor in PC3-CXCR4 cells, Figure S8: Determination of dissociation constants (Kd) in PC3-CXCR4 cells, Figure S9: Internalization of [^18^F]RPS-534 and [^18^F]RPS-547 in PC3-CXCR4 cells, Figure S10: Linear plot of docking score and IC_50_.
