# Supplementary material for: [18F]Fluoroethyltriazolyl Monocyclam Derivatives as Imaging Probes for the Chemokine Receptor CXCR4
Source: Molecules. 2019 Apr 24;24(8):1612. doi: 10.3390/molecules24081612 (PMC6514812; doi:10.3390/molecules24081612)

SUPPLEMENTARY MATERIALS

for

[^{18}F]fluoroethyltriazolyl monocyclam derivatives as imaging probes for the chemokine receptor CXCR4

Alejandro Amor-Coarasa, James M. Kelly, Pradeep K. Singh, Shashikanth Ponnala, Anastasia Nikolopoulou, Clarence Williams, Jr., Yogindra Vedvyas, Moonsoo M. Jin, J. David Warren, John W. Babich

Table of Contents

S001. XP Docking Scores for CXCR4 Binders	2
S002. Syntheses of Precursors and Standards	13
S003. Inhibition Binding Assays	36
S004. Saturating Binding Assays	37
S005. Internalization Experiments	39
S006. Tissue Biodistribution	40
S007. Correlation Between Docking Score and IC_{50}	41
S008. NMR Spectroscopic Data	42

S001. XP Docking Scores for CXCR4 Binders

Table S1. Fluorine-containing derivatives of AMD-3465. Docking score was determined in an extra precision screen against human CXCR4 (PDB ID: 3ODU) using Schrodinger. The highest docking score is reported for each compound.

Name or No.	Compound	XP Docking Score (kcal/mol)
**RPS-534**	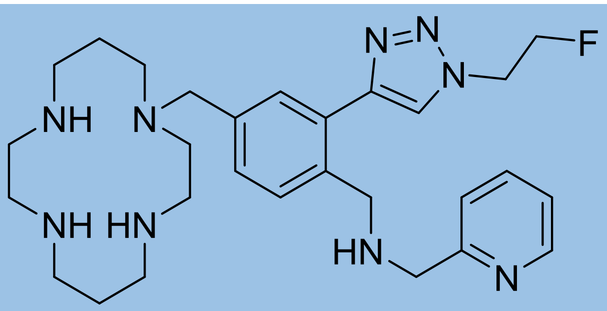	-8.17
**1**	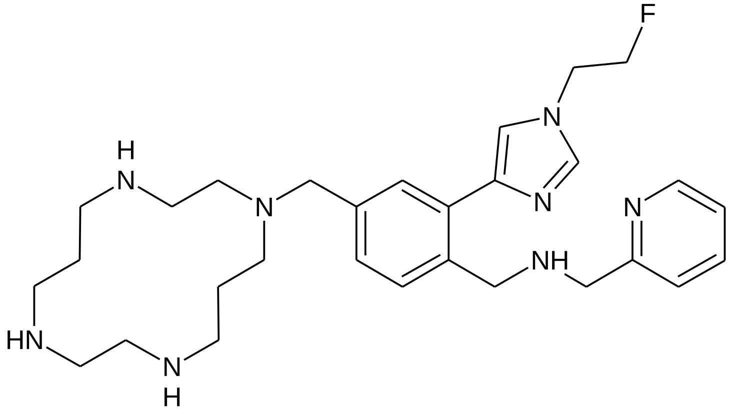	-8.06
**2**	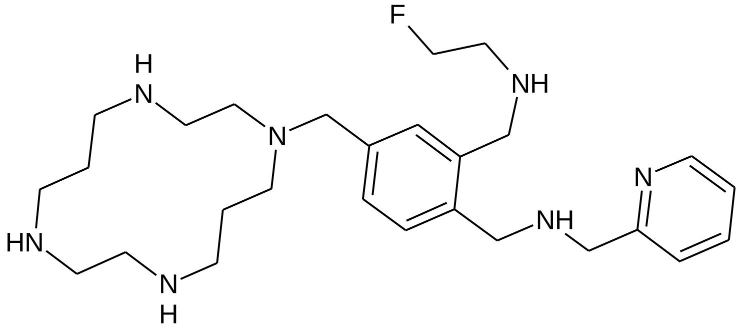	-7.62

**3**	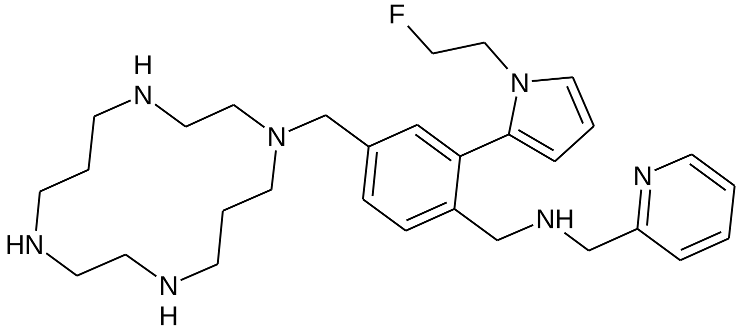	-7.41
**4**	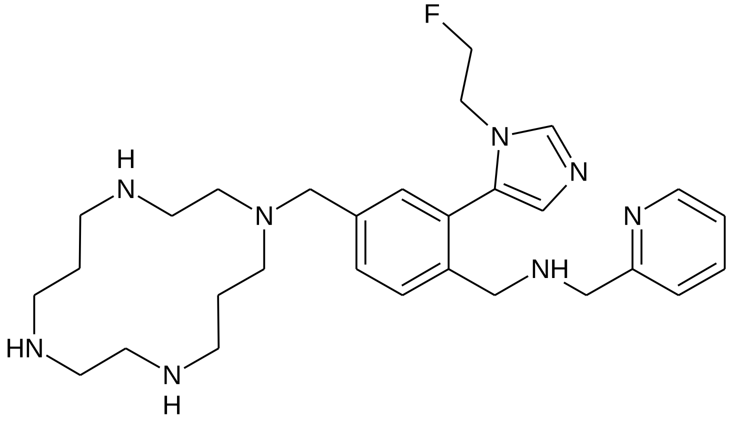	-5.93
**5**	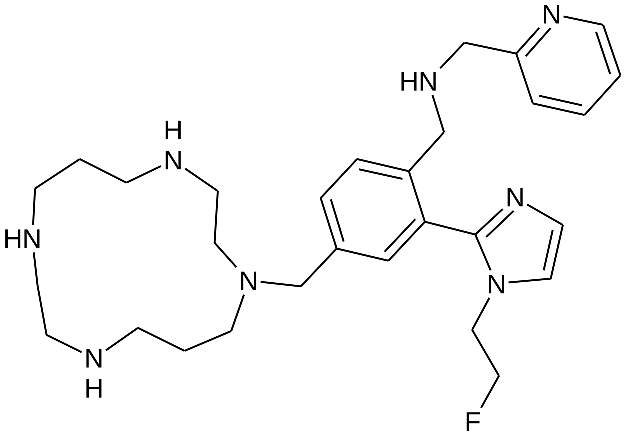	-6.74
**6**	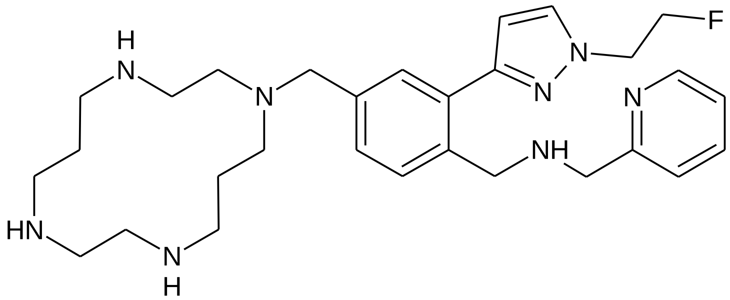	-6.87

**7**	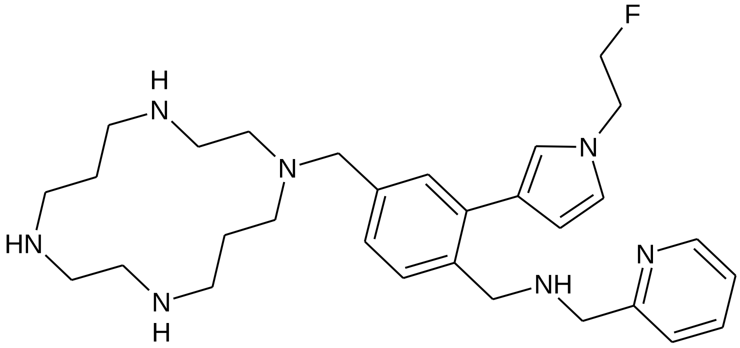	-7.19
**8**	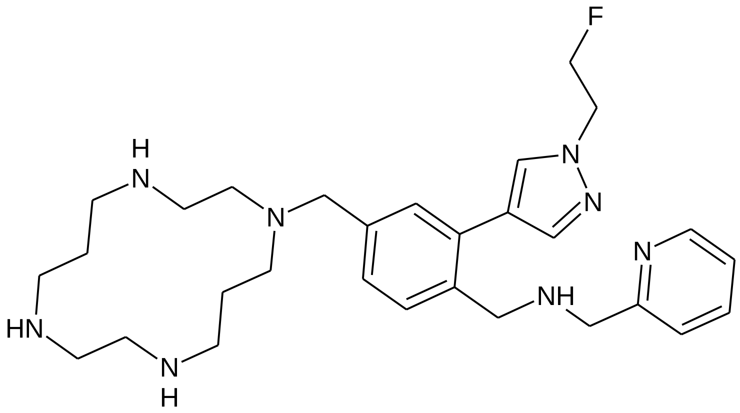	-7.12
**9**	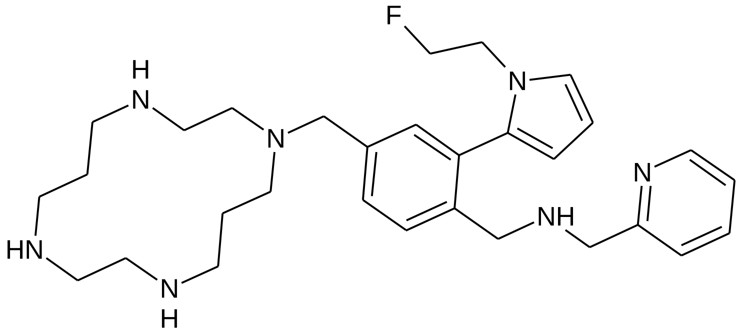	-6.97
**10**	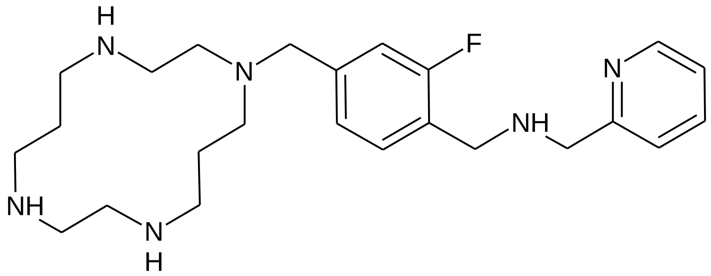	-6.85

**11**	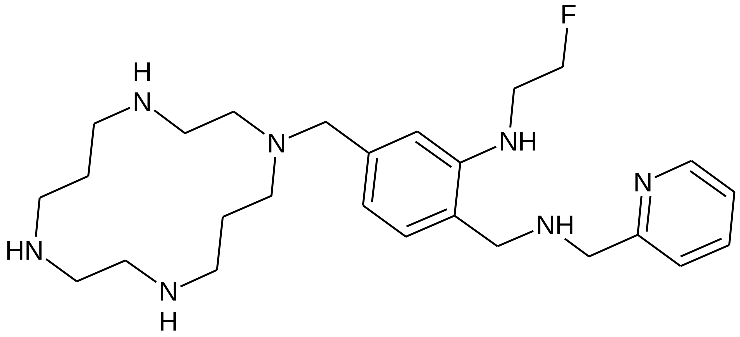	-6.75
**RPS-533**	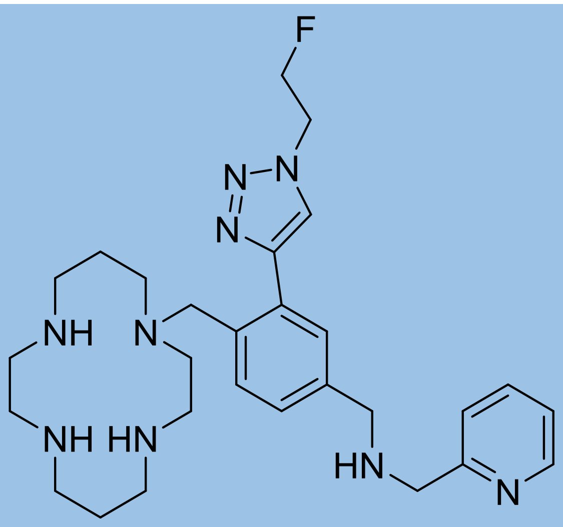	-8.08
**1**	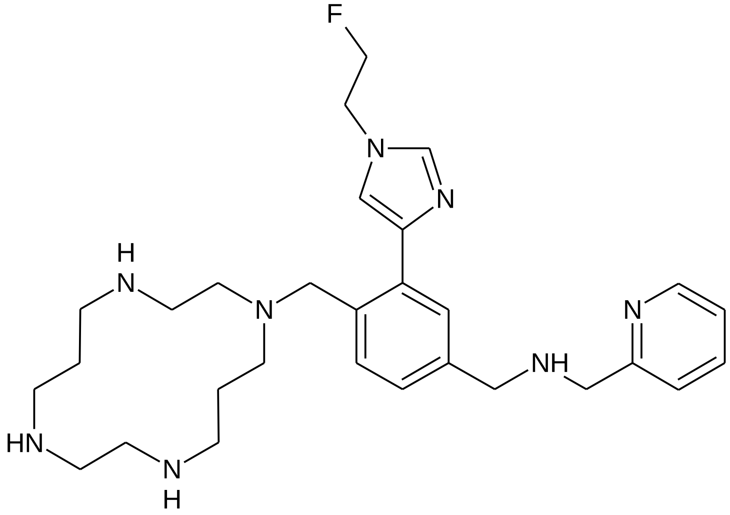	-8.06

**2**	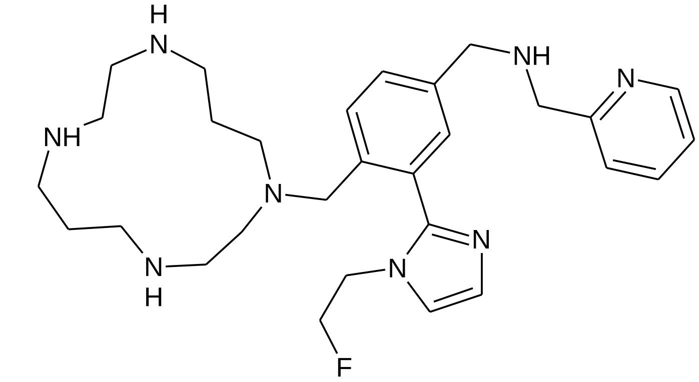	-8.02
**3**	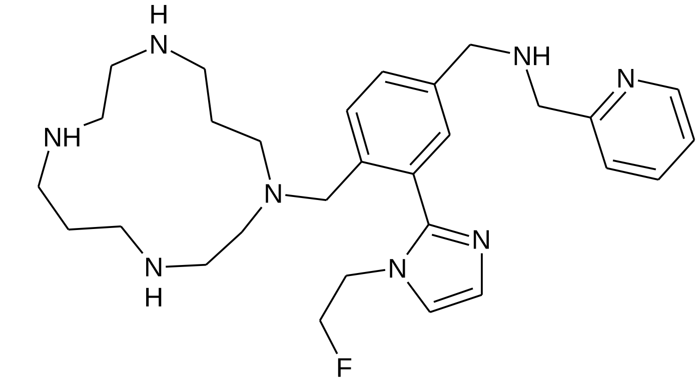	-8.02
**4**	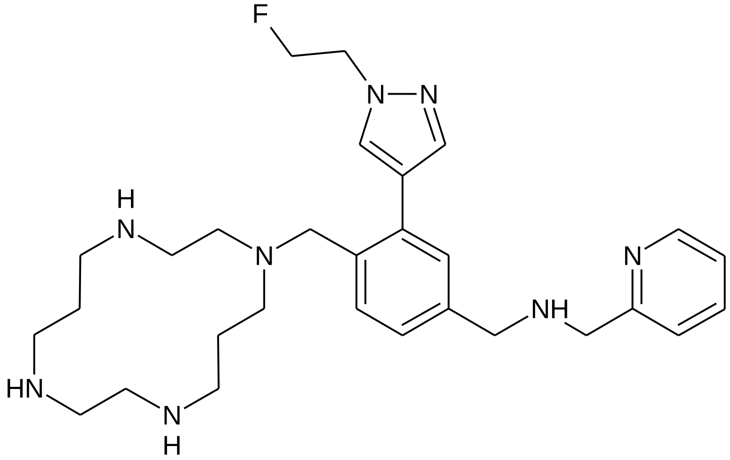	-7.79

**5**	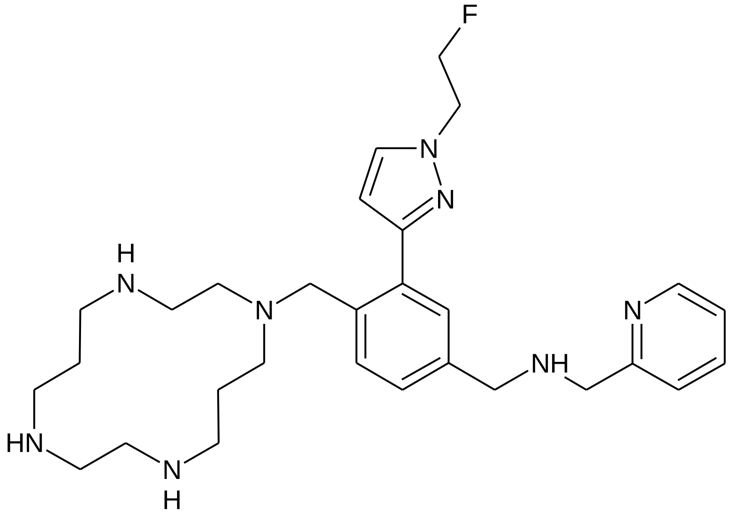	-7.41
**6**	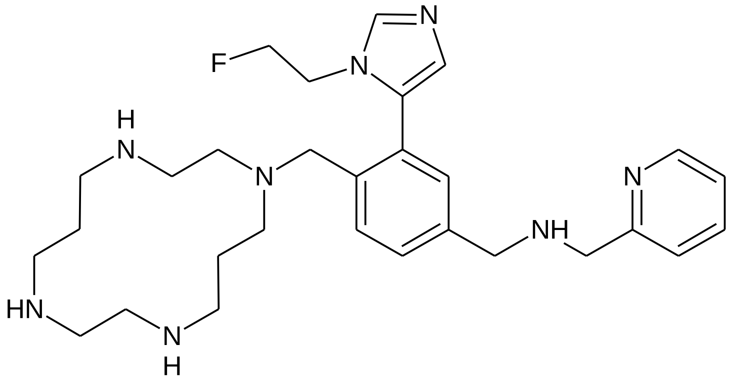	-7.19
**7**	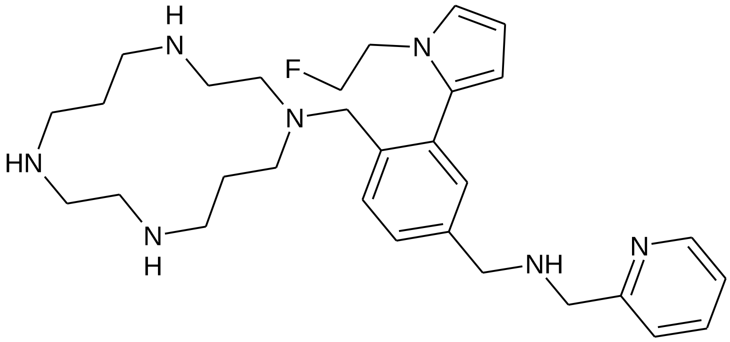	-7.12

**8**	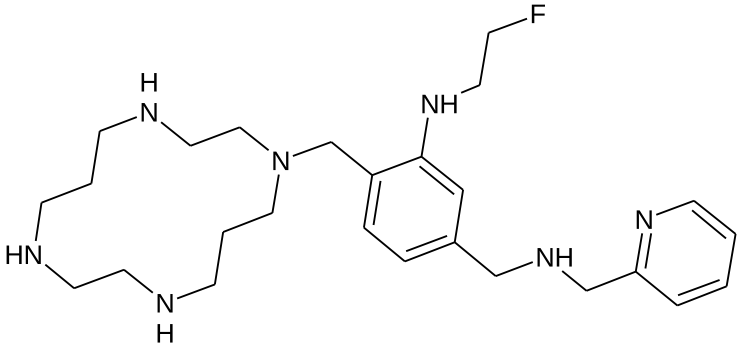	-6.93
**9**	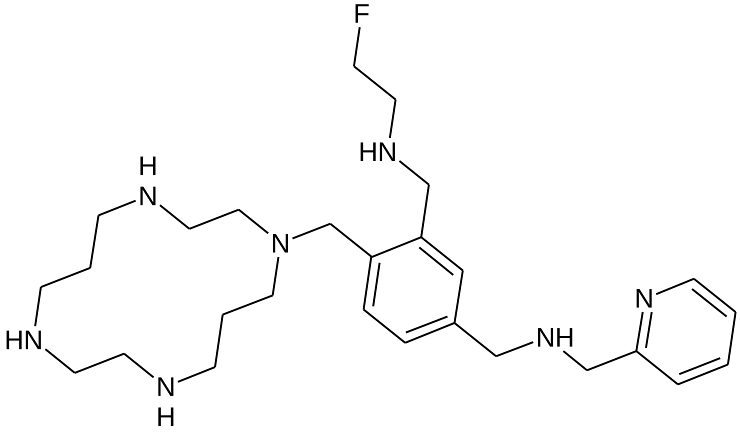	-6.72
**10**	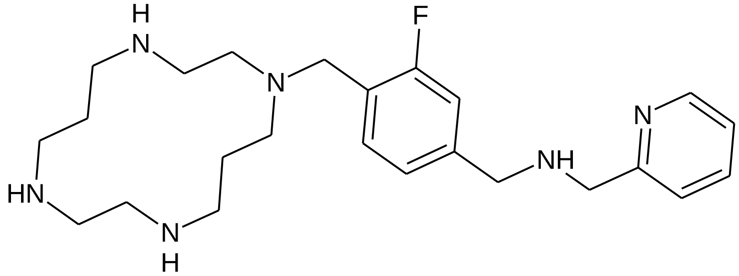	-6.22
**RPS-547**	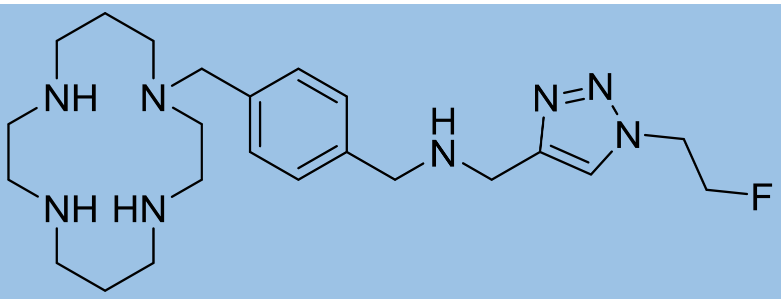	-7.95

1

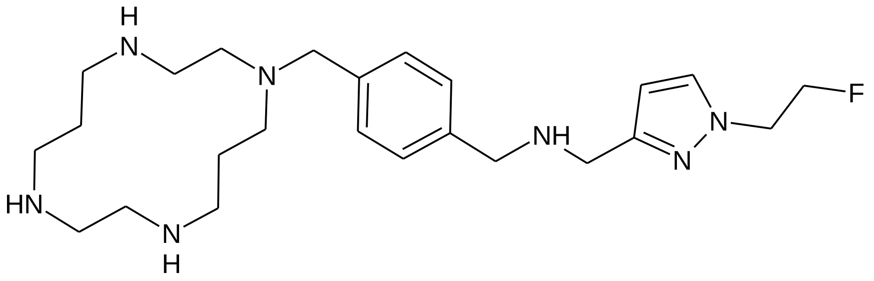

-7.02

2

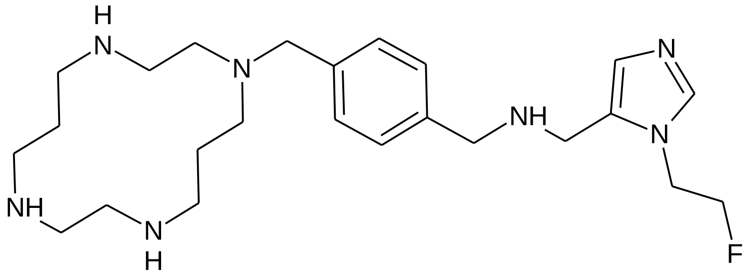

-6.55

3

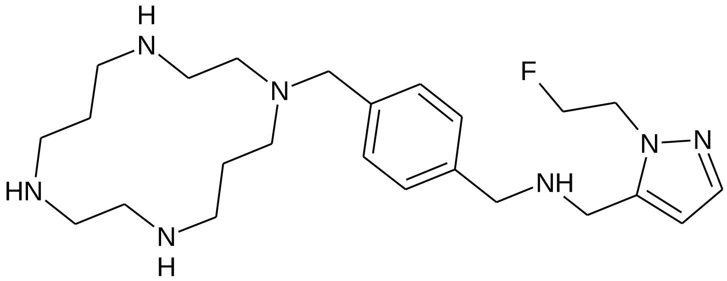

-6.19

4

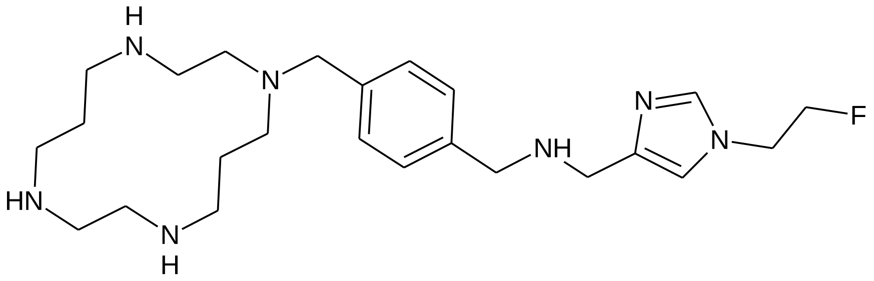

-5.96

5

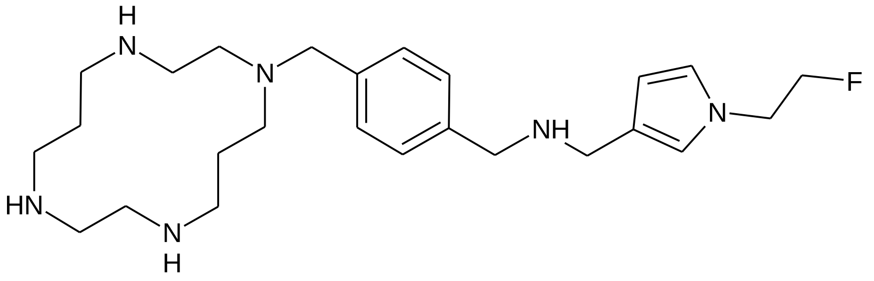

-5.85

**6**	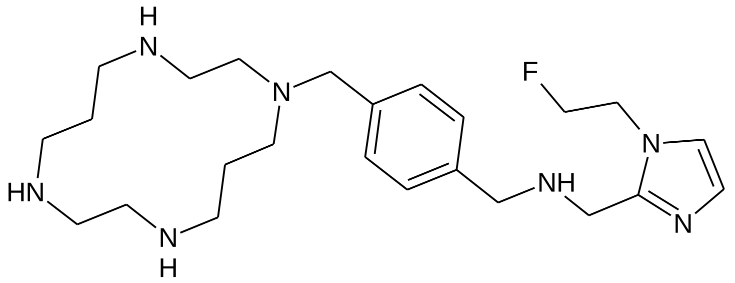	-5.65
**7**	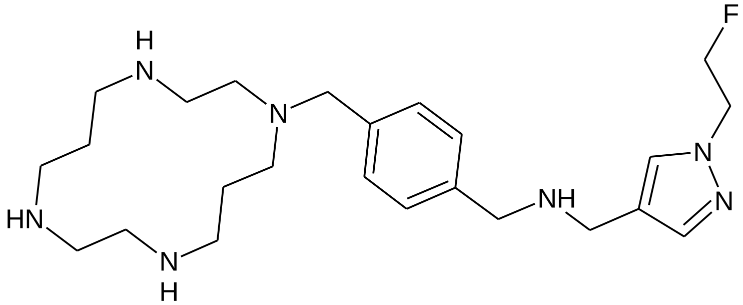	-5.44
**8**	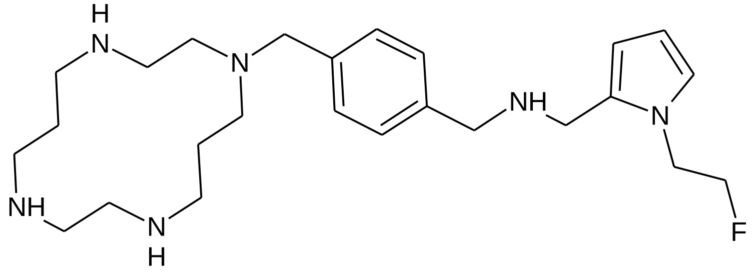	-5.44
**RPS-552**	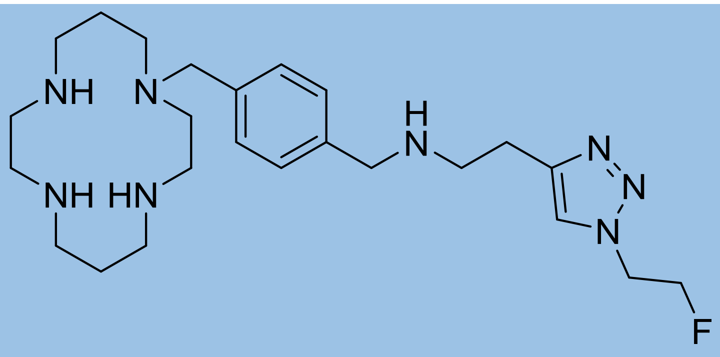	-7.18
**1**	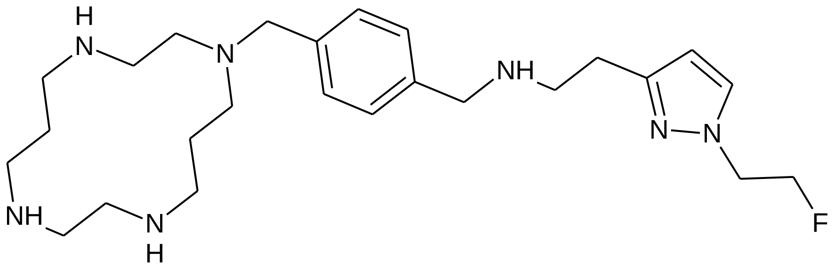	-7.91

**2**	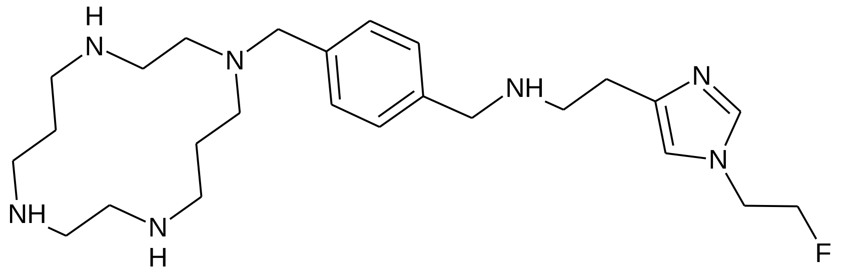	-7.61
**3**	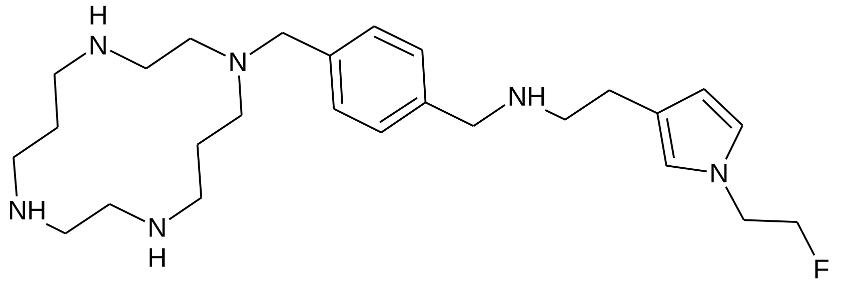	-7.04
**4**	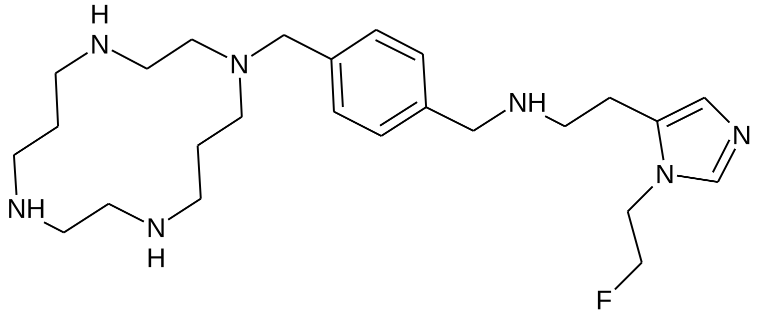	-6.60
**5**	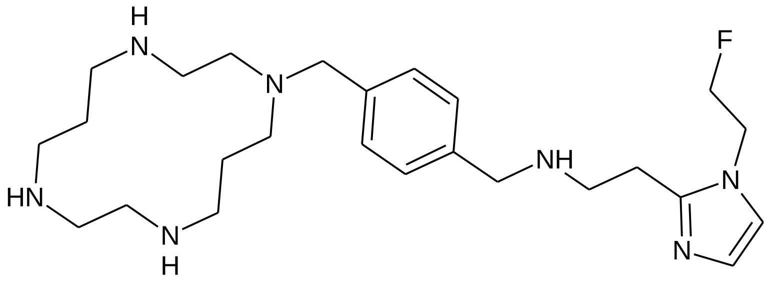	-5.89
**6**	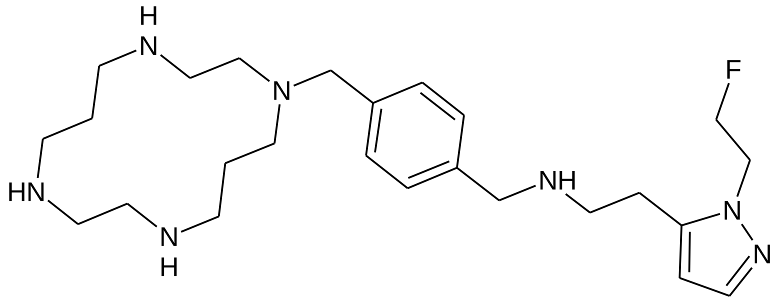	-5.67

**7**	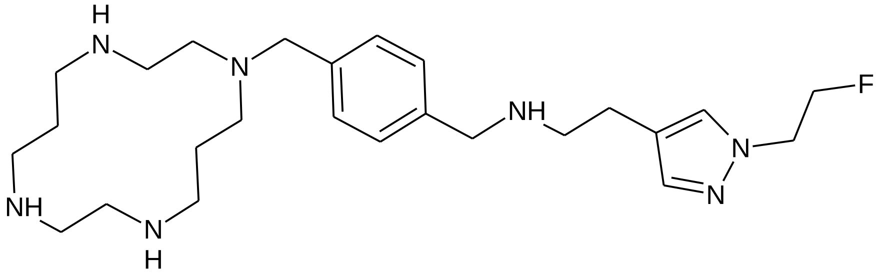	-5.23

S002. Syntheses of Precursors and Standards

General

All commercially available materials were used as received unless otherwise indicated. 4-(bromomethyl)-3-iodobenzoic acid [1] and 2-fluoroethyl azide [2] were synthesized according to literature procedures. All reactions were carried out under an atmosphere of argon in an oven-dried round bottom flask with magnetic stirring, unless otherwise noted. Reactions were monitored by UPLC. HPLC purifications were performed using a Waters AutoPure HPLC/MS system equipped with XBridge OBD prep C18 5μm (19 x 150 mm) column and SQD2 mass spectrometer. A 10-minute gradient of 5% – 95% acetonitrile (0.1% formic acid) in water (0.1% formic acid) was used as mobile phase. All NMR spectra were recorded on Bruker DRX-500 spectrometer (500 MHz for ^{1}H and 125 MHz for ^{13}C). Chemical shifts, δ, are reported in ppm, with the residual solvent resonance as internal standard. NMR data are reported as following: chemical shift (multiplicity s = singlet; d = doublet; t = triplet; q = quartet; m = multiplet; br = broad, coupling constant in Hz, and integration).

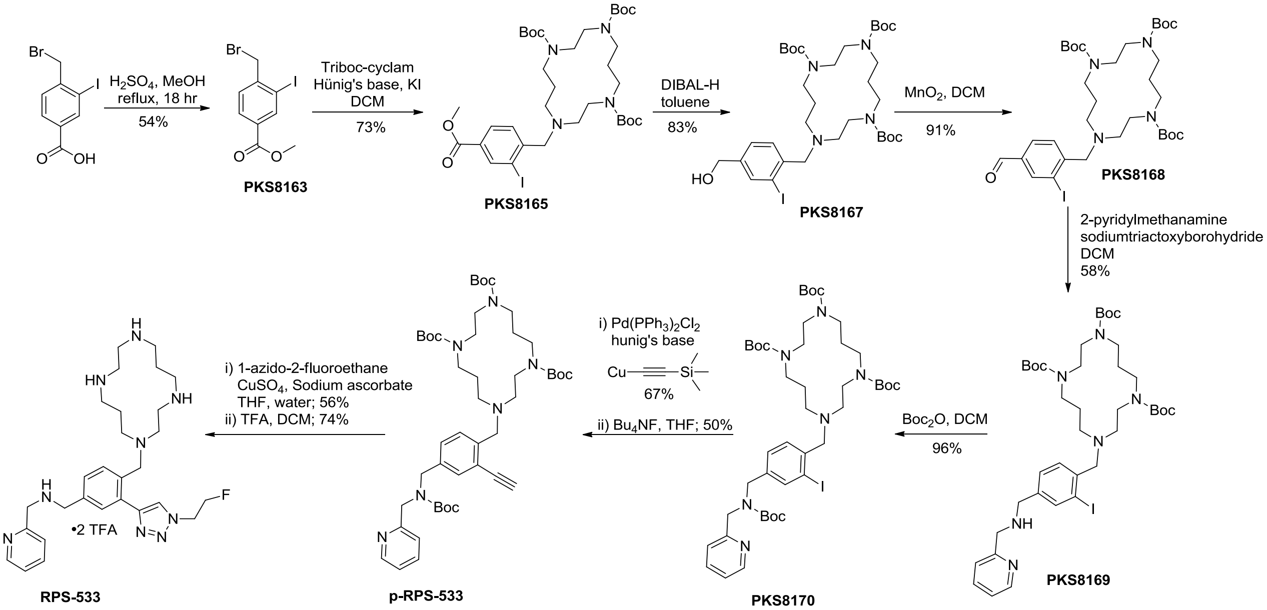

Figure S1. Synthesis of p-RPS-533 and RPS-533.

PKS8163: 4-(bromomethyl)-3-iodo-benzoic acid (8.00 g, 23.5 mmol) was dissolved in methanol (50.0 mL) and H_2SO_4 (2.50 mL, 46.93 mmol) was added to the solution. The mixture was refluxed under overnight. The solvent was removed, and the mixture was purified by Combi-Flash (silica gel; slow 0 - 10% ethyl acetate in hexanes gradient) to give product (3.4 g, 54%) as white solid. ^{1}H NMR (500 MHz, $CDCl_3$) δ 8.51 (d, *J* = 1.7 Hz, 1H), 7.99 (dd, *J* = 8.0, 1.7 Hz, 1H), 7.53 (d, *J* = 8.0 Hz, 1H), 4.60 (s, 2H), 3.92 (s, 3H).

PKS8165: methyl 4-(bromomethyl)-3-iodo-benzoate (**PKS8163**; 547 mg, 1.54 mmol) and triBoc-cyclam (700 mg, 1.40 mmol) were dissolved in DMF (4 mL). To the solution DIPEA (490 μL, 2.80 mmol) and potassium iodide (116 mg, 700 μmol) were added. The mixture was stirred at ambient temperature for 24 h. The reaction mixture was diluted with water and extracted twice with ethyl acetate, and the

combined organic layer was washed with brine, dried over anhydrous sodium sulfate and evaporated. The crude product was purified by Combi-Flash (silica gel; 0 – 70% gradient of ethyl acetate in hexanes) to give **PKS8165** (1.05 g, 97%) as a white solid. ^{1}H NMR (500 MHz, $CDCl_3$) δ 8.46 (d, *J* = 1.7 Hz, 1H), 7.95 (d, *J* = 8.0 Hz, 1H), 7.56 – 7.41 (m, 1H), 3.91 (s, 3H), 3.61 (s, 2H), 3.50 – 3.21 (m, 12H), 2.76 – 2.58 (m, 2H), 2.57 – 2.35 (m, 2H), 1.96 – 1.81 (m, 2H), 1.80 – 1.66 (m, 2H), 1.51 – 1.30 (m, 27H).

PKS8167: **PKS8165** (925 mg, 1.19 mmol) was dissolved in dry toluene (6 mL) in an argon flushed 50 mL round bottom flask. The solution was cooled to -78 °C and diisobutylaluminium hydride (1.0 M in hexane, 2.97 mmol, 3 mL) was added dropwise. The reaction mixture was slowly warmed to 0 °C. Reaction was quenched with water (200 μL) and 1N NaOH (3 mL) was added. The resulting mixture was diluted with ethyl acetate and stirred for 30 min. Then the mixture was filtered through Celite and evaporated. The crude product was purified by Combi-Flash to give **PKS8167** (740 mg, 83%) as a white solid. ^{1}H NMR (500 MHz, $CDCl_3$) δ 7.82 (s, 1H), 7.34 (d, *J* = 8.0 Hz, 1H), 7.29 (d, *J* = 8.0 Hz, 1H), 4.62 (d, *J* = 5.0 Hz, 2H), 3.56 (s, 2H), 3.48 – 3.19 (m, 12H), 2.76 – 2.58 (m, 2H), 2.54 – 2.34 (m, 2H), 1.93 – 1.78 (m, 2H), 1.76 – 1.66 (m, 2H), 1.51 – 1.30 (m, 27H).

PKS8168: To a solution of **PKS8167** (717 mg, 960 μmol) in DCM (15 mL) was added manganese dioxide (334 mg, 3.84 mmol). The mixture was stirred at ambient temperature overnight and was then heated to reflux for 4 h. After completion of the reaction, the mixture was filtered through Celite, evaporated and dried under vacuum to give **PKS8168** as a white solid (695 mg, 97%) that was used without further purification. ^{1}H NMR (500 MHz, $CDCl_3$) δ 9.90 (s, 1H), 8.29 (s, 1H), 7.80 (d, *J* = 8.0 Hz, 1H), 7.59 (d, *J* = 8.0 Hz, 1H), 3.63 (s, 2H), 3.52 – 3.20 (m, 12H), 2.78 – 2.60 (m, 2H), 2.60 – 2.36 (m, 2H), 1.97 – 1.81 (m, 2H), 1.77 – 1.66 (m, 2H), 1.54 – 1.29 (m, 27H).

PKS8169: A mixture of **PKS8168** (685 mg, 920 μmol) and 2-pyridylmethanamine (100 μL, 966 μmol) in DCM (10 mL) was stirred at ambient temperature for 2 h and then sodium triacetoxyborohydride (585 mg, 2.76 mmol) was added. The resulting mixture was stirred at ambient temperature for 4 h. Excess reagent was quenched with aqueous $NaHCO_3$, the layers were separated and the aqueous layer was extracted with dichloromethane. The combined organic layers were dried over anhydrous sodium sulfate and evaporated to give **PKS8169** as a colorless gum (which turned into a fluffy solid under vacuum) that was used without further purification. ^{1}H NMR (500 MHz, DMSO-d_6) δ 8.50 – 8.46 (m, 1H), 7.82 (d, *J* = 1.5 Hz, 1H), 7.77 – 7.72 (m, 1H), 7.43 (d, *J* = 7.8 Hz, 1H), 7.35 – 7.29 (m, 2H), 7.25 – 7.22 (m, 1H), 3.75 (s, 2H), 3.67 (s, 2H), 3.51 (s, 2H), 3.47 – 3.14 (m, 12H), 2.62 – 2.53 (m, 2H), 2.46 – 2.28 (m, 2H), 1.85 – 1.72 (m, 2H), 1.69 – 1.55 (m, 2H), 1.43 – 1.18 (m, 27H).

PKS8170: Di-*tert*-butyl dicarbonate (364 μL, 1.58 mmol) was added to a solution of **PKS8169** (663.0 mg, 792 μmol) in DCM (10 mL). After stirring for 5 min, triethylamine (329 μL, 2.38 mmol) was added and the reaction mixture was stirred at ambient temperature for 3 h. The solvent was evaporated, and the crude residue was purified by Combi-Flash (silica gel; 0 - 10 % methanol in dichloromethane) to give **PKS8170** as a white solid. ^{1}H NMR (500 MHz, DMSO-d_6) δ 8.53 – 8.49 (m, 1H), 7.78 – 7.73 (m, 1H), 7.69 (s, 1H), 7.35 (d, *J* = 7.1 Hz, 1H), 7.27 (dd, *J* = 7.5, 4.9 Hz, 1H), 7.25 – 7.15 (m, 2H), 4.49 – 4.41 (m, 2H), 4.41 – 4.32

(m, 2H), 3.50 (s, 2H), 3.46 – 3.09 (m, 12H), 2.62 – 2.54 (m, 2H), 2.45 – 2.25 (m, 2H), 1.85 – 1.72 (m, 2H), 1.69 – 1.57 (m, 2H), 1.45 – 1.17 (m, 36H).

p-RPS-533: Ethynyl(trimethyl)silane (91 μL, 640 μmol) and triethyl amine (1.5 eq.) were added to a suspension of copper(I) iodide (41 mg, 214 μmol) in THF (1 mL) and the mixture was stirred at ambient temperature for 1 h. In parallel, **PKS8170** (200 mg, 213 μmol), *trans*-dichlorobis(triphenylphosphine)palladium (II) (74.9 mg, 107 μmol) and triethylamine (1.5 eq.) were dissolved in THF (2 mL) and stirred at ambient temperature for 1 h in a microwave vessel flushed with argon to give a yellow suspension. The copper acetylide solution was added to the microwave vessel to give a clear solution. The mixture was heated to 120 °C under microwave for 1 h. The mixture was cooled, the solvent was evaporated, and the crude residue was purified by Combi-Flash (silica gel; 0 - 10 % gradient of methanol in dichloromethane) to give the silyl phenylacetylide (130 mg, 67%) as a white solid. The solid (125 mg, 137 μmol) was dissolved in THF (3 mL). The solution was cooled to 10 °C and tetrabutylammonium fluoride (1.0 M in THF) (138 μmol, 140 μL) was added. The solution was warmed to ambient temperature and stirred for 1 h. The solvent was evaporated, and crude residue was purified by HPLC to give **p-RPS-533** (57 mg, 50%) as a white solid. ^{1}H NMR (500 MHz, DMSO-d$_6$) δ 8.53 – 8.48 (m, 1H), 7.79 – 7.72 (m, 1H), 7.39 (d, *J* = 8.0 Hz, 1H), 7.32 – 7.14 (m, 4H), 4.46 (s, 2H), 4.42 – 4.31 (m, 3H), 3.62 (s, 2H), 3.44 – 3.07 (m, 12H), 2.60 – 2.53 (m, 2H), 2.42 – 2.25 (m, 2H), 1.86 – 1.70 (m, 2H), 1.68 – 1.53 (m, 2H), 1.46 – 1.17 (m, 36H).

RPS-533: Copper sulfate pentahydrate (0.5 M, 66 μL) and sodium ascorbate (1.5 M, 22 μL) were mixed under argon atmosphere and stirred for 20 min until the solution turned dark orange. In another round bottom flask **p-RPS-533** (14.0 mg, 17 μmol) and 1-azido-2-fluoro-ethane (0.33 M, 102 μL) were dissolved in DMF (1 mL). To this mixture was added the Cu(I) reagent, and the resulting reaction was stirred at ambient temperature overnight. The mixture was diluted with dichloromethane and passed through a basic alumina plug. The filtrate was evaporated, and crude product was purified by HPLC to give a white solid (8.7 mg, 56%). The solid (6.0 mg, 6.5 μmol) was dissolved in DCM (0.5 mL) and the solution was cooled to 0 °C. Trifluoroacetic acid (0.5 mL) was added to the solution and the mixture was slowly warmed to ambient temperature. After completion of reaction, excess solvent and TFA were evaporated and crude was purified by HPLC to give **RPS-533** (5.8 mg, 74%) as the TFA salt and a white solid. ^{1}H NMR (500 MHz, DMSO-d$_6$) δ 9.63 (br, 2H), 8.64 (d, *J* = 4.9 Hz, 1H), 8.43 (s, 1H), 7.88 (t, *J* = 7.7 Hz, 1H), 7.75 (s, 1H), 7.57 (d, *J* = 6.9 Hz, 2H), 7.49 (d, *J* = 7.9 Hz, 1H), 7.44 (dd, *J* = 7.6, 5.0 Hz, 1H), 4.98 – 4.92 (m, 1H), 4.89 – 4.83 (m, 2H), 4.83 – 4.78 (m, 1H), 4.33 (s, 2H), 4.30 (s, 2H), 3.89 (s, 2H), 3.20 – 2.79 (m, 14H), 2.65 – 2.52 (m, 2H), 1.87 – 1.59 (m, 4H). ^{19}F NMR (471 MHz, DMSO-d$_6$) δ -224.6 (tt, *J* = 46.9, 28.1 Hz).

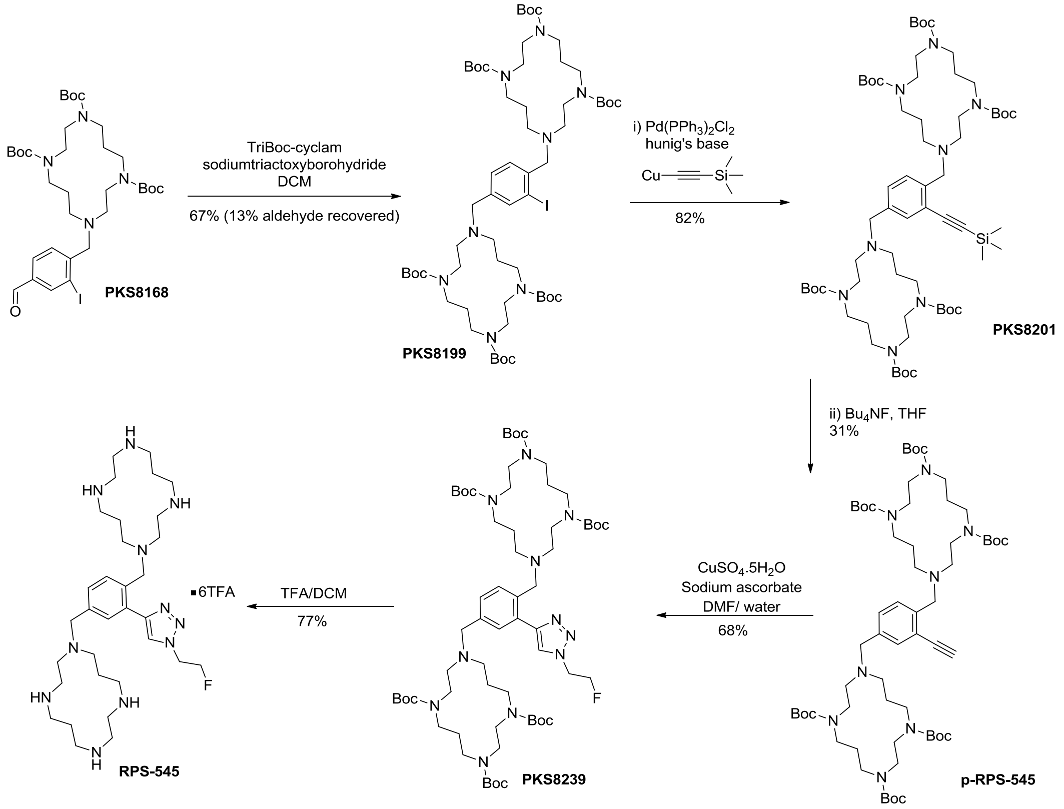

Figure S2. Synthesis of p-RPS-545 and RPS-545.

PKS8199: A mixture of **PKS8168** (206 mg, 277 μmol) and triBoc-cyclam (138 mg, 277 μmol) in DCM (5 mL) was stirred at ambient temperature for 2 h and then sodium triacetoxyborohydride (176 mg, 830 μmol) was added. The mixture was stirred at ambient temperature overnight. Excess reagent was quenched with aqueous $NaHCO_3$, the layers were separated and the aqueous layer was extracted with dichloromethane. The combined organic layers were dried over anhydrous sodium sulfate and evaporated, and the crude residue was purified by Combi-Flash (silica gel; ethyl acetate in hexane) to give **PKS8199** (230 mg, 67%) as a colorless gum which turned into a fluffy solid under vacuum. ^{1}H NMR (500 MHz, DMSO-d$_6$) δ 7.75 (s, 1H), 7.32 (d, *J* = 7.8 Hz, 1H), 7.23 (d, *J* = 7.8 Hz, 1H), 3.50 (s, 2H), 3.44 (s, 2H), 3.40 – 3.03 (m, 24H), 2.63 – 2.56 (m, 2H), 2.54 – 2.48 (m, 2H), 2.44 – 2.15 (m, 4H), 1.92 – 1.71 (m, 4H), 1.61 (s, 4H), 1.47 – 1.16 (m, 54H).

PKS8201: Ethynyl(trimethyl)silane (52 μL, 366 μmol) and triethyl amine (1.5 eq.) were added to a suspension of copper(I) iodide (23 mg, 122 μmol) in THF (1 mL) under argon atmosphere and the mixture was stirred at ambient temperature for 1 h. **PKS8199** (150 mg, 122 μmol), *trans*-dichlorobis(triphenylphosphine)palladium (II) (43 mg, 61 μmol) and triethylamine (1.5 eq.) were dissolved in THF (2 mL) and stirred at ambient temperature for 1 h in a microwave vessel flushed with argon to give a yellow suspension. The copper acetylide reagent was added to the microwave vessel to give a clear solution. The mixture was heated to 120 °C under microwave for 1 h. The mixture was cooled, the solvent was evaporated, and the crude residue was purified by Combi-Flash (silica gel; 0 - 10%

gradient of methanol in dichloromethane) to give **PKS8201** (120 mg, 82%). ^{1}H NMR (500 MHz, DMSO-d$_6$) δ 7.44 – 7.40 (m, 1H), 7.34 (d, *J* = 6.8 Hz, 1H), 7.18 (s, 1H), 3.65 (s, 2H), 3.46 (s, 2H), 3.39 – 2.97 (m, 24H), 2.64 – 2.52 (m, 4H), 2.45 – 2.10 (m, 4H), 1.79 (s, 4H), 1.56 (s, 4H), 1.47 – 1.17 (m, 54H), 0.22 (d, J = 6.4 Hz, 9H).

p-RPS-545: **PKS8201** (120 mg, 100 μmol) was dissolved in THF (3 mL) and cooled to 10 °C. Tetrabutylammonium fluoride (1.0 M in THF) (100 μL, 100 μmol) was added, and the solution was warmed to ambient temperature and stirred for 1 h. The solvent was evaporated, and the crude residue was purified by HPLC to give **p-RPS-545** (35 mg, 31%) as white solid. ^{1}H NMR (500 MHz, DMSO-d$_6$) δ 7.40 – 7.31 (m, 2H), 7.24 (d, *J* = 7.9 Hz, 1H), 4.33 (s, 1H), 3.63 (s, 2H), 3.47 (s, 2H), 3.42 – 3.01 (m, 24H), 2.63 – 2.54 (m, 2H), 2.54 – 2.51 (m, 2H), 2.40 – 2.18 (m, 4H), 1.90 – 1.72 (m, 4H), 1.69 – 1.51 (m, 4H), 1.45 – 1.19 (m, 54H).

PKS8239: Copper sulfate pentahydrate (0.5 M, 35 μL) and sodium ascorbate (1.5 M, 12 μL) were mixed under an argon atmosphere and stirred for 20 min while the solution turned dark orange. In another round bottom flask **p-RPS-545** (9.8 mg, 9 μmol) and 1-azido-2-fluoro-ethane (0.33 M, 53 μL) were dissolved in DMF (1 mL). The Cu(I) reagent was added to the mixture, resulting in a dull green color, and the reaction was stirred at ambient temperature overnight. The mixture was diluted with dichloromethane and passed through a basic alumina plug. The filtrate was evaporated, and crude residue was purified by HPLC to give **PKS8239** (10 mg, 95%) as a white solid. ^{1}H NMR (500 MHz, DMSO-d$_6$) δ 8.34 (s, 1H), 7.56 – 7.41 (m, 2H), 7.24 (d, *J* = 7.8 Hz, 1H), 4.97 – 4.89 (m, 1H), 4.87 – 4.78 (m, 2H), 4.78 – 4.71 (m, 1H), 3.69 (s, 2H), 3.53 (s, 2H), 3.48 – 3.02 (m, 24H), 2.60 – 2.53 (m, 2H), 2.54 – 2.46 (m, 2H), 2.39 – 2.17 (m, 4H), 1.88 – 1.69 (m, 4H), 1.67 – 1.49 (m, 4H), 1.45 – 1.13 (m, 54H).

RPS-545: **PKS8239** (10 mg, 8 μmol) was dissolved in DCM (0.5 mL) and the solution was cooled to 0 °C. Trifluoroacetic acid (0.5 mL) was added to the solution and the mixture was slowly warmed to ambient temperature. After completion of reaction, the excess solvent and TFA were evaporated and the crude residue was purified by HPLC to give **RPS-545** (9.6 mg, 77%) as the TFA salt and a colorless gum. ^{1}H NMR (500 MHz, DMSO-d$_6$) δ 8.48 (s, 1H), 7.46 – 7.40 (m, 2H), 7.34 (d, *J* = 7.9 Hz, 1H), 7.22 (br, 6H), 4.99 – 4.76 (m, 4H), 3.84 (s, 2H), 3.74 (s, 2H), 3.20 – 2.92 (m, 16H), 2.86 – 2.51 (m, 16H), 2.00 – 1.91 (m, 2H), 1.84 – 1.63 (m, 6H). ^{13}C NMR (126 MHz, DMSO-d$_6$) δ 145.9, 134.7, 133.6, 131.4, 131.1, 130.8, 130.0, 124.4, 81.9 (d, *J* = 168.5 Hz), 53.9, 52.7, 50.3 (d, *J* = 19.1 Hz), 50.0, 49.7, 49.3, 48.4, 48.2, 47.7, 45.1, 44.9, 44.2, 44.1, 43.9, 43.7, 42.9, 24.8, 22.0. ^{19}F NMR (471 MHz, DMSO-d$_6$) δ -224.9 (tt, *J* = 46.8, 28.1 Hz).

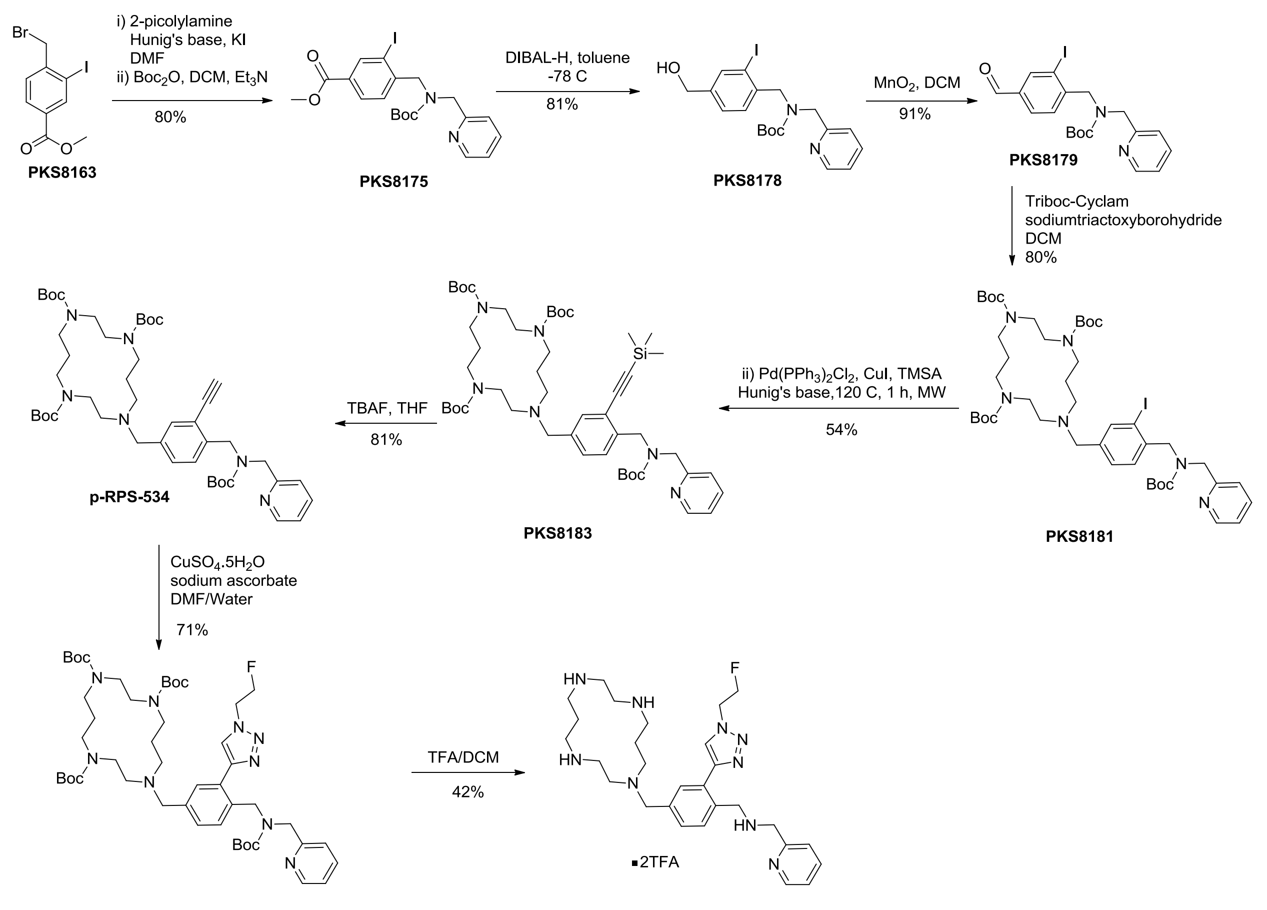

Figure S3. Synthesis of p-RPS-534 and RPS-534.

PKS8175: **PKS8163** (800 mg, 2.25 mmol) and 2-pyridylmethanamine (422 μL, 4.10 mmol) were dissolved in DMF (4 mL). To the solution were added Hünig's base (716 μL, 4.10 mmol) and potassium iodide (170 mg, 1.02 mmol). The mixture was stirred at ambient temperature for 24 h, at which point di-*tert*-butyl dicarbonate (1.41 mL, 6.15 mmol,) was added. The mixture was stirred for an additional 4 h before it was diluted with water and extracted twice with ethyl acetate. The combined organic layers were washed with brine, dried over anhydrous sodium sulfate and evaporated. The crude residue was purified by Combi-Flash (silica gel; 0 - 70% gradient of ethyl acetate in hexanes) to give **PKS8175** (950 mg, 96%) as a colorless gum. ^{1}H NMR (500 MHz, $CDCl_3$) δ 8.52 (d, *J* = 4.7 Hz, 1H), 8.45 (d, *J* = 1.7 Hz, 1H), 8.03 – 7.93 (m, 1H), 7.78 – 7.67 (m, 1H), 7.33 – 7.13 (m, 3H), 4.74 – 4.45 (m, 4H), 3.91 (s, 3H).

PKS8178: **PKS8175** (125 mg, 259 μmol) was dissolved in dry toluene (4 mL) in an argon flushed 25 mL round bottom flask. The solution was cooled to -78 °C and DIBAL-H (1M in hexane) (650 μL, 650 μmol) was added dropwise. The reaction mixture was slowly warmed to 0 °C. The reaction was quenched with water (200 μL) and 1N NaOH (1 mL), diluted with ethyl acetate and stirred for 30 min. Then the mixture was filtered through Celite and the filtrate was evaporated. The crude residue was purified by Combi-Flash to give **PKS8178** (96 mg, 81%) as a white solid. ^{1}H NMR (500 MHz, $CDCl_3$) (rotamers) δ 8.56 – 8.47

(m, 1H), 7.80 (d, *J* = 1.6 Hz, 1H), 7.71 (dt, *J* = 7.6, 3.6 Hz, 1H), 7.36 (d, *J* = 7.8 Hz, 0.5 H), 7.31 (dd, *J* = 7.9, 1.7 Hz, 1H), 7.29 – 7.23 (m, 0.5H), 7.23 – 7.18 (m, 1.5H), 7.15 – 7.09 (m, 0.5H), 4.68 – 4.42 (m, 6H), 1.42 (s, 9H).

PKS8179: To a solution of **PKS8178** (96 mg, 211 μmol) in DCM (5 mL) was added manganese dioxide (86 mg, 845 μmol, 85% purity). The mixture was stirred at ambient temperature for 24 h. The reaction was then filtered through Celite and evaporated to give PKS8179 (87 mg, 91%) as a white solid that was used without further purification. ^{1}H NMR (500 MHz, $CDCl_3$) δ 9.91 (s, 1H), 8.59 – 8.48 (m, 1H), 8.29 (d, *J* = 1.7 Hz, 1H), 7.88 – 7.76 (m, 1H), 7.71 (dt, *J* = 7.7, 4.6 Hz, 1H), 7.38 (d, *J* = 8.2 Hz, 1H), 7.24 – 7.19 (m, 1H), 7.19 – 7.13 (m, 1H), 4.73 – 4.47 (m, 4H), 1.44 – 1.39 (m, 9H).

PKS8181: A mixture of **PKS8179** (85 mg, 188 μmol) and triBoc-cyclam (94 mg, 188 μmol) in DCM (4 mL) was stirred at ambient temperature for 2 h and then sodium triacetoxyborohydride (120 mg, 564 μmol) was added. The resulting mixture was stirred at ambient temperature overnight. Excess reagent was quenched with aqueous $NaHCO_3$, the layers were separated and the aqueous layer was extracted with dichloromethane. The combined organic layers were dried over anhydrous sodium sulfate and evaporated, and the crude residue was purified by Combi-Flash (silica gel; ethyl acetate in hexane) to give **PKS8181** (140 mg, 80%) as a colorless gum which turned into a fluffy solid under vacuum. ^{1}H NMR (500 MHz, $CDCl_3$) δ 8.53 (d, *J* = 4.6 Hz, 1H), 7.77 – 7.62 (m, 2H), 7.36 – 7.28 (m, 1H), 7.25 – 7.10 (m, 3H), 4.69 – 4.41 (m, 6H), 3.58 – 3.04 (m, 12H), 2.70 – 2.50 (m, 2H), 2.36 (s, 2H), 1.99 – 1.74 (m, 2H), 1.62 (s, 2H), 1.52 – 1.32 (m, 36H).

PKS8183: Ethynyl(trimethyl)silane (25 mg, 256 μmol) and triethyl amine (1.5 eq.) were added to a suspension of copper iodide (16 mg, 85 μmol) in THF (1 mL) under argon atmosphere and the mixture was stirred at ambient temperature for 1 h. In parallel, **PKS8181** (80 mg, 85 μmol), *trans*-dichlorobis(triphenylphosphine)palladium (II) (30 mg, 43 μmol) and triethylamine (1.5 eq.) in THF (1 mL) were stirred at ambient temperature for 1 h in a microwave vessel flushed with argon to give a yellow suspension. The copper acetylide reagent was added to the microwave vessel to give a clear solution. The mixture was heated at 120 °C under microwave for 1 h. The mixture was cooled, the solvent was evaporated, and the crude residue was purified by Combi-Flash (silica gel; 0 - 10 % gradient of methanol in dichloromethane) to give **PKS8183** (42 mg, 54%) as a white solid. ^{1}H NMR (500 MHz, DMSO-d_6) δ 8.50 (d, *J* = 4.8 Hz, 1H), 7.76 (t, *J* = 7.6 Hz, 1H), 7.36 (s, 1H), 7.33 – 7.13 (m, 4H), 4.68 – 4.33 (m, 4H), 3.46 (s, 2H), 3.38 – 2.97 (m, 12H), 2.60 – 2.51 (m, 2H), 2.38 – 2.17 (m, 2H), 1.92 – 1.74 (m, 2H), 1.67 – 1.50 (m, 2H), 1.45 – 1.20 (m, 36H), 0.09 (s, 9H).

p-RPS-534: **PKS8183** (30 mg, 33 μmol) was dissolved in THF (2 mL) and cooled to 10 °C. Then tetrabutylammonium fluoride (1.0 M in THF, 33 μL, 33 μmol) was added, the solution was warmed to ambient temperature, and stirred for 1 h. The solvent was evaporated, and crude residue was purified by HPLC to give **p-RPS-534** (22.5 mg, 81%) as a white solid. ^{1}H NMR (500 MHz, DMSO-d_6) δ 8.50 (d, *J* = 4.8 Hz, 1H), 7.79 – 7.73 (m, 1H), 7.36 (s, 1H), 7.31 (d, *J* = 8.0 Hz, 1H), 7.26 (dd, *J* = 7.5, 4.9 Hz, 1H), 7.24 – 7.14 (m, 2H), 4.68 – 4.36 (m, 4H), 4.31 (s, 1H), 3.47 (s, 2H), 3.29 – 3.02 (m, 12H), 2.54 – 2.50 (m, 2H), 2.38 – 2.17 (m, 2H), 1.89 – 1.73 (m, 2H), 1.68 – 1.53 (m, 2H), 1.43 – 1.22 (m, 36H).

PKS8233: Copper sulfate pentahydrate (0.5 M, 18 μL) and sodium ascorbate (1.5 M, 12 μL) were mixed in an argon atmosphere and stirred for 20 min. The solution turned black and then brown. In another round bottom flask, **p-RPS-534** (7.4 mg, 9 μmol) and 1-azido-2-fluoro-ethane (1.6 mg, 18 μmol) were dissolved in DMF (1 mL). To this mixture was added the Cu(I) reagent, resulting in a dull green solution. The reaction was stirred at ambient temperature overnight, diluted with dichloromethane and passed through a basic alumina plug. The filtrate was evaporated, and the crude residue was purified by HPLC to give **PKS8233** (5.8 mg, 71%) as a white solid. ^{1}H NMR (500 MHz, DMSO-d$_6$) δ 8.45 (d, *J* = 4.8 Hz, 1H), 8.41 – 8.30 (m, 1H), 7.76 – 7.68 (m, 1H), 7.59 – 7.47 (m, 1H), 7.31 (d, *J* = 8.0 Hz, 1H), 7.28 – 7.20 (m, 2H), 7.17 (d, *J* = 7.8 Hz, 1H), 4.94 – 4.86 (m, 1H), 4.86 – 4.64 (m, 5H), 4.45 – 4.30 (m, 2H), 3.54 (s, 2H), 3.54 – 3.07 (m, 12H), 2.62 – 2.52 (m, 2H), 2.42 – 2.27 (m, 2H), 1.89 – 1.72 (m, 2H), 1.71 – 1.56 (m, 2H), 1.47 – 1.13 (m, 36H).

RPS-534: **PKS8233** (5.8 mg, 6 μmol) was dissolved in DCM (0.5 mL) and the solution was cooled to 0 °C. Trifluoroacetic acid (0.5 mL) was added to the solution and the mixture was allowed to warm to ambient temperature slowly. After completion of reaction, excess solvent and TFA were evaporated and the crude residue was purified by HPLC to give **RPS-534** (3.2 mg, 42%) as a colorless gum. ^{1}H NMR (500 MHz, DMSO-d$_6$) δ 9.67 (br, 2H), 8.72 (s, 1H), 8.59 (d, *J* = 4.8 Hz, 1H), 7.87 (t, *J* = 7.7 Hz, 1H), 7.67 (d, *J* = 7.8 Hz, 1H), 7.60 (s, 1H), 7.49 (d, *J* = 7.8 Hz, 1H), 7.42 (dd, *J* = 7.6, 5.0 Hz, 1H), 7.37 (d, *J* = 7.9 Hz, 1H), 5.00 – 4.94 (m, 1H), 4.90 – 4.84 (m, 2H), 4.84 – 4.78 (m, 1H), 4.47 (s, 2H), 4.41 (s, 2H), 3.77 (s, 2H), 3.20 – 3.07 (m, 6H), 3.05 (t, *J* = 5.3 Hz, 2H), 2.85 – 2.75 (m, 2H), 2.75 – 2.68 (m, 2H), 2.68 – 2.62 (m, 2H), 2.62 – 2.54 (m, 2H), 2.04 – 1.91 (m, 2H), 1.81 – 1.65 (m, 2H). ^{13}C NMR (126 MHz, DMSO-d$_6$) δ 151.8, 149.1, 145.9, 137.4, 137.2, 132.7, 130.6, 130.5, 130.0, 128.0, 124.3, 123.7, 123.3, 81.8 (d, *J* = 168.2 Hz), 53.7, 50.6 (d, *J* = 19.1 Hz), 49.8, 49.6,49.4, 48.8, 48.4, 47.7, 45.1, 44.2, 43.9, 43.7, 24.8, 22.0. ^{19}F NMR (471 MHz, DMSO-d6) δ -224.8 (tt, *J* = 46.8, 27.8 Hz).

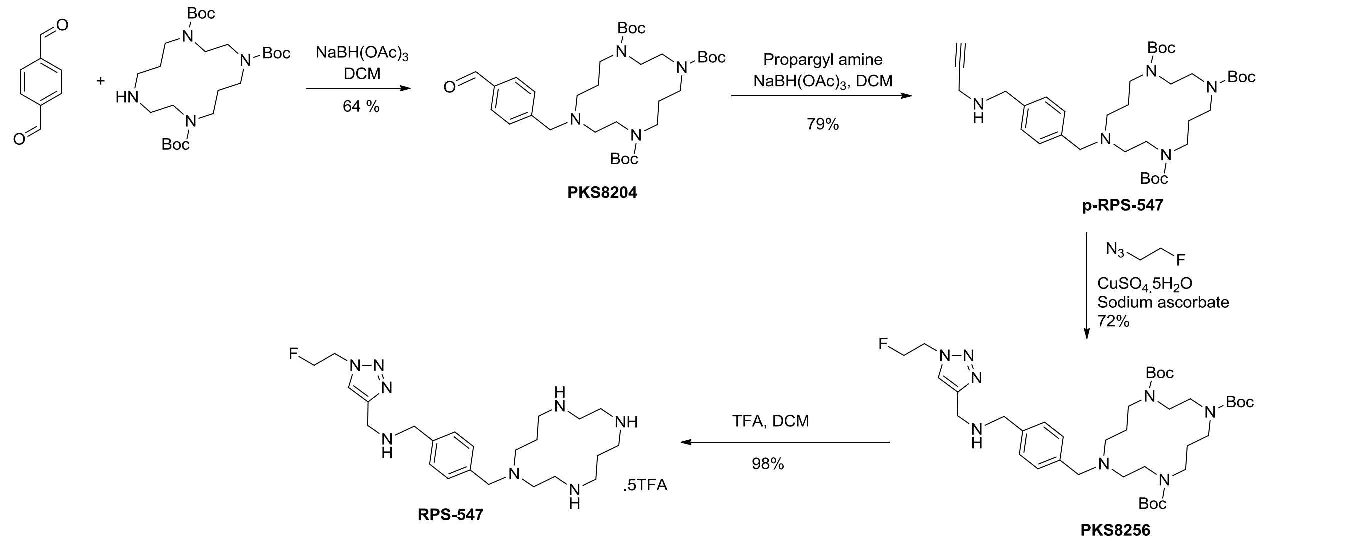

Figure S4. Synthesis of p-RPS-547 and RPS-547.

PKS8204: A mixture of terephthalaldehyde (134 mg, 1.0 mmol) and triBoc-cyclam (250 mg, 500 μmol) in DCM (10 mL) was stirred at ambient temperature for 2 h and then sodium triacetoxyborohydride (318 mg, 1.5 mmol) was added. The mixture was stirred overnight at ambient temperature. The reaction was quenched with aqueous $NaHCO_3$, the layers were separated and the aqueous layer was extracted with dichloromethane. The combined organic layers were dried over anhydrous sodium sulfate and evaporated. The crude residue was purified by Combi-Flash (silica gel; ethyl acetate in hexanes) to give **PKS8204** (198 mg, 64%) as a colorless gum, which turned into a fluffy solid under vacuum. ^{1}H NMR (500 MHz, $CDCl_3$) δ 9.98 (s, 1H), 7.81 (d, *J* = 7.6 Hz, 2H), 7.44 (d, *J* = 7.6 Hz, 2H), 3.60 (s, 2H), 3.51 – 3.16 (m, 12H), 2.73 – 2.53 (m, 2H), 2.50 – 2.30 (m, 2H), 1.96 – 1.78 (m, 2H), 1.76 – 1.63 (m, 2H), 1.52 – 1.17 (m, 27H).

p-RPS-547: A mixture of **PKS8204** (90 mg, 145 μmol), propargyl amine (12 μL, 189 μmol) and acetic acid (4.2 μL, 73 μmol,) in DCM (4 mL) was stirred at ambient temperature for 2 h, then sodium triacetoxyborohydride (37 mg, 175 μmol) was added. The mixture was stirred overnight at ambient temperature, but the reaction did not go to completion. Therefore additional propargyl amine (12 μL) and sodium triacetoxyborohydride (37 mg) were added and the mixture was stirred for an additional 24 h. The reaction was quenched with aqueous $NaHCO_3$, the layers were separated and the aqueous layer was extracted with dichloromethane. The combined organic layers were dried over anhydrous sodium sulfate and evaporated. The crude residue was purified by Combi-Flash (silica gel; ethyl acetate in hexane) and **p-RPS-547** (76 mg, 79%) was isolated as a colorless gum that turned into a fluffy solid under vacuum. ^{1}H NMR (500 MHz, DMSO-d_6) δ 7.27 – 7.15 (m, 4H), 3.70 (s, 2H), 3.47 (s, 2H), 3.30 – 3.09 (m, 14H), 3.06 (t, *J* = 2.4 Hz, 1H), 2.52 (s, 1H), 2.45 – 2.18 (m, 3H), 1.90 – 1.71 (m, 2H), 1.68 – 1.50 (m, 2H), 1.46 – 1.21 (m, 27H).

PKS8256: Copper sulfate pentahydrate (0.5 M, 91 μL) and sodium ascorbate (1.5 M, 91 μL) were mixed in an argon atmosphere and stirred for 20 min to afford a brown solution. In another round bottom flask **p-RPS-547** (15 mg, 23 μmol) and 1-azido-2-fluoro-ethane (0.33 M, 69 μL) were dissolved in DMF (500 μL). The Cu(I) reagent was added to the reaction mixture, resulting in a dull green color. The mixture was stirred at ambient temperature overnight. It was then diluted with dichloromethane and passed through a short plug of basic alumina. The filtrate was evaporated, and the crude residue was purified by HPLC to give **PKS8256** (12 mg, 72%). ^{1}H NMR (500 MHz, DMSO-d_6) δ 8.26 (br, 1H), 7.98 (s, 1H), 7.26 (d, *J* = 7.8 Hz, 2H), 7.21 (d, *J* = 7.8 Hz, 2H), 4.88 – 4.83 (m, 1H), 4.78 – 4.74 (m, 1H), 4.74 – 4.69 (m, 1H), 4.68 – 4.63 (m, 1H), 3.72 (s, 2H), 3.68 (s, 2H), 3.48 (s, 2H), 3.54 – 3.44 (m, 2H), 3.35 – 3.20 (m, 8H), 3.20 – 3.08 (m, 2H), 2.56 – 2.51 (m, 2H), 2.38 – 2.20 (m, 2H), 1.80 (s, 2H), 1.60 (s, 2H), 1.44 – 1.22 (m, 27H).

RPS-547: **PKS8256** (12 mg, 16 μmol) was dissolved in dichloromethane (0.5 mL) and the solution was cooled to 0 °C. Trifluoroacetic acid (0.5 mL) was added dropwise with constant stirring. The reaction mixture was slowly warmed to ambient temperature. After completion of the reaction, the solvents were evaporated and the crude residue was dried under vacuum and triturated with diethyl ether to give a white solid. The solid was purified by HPLC to give **RPS-547** (16 mg, 98%) as a colorless gum. ^{1}H NMR (500 MHz, CD_3OD) δ 8.17 (s, 1H), 7.56 (d, *J* = 7.6 Hz, 2H), 7.40 (d, *J* = 7.6 Hz, 2H), 4.89 – 4.86 (m, 1H), 4.82 – 4.79 (m, 1H), 4.78 – 4.73 (m, 2H), 4.40 (s, 2H), 4.31 (s, 2H), 3.84 (s, 2H), 3.34 – 3.29 (m, 2H), 3.26 (t, *J* =

5.3 Hz, 2H), 3.22 – 3.14 (m, 4H), 3.03 – 2.91 (m, 4H), 2.80 – 2.69 (m, 4H), 2.14 – 2.03 (m, 2H), 1.96 – 1.85 (m, 2H). ^{13}C NMR (126 MHz, CD_3OD) δ 139.6, 137.8, 132.2, 132.1, 131.6, 127.2, 82.8 (d, *J* = 170.7 Hz), 55.9, 52.2, 52.0 (d, *J* = 20.0 Hz), 51.7, 51.3, 50.4, 50.3, 47.3, 46.9, 45.9, 45.8, 42.4, 26.0, 23.5. ^{19}F NMR (471 MHz, CD_3OD) δ -224.25 (tt, *J* = 46.6, 27.6 Hz).

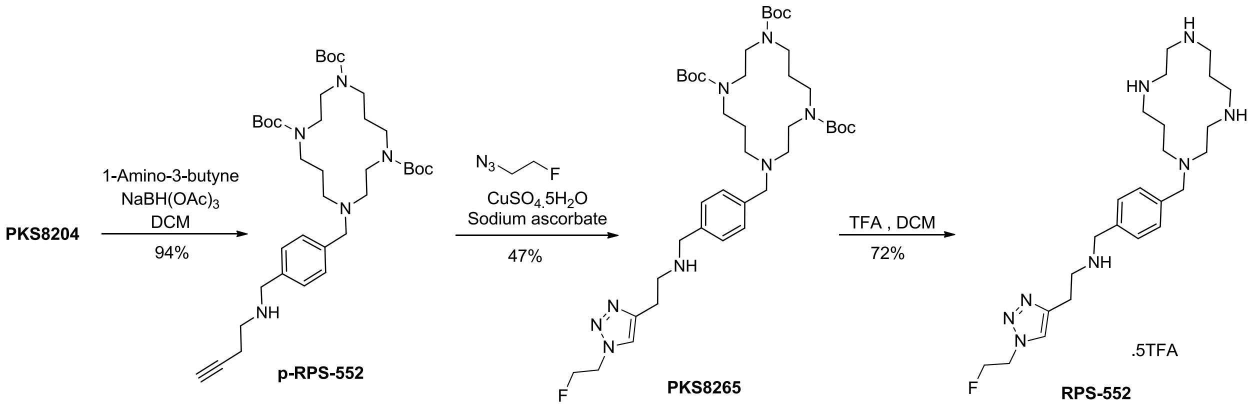

Figure S5. Synthesis of p-RPS-552 and RPS-552.

p-RPS-552: A mixture of **PKS8204** (63 mg, 101 μmol) 1-amino-3-butyne (17 μL, 202 μmol) and acetic acid (3.0 μL, 51 μmol,) in dichloromethane (4 mL) was stirred at ambient temperature for 2 h and then sodium triacetoxyborohydride (43 mg, 202 μmol) was added. The mixture was stirred at ambient temperature overnight. The reaction was quenched with aqueous $NaHCO_3$, the layers were separated and the aqueous layer was extracted with dichloromethane. The combined organic layers were dried over anhydrous sodium sulfate and evaporated, and the crude residue was purified by Combi-Flash (silica gel; ethyl acetate in hexane) to give p-RPS-552 (64 mg, 94%) as a colorless gum which turned into a fluffy solid under vacuum. ^{1}H NMR (500 MHz, DMSO-d_6) δ 7.25 (d, *J* = 7.7 Hz, 2H), 7.21 (d, *J* = 7.7 Hz, 2H), 3.71 (s, 2H), 3.47 (s, 2H), 3.30 – 3.20 (m, 10H), 3.20 – 3.07 (m, 2H), 2.78 (t, *J* = 2.7 Hz, 1H), 2.65 (t, *J* = 7.0 Hz, 2H), 2.56 – 2.51 (m, 2H), 2.39 – 2.16 (m, 4H), 1.87 – 1.71 (m, 2H), 1.67 – 1.48 (m, 2H), 1.49 – 1.12 (m, 27H).

PKS8265: Copper sulfate pentahydrate (0.5 M, 200 μL) and sodium ascorbate (1.5 M, 200 μL) were mixed under an argon atmosphere and stirred for 20 min. The solution turned black and then brown. In another round bottom flask **p-RPS-552** (34 mg, 50 μmol) and 1-azido-2-fluoro-ethane (0.33 M, 303 μL) were dissolved in DMF (500 μL). The Cu(I) reagent was added to the mixture, giving a dull green color. The mixture was stirred at ambient temperature overnight. It was then diluted with dichloromethane and passed through a short plug of basic alumina. The filtrate was evaporated, and the crude residue was purified by HPLC to give **PKS8265** (18 mg, 47%) as a colorless liquid. ^{1}H NMR (500 MHz, DMSO-d_6) δ 8.01 (s, 1H), 7.42 (d, *J* = 7.7 Hz, 2H), 7.33 (d, *J* = 7.7 Hz, 2H), 4.89 – 4.82 (m, 1H), 4.79 – 4.73 (m, 1H), 4.73 – 4.68 (m, 1H), 4.68 – 4.62 (m, 1H), 4.14 (s, 2H), 3.53 (s, 2H), 3.43 – 3.06 (m, 14H), 3.01 (t, *J* = 7.9 Hz, 2H), 2.60 – 2.52 (m, 2H), 2.39 – 2.19 (m, 2H), 1.90 – 1.72 (m, 2H), 1.68 – 1.53 (m, 2H), 1.48 – 1.19 (m, 27H).

RPS-552: **PKS8265** (17 mg, 22 μmol) was dissolved in dichloromethane (0.5 mL) and the solution was cooled to 0 °C. Trifluoroacetic acid (0.5 mL) was added to the solution and the mixture was slowly

warmed to ambient temperature. After completion of reaction, the solvents were evaporated and the crude residue was purified by HPLC to give **RPS-552** (16 mg, 72%) as a TFA salt and a colorless gum. ^{1}H NMR (500 MHz, CD_3OD) δ 7.92 (s, 1H), 7.56 (d, *J* = 7.7 Hz, 2H), 7.40 (d, *J* = 7.7 Hz, 2H), 4.86 – 4.82 (m, 1H), 4.78 – 4.72 (m, 2H), 4.72 – 4.67 (m, 1H), 4.30 (s, 2H), 3.84 (s, 2H), 3.40 (t, *J* = 7.5 Hz, 2H), 3.34 – 3.24 (m, 4H), 3.22 – 3.17 (m, 4H), 3.15 (t, *J* = 7.5 Hz, 2H), 3.05 – 2.92 (m, 4H), 2.79 – 2.69 (m, 4H), 2.14 – 2.03 (m, 2H), 1.95 – 1.87 (m, 2H). ^{13}C NMR (126 MHz, CD_3OD) δ 144.1, 137.8, 132.3, 132.1, 131.5, 124.8, 82.8 (d, *J* = 170.5 Hz), 55.9, 52.1, 51.9 (d, *J* = 20.1 Hz), 51.8, 51.6, 50.1, 50.0, 47.5, 47.2, 46.8, 45.9, 45.6, 25.9, 23.5, 23.0. ^{19}F NMR (471 MHz, CD_3OD) δ -223.8 (tt, *J* = 47.2, 27.3 Hz).

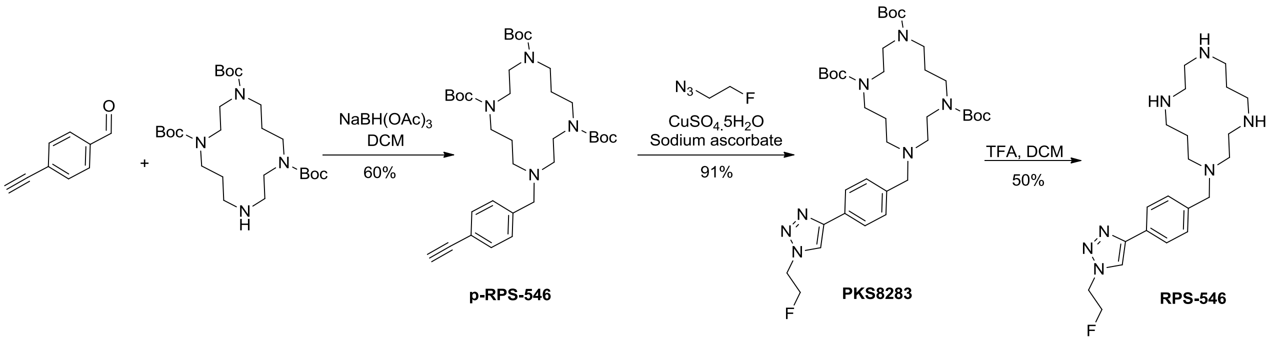

Figure S6. Synthesis of p-RPS-546 and RPS-546.

p-RPS-546: A mixture of 4-ethynylbenzaldehyde (65 mg, 500 μmol) and triBoc-cyclam (250 mg, 500 μmol) in dichloromethane (4 mL) was stirred at ambient temperature for 2 h, and then sodium triacetoxyborohydride (318 mg, 1.5 mmol) was added. The mixture was stirred at ambient temperature overnight. The reaction was quenched with aqueous $NaHCO_3$, the layers were separated and the aqueous layer was extracted with dichloromethane. The combined organic layers were dried over anhydrous sodium sulfate and evaporated, and the crude residue was purified by Combi-Flash (silica gel; ethyl acetate in hexanes) to give **p-RPS-546** (185 mg 60%) as a white solid. ^{1}H NMR (500 MHz, DMSO-d_6) δ 7.38 (d, *J* = 7.7 Hz, 2H), 7.28 (d, *J* = 7.7 Hz, 2H), 4.12 (s, 1H), 3.49 (s, 2H), 3.34 – 3.07 (m, 12H), 2.58 – 2.43 (m, 2H), 2.40 – 2.20 (m, 2H), 1.88 – 1.72 (m, 2H), 1.71 – 1.53 (m, 2H), 1.46 – 1.20 (m, 27H).

PKS8283: Copper sulfate pentahydrate (0.5 M, 163 μL) and sodium ascorbate (1.5 M, 54 μL) were mixed under an argon atmosphere and stirred for 20 min. The solution turned black and then brown. In another round bottom flask **p-RPS-546** (50 mg, 81 μmol) and 1-azido-2-fluoro-ethane (0.33 M, 246 μL) were dissolved in DMF (500 μL). The Cu(I) reagent was added to the mixture. The solution turned dull green. Mixture was stirred at ambient temperature overnight. The mixture was diluted with dichloromethane and passed through a basic alumina plug. The filtrate was evaporated, and isolated crude was purified by HPLC to give product (52.0 mg, 91%) as white solid. ^{1}H NMR (500 MHz, $CDCl_3$) δ 7.86 (s, 1H), 7.76 (d, *J* = 7.9 Hz, 2H), 7.32 (d, *J* = 7.9 Hz, 2H), 4.92 – 4.85 (m, 1H), 4.82 – 4.73 (m, 2H), 4.72 – 4.67 (m, 1H), 3.55 (s, 2H), 3.48 – 3.20 (m, 12H), 2.71 – 2.52 (m, 2H), 2.48 – 2.30 (m, 2H), 1.99 – 1.79 (m, 2H), 1.74 – 1.63 (m, 2H), 1.49 – 1.28 (m, 27H).

RPS-546: To a solution of **PKS8283** (50 mg, 71 μmol) in dichloromethane (1 mL), trifluoroacetic acid (1 mL) was added at 0 °C. The mixture was slowly warmed to ambient temperature and stirred at ambient temperature. After completion of reaction, the solvent was evaporated and the crude residue was purified by HPLC to give RPS-546 as a mixed TFA/formate salt (30 mg, 50%). ^{1}H NMR (500 MHz, CD_3OD) δ 8.51 (s, 1H; formate), 8.40 (s, 1H), 7.90 (d, *J* = 7.9 Hz, 2H), 7.41 (d, *J* = 7.9 Hz, 2H), 4.93 – 4.90 (m, 1H), 4.82 (t, *J* = 4.8 Hz, 2H), 4.79 – 4.74 (m, 1H), 3.81 (s, 2H), 3.25 (t, *J* = 5.7 Hz, 2H), 3.23 – 3.18 (m, 2H), 3.17 – 3.10 (m, 4H), 3.02 – 2.93 (m, 4H), 2.84 – 2.74 (m, 4H), 2.11 – 2.01 (m, 2H), 1.92 – 1.84 (m, 2H). ^{13}C NMR (126 MHz, CD_3OD) δ 170.1 (formate), 148.4, 137.1, 131.7, 131.6, 127.1, 123.1, 82.8 (d, *J* = 170.7 Hz), 56.9, 53.2, 52.0 (d, *J* = 20.2 Hz), 51.9, 50.6, 50.4, 47.5, 47.1, 46.0, 45.9, 25.8, 23.7. ^{19}F NMR (471 MHz, CD_3OD) δ -224.3 (tt, *J* = 46.9, 27.1 Hz).

References:

1. Shankar, S.; Vaidyanathan, G.; Affleck, D.; Welsh, P.C.; Zalutsky, M.R. N-Succinimidyl 3-[^{131}I]Iodo-4-phosphonomethylbenzoate ([^{131}I]SIPMB), a Negatively Charged Substituent-Bearing Acylation Agent for the Radioiodination of Peptides and mAbs. *Bioconjugate Chem.* **2003**, *14*, 331-341.

2. Boss, S.D.; Betzel, T.; Müller, C.; et al. Comparative Studies of Three Pairs of α- and γ-Conjugated Folic Acid Derivatives Labeled with Fluorine-18. *Bioconjugate Chem.* **2016**, *27*, 74-86.

Synthesis of Pentixafor

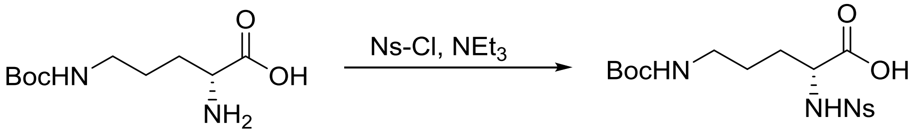

2 **3**

N^{α}-Ns-N^{δ}-Boc-D-Ornithine **(3):** Boc-D-Orn-OH **2** (2333mg, 10 mmol) was dissolved in a mixture of H_2O (10 mL), THF (3.2 mL) and triethylamine (4.17 mL, 30 mmol). The solution was cooled in an ice/water bath, and 2-nitrobenzenesulfonyl chloride (2078 mg, 12.6 mmol) was added in portions. The reaction mixture was stirred at room temperature for 18 h. It was concentrated in vacuo until approximately half of the original volume remained, then it was acidified with concentrated 1M HCl to a pH of 3, and extracted with EtOAc (3 × 150 mL). The organic layers were combined, washed with brine, dried over Na_2SO_4, filtered, and concentrated under reduced pressure to yield **3** as a yellow solid, which was used in the next step without any further purification Yield = 3370 mg, 81%. ^{1}H NMR (500 MHz, Methanol-d4) δ 8.09 (dd, *J* = 6.0, 3.3 Hz, 1H), 7.92 – 7.83 (m, 1H), 7.83 – 7.73 (m, 2H), 3.94 (d, *J* = 3.4 Hz, 1H), 3.02 (t, *J* = 6.8 Hz, 2H), 1.83 (dd, *J* = 10.1, 5.1 Hz, 1H), 1.76 – 1.64 (m, 1H), 1.55 (t, *J* = 7.6 Hz, 2H), 1.42 (s, 9H).^{13}C NMR (126 MHz, CD_3OD) δ 175.3, 158.5, 149.3, 135.10, 134.9, 133.6, 131.5, 126.1, 79.8, 58.2, 40.7, 31.5, 28.7, 26.9. HRMS calc. for $C_{16}H_{23}N_3O_8S$ $[M+Na]^+$: 440.1104 Found: 440.1094.

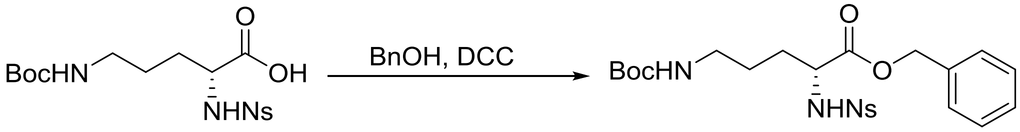

3 **4**

Benzyl (R)-5-((tert-butoxycarbonyl)amino)-2-((2-nitrophenyl)sulfonamido)pentanoate **(4):** Benzyl alcohol (972 mg, 9 mmol) and DMAP (0.0366 g, 0.3 mmol) were added to a solution of **3** (1.25 g, 3 mmol) in CH_2Cl_2 (60 mL) and the mixture was cooled in an ice bath. Dicyclohexylcarbodiimide (772 mg, 3.75 mmol) was added and stirring was continued overnight, allowing the mixture to warm up to rt. The white solid was removed by vacuum filtration. The filtrate was concentrated and purified by Combi-Flash using EtOAc in hexane (25% to 100%) gave **4** as clear viscous liquid. Yield = 1.32 g, 87%. ^{1}H NMR (500 MHz, $CDCl_3$) δ 8.01 (dd, *J* = 7.7, 1.6 Hz, 1H), 7.83 (dd, *J* = 7.9, 1.4 Hz, 1H), 7.64 (dtd, *J* = 24.9, 7.6, 1.5 Hz, 2H), 7.39 – 7.30 (m, 3H), 7.21 – 7.15 (m, 2H), 6.20 (d, *J* = 9.1 Hz, 1H), 4.96 – 4.87 (m, 2H), 4.24 (td, *J* = 8.6, 4.8 Hz, 1H), 3.15 (q, *J* = 6.4 Hz, 2H), 1.93 (dddd, *J* = 14.2, 9.8, 6.7, 4.9 Hz, 1H), 1.81 – 1.70 (m, 1H), 1.61 (tdd, *J* = 9.7, 7.9, 4.8 Hz, 2H), 1.46 (s, 9H).^{13}C NMR (126 MHz, $CDCl_3$) δ 170.8, 156.1, 147.7, 134.8, 134.1, 133.7, 132.9, 130.4, 128.8, 128.7, 128.5, 125.8, 79.4, 67.5, 56.6, 39.7, 30.6, 28.5, 26.0. HRMS calc. for $C_{23}H_{29}N_3O_8S$ $[M+Na]^+$: 530.1573 Found: 530.1559.

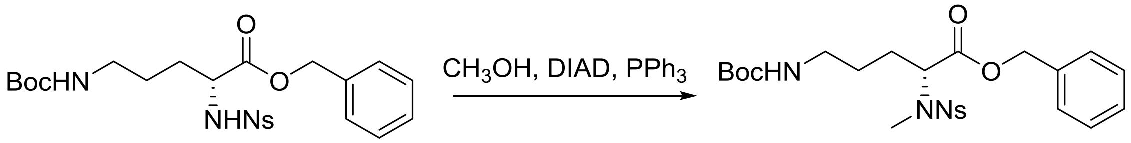

4 **5**

Benzyl (R)-5-((tert-butoxycarbonyl)amino)-2-((N-methyl-2-nitrophenyl)sulfonamido)pentanoate **(5):** To an ice cold solution of solution of **4** (1.04 g, 2 mmol) and triphenylphosphine (786 mg, 3 mmol) in THF (20 mL) was added diisopropyl azodicarboxylate (606 mg or 0.59 mL, 3 mmol) followed by MeOH (320 mg or 0.4 mL, 10 mmol) and the resulting reaction mixture was stirred overnight at room temperature. The crude compound was purified by using Combi-Flash using EtOAc in hexane (10% to 50%) gave **5** as an amber colored liquid. Yield = 946 mg, 71%. ^{1}H NMR (500 MHz, CD_3OD) δ 8.03 (d, *J* = 7.8 Hz, 1H), 7.73 – 7.60 (m, 3H), 7.38 – 7.27 (m, 3H), 7.25 – 7.16 (m, 2H), 5.03 (d, *J* = 12.1 Hz, 1H), 4.94 (d, *J* = 12.1 Hz, 1H), 4.70 (dd, *J* = 10.9, 4.8 Hz, 1H), 3.09 (q, *J* = 7.0 Hz, 2H), 2.95 (s, 3H), 2.03 (td, *J* = 9.5, 5.1 Hz, 1H), 1.85 – 1.70 (m, 1H), 1.63 – 1.47 (m, 2H), 1.44 (s, 9H). ^{13}C NMR (126 MHz, CD_3OD) δ 170.2, 157.1, 147.9, 135.3, 133.7, 131.8, 131.3, 130.5, 128.1, 128.0, 123.8, 78.5, 66.7, 59.3, 39.0, 29.4, 27.3, 26.2, 25.8. HRMS calc. for $C_{24}H_{31}N_3O_8S$ $[M+Na]^+$: 544.1730 Found: 544.1719.

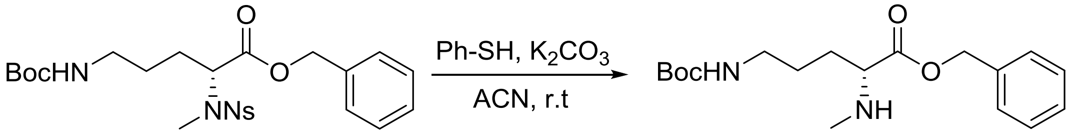

5 **6**

Benzyl (R)-5-((tert-butoxycarbonyl)amino)-2-(methylamino)pentanoate **(6):** To a stirred suspension of **5** (1.042 g, 2 mmol) and K_2CO_3 (690 mg, 5 mmol) in acetonitrile (20 mL) was added thiophenol (2.2 g, 20 mmol) at room temperature and stirred overnight. The reaction was filtered and the filter cake was washed with acetonitrile (5 mL) and the combined filtrates was evaporated to dryness. The viscous liquid thus obtained was dissolved in DCM and washed with water, dried over Na_2SO_4, filtered and dried in vacuo. Compound **6** was isolated as clear liquid and used without further purification. Yield = 550 mg, 82%. ^{1}H NMR (500 MHz, CD_3OD) δ 7.51 – 7.30 (m, 5H), 5.33 (m, 2H), 4.12 (dd, *J* = 7.1, 5.1 Hz, 1H), 3.05 (q, *J* = 6.5 Hz, 2H), 2.74 (s, 3H), 1.95 (m, 2H), 1.57 (td, *J* = 12.2, 5.9 Hz, 1H), 1.44 (s, 9H), 1.43 – 1.25 (m, 1H). ^{13}C NMR (126 MHz, CD_3OD) δ 169.7, 158.6, 136.2, 129.9, 129.9, 129.8, 129.8, 80.1, 69.4, 61.6, 40.30, 32.1, 28.7, 27.4, 26.2. HRMS calc. for $C_{18}H_{28}N_2O_4$ $[M+H]^+$: 337.2127 Found: 337.2116.

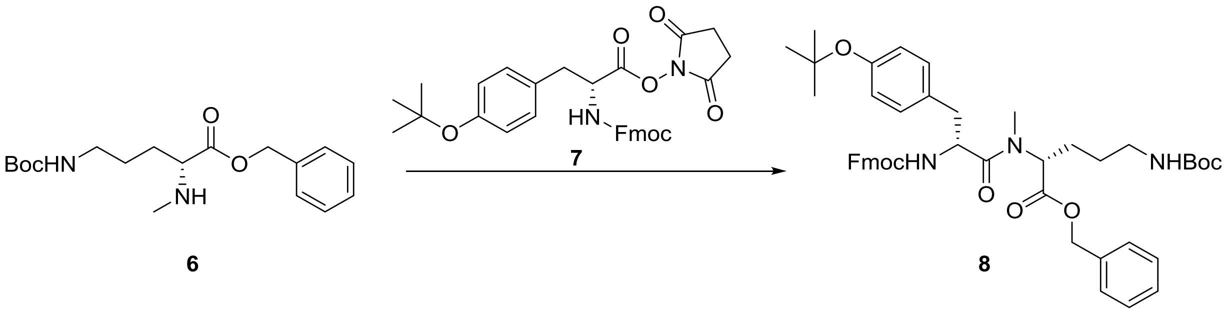

Benzyl (R)-2-((R)-2-((((9H-fluoren-9-yl)methoxy)carbonyl)amino)-3-(4-(tert-butoxy)phenyl)-N-methylpropanamido)-5-((tert-butoxycarbonyl)amino)pentanoate **(8):** To a solution of **6** (336 mg, 1 mmol) in DMF (5 mL) was added dropwise NHS ester **7** (552 mg, 1.2 mmol) in DMF (5 mL) at room temperature and the resulting mixture was stirred for 6 h under N_2. DMF was removed under reduced pressure and the resulting residue was dissolved in EtOAc, transferred to a separating funnel and washed successively with water and brine. The organic layer was dried over Na_2SO_4, filtered and concentrated under reduced pressure. The crude compound was purified by Combi-Flash using EtOAc in hexane (25% to 100%). Yield = 590 mg, 76%. ^{1}H NMR (500 MHz, $CDCl_3$) δ 7.76 (d, *J* = 7.6 Hz, 2H), 7.56 (dd, *J* = 7.8, 3.2 Hz, 2H), 7.40 (t, *J* = 7.4 Hz, 2H), 7.32 (qt, *J* = 7.9, 4.2 Hz, 7H), 7.06 (d, *J* = 8.0 Hz, 2H), 6.86 (d, *J* = 7.9 Hz, 2H), 5.78 (s, 1H), 5.21 – 4.95 (m, 3H), 4.92 – 4.81 (m, 1H), 4.35 (dd, *J* = 10.5, 7.3 Hz, 1H), 4.24 (dd, *J* = 10.5, 7.3 Hz, 1H), 4.16 (t, *J* = 7.4 Hz, 1H), 3.15 – 2.87 (m, 4H), 2.84 (s, 3H), 2.00 (d, *J* = 9.1 Hz, 1H), 1.70 (dtd, *J* = 14.6, 9.9, 4.9 Hz, 1H), 1.43 (m, 10H), 1.30 (m, 10H). ^{13}C NMR (126 MHz, $CDCl_3$) δ ^{13}C NMR (126 MHz, $CDCl_3$) δ 172.9, 170.3, 156.0, 154.4, 143.9, 143.8, 141.4, 141.4, 135.4, 130.7, 130.2, 130.0, 128.8, 128.6, 128.6, 128.4, 128.1, 127.8, 127.8, 127.2, 125.3, 125.3, 125.2, 124.4, 120.1, 79.6, 78.7, 67.3, 67.2, 57.4, 52.5, 47.1, 40.0, 38.1, 28.9, 28.4, 26.6, 25.5. HRMS calc. for $C_{46}H_{55}N_3O_8$ $[M+H]^+$: 778.4067 Found: 778.4058.

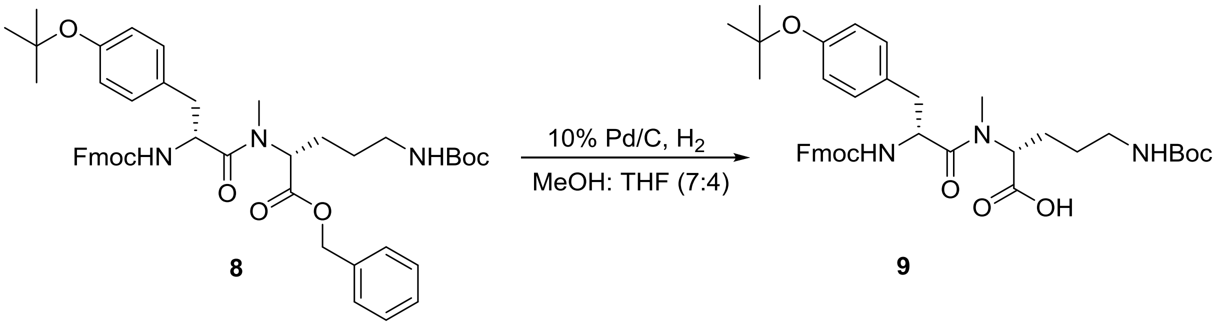

(R)-2-((R)-2-((((9H-fluoren-9-yl)methoxy)carbonyl)amino)-3-(4-(tert-butoxy)phenyl)-N-methylpropanamido)-5-((tert-butoxycarbonyl)amino)pentanoic acid **(9):** Compound **8** (385 mg, 0.5 mmol) was dissolved in a mixture of MeOH and THF (10 mL, 7:4). Then 10% Pd/C was added and the suspension was stirred under H_2 balloon pressure for 3 h. The reaction mixture was filtered through Celite and the filter cake was washed with methanol (3 x 5 mL). The filtrate was evaporated to afford compound **9** as white solid, which was used without any further purification. Yield = 309 mg, 90%. ^{1}H

NMR (500 MHz, CD_3OD) δ 7.79 (d, *J* = 7.6 Hz, 2H), 7.63 (d, *J* = 7.7 Hz, 2H), 7.40 (t, *J* = 7.5 Hz, 2H), 7.31 (td, *J* = 7.4, 4.5 Hz, 2H), 7.19 (d, *J* = 8.0 Hz, 2H), 6.87 (d, *J* = 8.3 Hz, 2H), 4.79 (d, *J* = 8.0 Hz, 2H), 4.32 – 4.05 (m, 3H), 3.03 (dt, *J* = 18.5, 7.3 Hz, 3H), 2.91 (s, 3H), 2.86 (dd, *J* = 9.1, 4.8 Hz, 1H), 2.05 – 1.67 (m, 1H), 1.38 (s, 9H), 1.33 (m, 3H), 1.25 (s, 9H). ^{13}C NMR (126 MHz, CD_3OD) δ 174.7, 174.1, 173.9, 158.4, 158.0, 155.3, 145.2, 145.1, 142.6, 142.5, 133.4, 131.2, 131.0, 130.8, 128.7, 128.3, 128.3, 128.2, 128.1, 126.4, 126.2, 125.2, 125.2, 121.0, 120.92 79.8, 79.5, 68.3, 68.0, 57.8, 54.8, 54.3, 53.9, 40.7, 40.6, 38.2, 33.0, 32.0, 30.7, 30.6, 30.4, 29.2, 29.1, 29.1, 28.8, 28.7, 27.4, 26.4. HRMS calc. for $C_{39}H_{49}N_3O_8$ $[M+Na]^+$: 710.3417 Found: 710.3392.

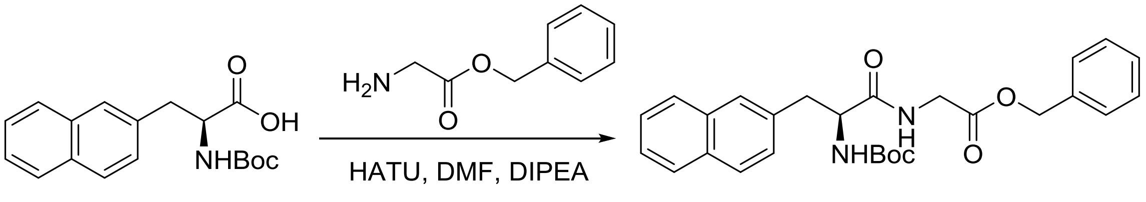

10 **11**

Benzyl (S)-(2-((tert-butoxycarbonyl)amino)-3-(naphthalen-2-yl)propanoyl)glycinate **(11):** To a stirred mixture of **10** (945 mg, 3 mmol), benzyl glycinate (594 mg, 3.6 mmol) and HATU (1.36 g, 3.6 mmol) in DMF (15 mL) was added DIPEA (1.26 mL, 7.2 mmol) and the reaction was stirred at room temperature overnight under N_2. The solvent was removed under reduce pressure and the resulting residue was dissolved in EtOAc. The organic layers was washed with brine, dried over Na_2SO_4 and filtered. The filtrate was concentrated and purified by Combi-Flash using EtOAc in hexane (10% to 100%). Yield = 360 mg, 78%. ^1H NMR (500 MHz, $CDCl_3$) δ 7.86 – 7.76 (m, 3H), 7.68 (d, *J* = 2.0 Hz, 1H), 7.52 – 7.42 (m, 2H), 7.42 – 7.31 (m, 6H), 6.48 (t, *J* = 5.3 Hz, 1H), 5.15 (d, *J* = 12.1 Hz, 1H), 5.12 (d, *J* = 12.3 Hz, 1H), 5.06 (s, 1H), 4.59 – 4.48 (m, 1H), 4.09 (dd, *J* = 18.3, 5.4 Hz, 1H), 3.99 (dd, *J* = 18.3, 5.0 Hz, 1H), 3.27 (qd, *J* = 14.2, 6.4 Hz, 2H), 1.39 (s, 9H). ^{13}C NMR (126 MHz, $CDCl_3$) δ 171.6, 169.3, 135.2, 134.2, 133.6, 132.6, 128.7, 128.6, 128.5, 128.5, 128.1, 127.7, 127.7, 127.4, 126.2, 125.8, 80.5, 77.4, 77.1, 76.9, 67.3, 55.7, 41.4, 38.4, 28.3. HRMS calc. for $C_{27}H_{30}N_2O_5H$ $[M+Na]^+$: 485.2025 Found: 485.2041.

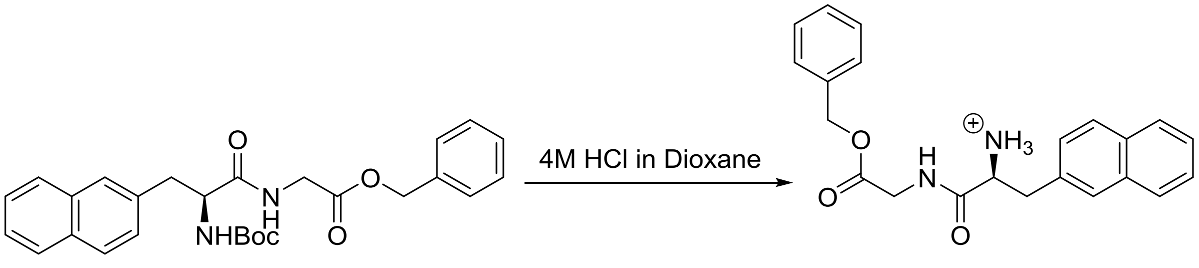

11 **12**

Benzyl (S)-(2-amino-3-(naphthalen-2-yl)propanoyl)glycinate hydrochloride **(12):** To a solution of compound **11** (924 mg, 2 mmol) in dioxane (10 mL) was added 4M HCl in dioxane (10 mL) and the reaction was stirred at room temperature for 3 h. A white solid was formed during the reaction, and the

solid was isolated by filtration. The solid was washed with diethyl ether and used without any further purification. Yield = 732 mg, 92%. ^{1}H NMR (500 MHz, DMSO-d_6) δ 9.14 (t, *J* = 5.8 Hz, 1H), 8.28 (s, 3H), 7.95 – 7.87 (m, 2H), 7.87 – 7.83 (m, 1H), 7.81 (s, 1H), 7.56 – 7.49 (m, 2H), 7.48 (dd, *J* = 8.4, 1.7 Hz, 1H), 7.43 – 7.33 (m, 5H), 5.18 (s, 2H), 4.23 (t, *J* = 6.8 Hz, 1H), 4.17 – 3.99 (m, 2H), 3.32 (dd, *J* = 14.2, 5.2 Hz, 1H), 3.15 (dd, *J* = 14.2, 8.0 Hz, 1H). ^{13}C NMR (126 MHz, DMSO-d_6) δ δ 169.2, 168.7, 135.8, 133.0, 132.4, 132.2, 128.4, 128.3, 128.2, 128.1, 128.0, 127.6, 127.5, 126.1, 125.8, 66.1, 53.3, 40.8, 40.0, 39.8, 39.6, 39.5, 39.3, 39.1, 39.0, 37.1. HRMS calc. for $C_{22}H_{22}N_2O_3$ [M+H]$^+$: 363.1709 Found: 363.1693.

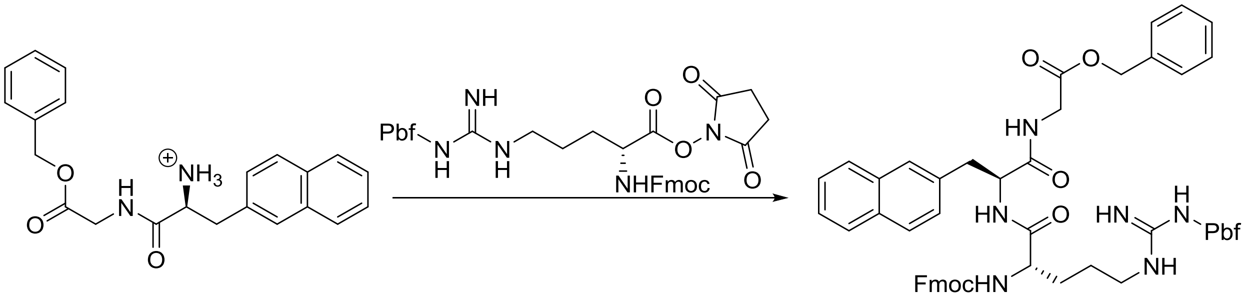

12 **13**

Benzyl ((S)-2-((S)-2-((((9H-fluoren-9-yl)methoxy)carbonyl)amino)-5-(3-((2,2,4,6,7-pentamethyl-2,3-dihydrobenzofuran-5-yl)sulfonyl)guanidino)pentanamido)-3-(naphthalen-2-yl)propanoyl)glycinate **(13):** To a solution of **12** (597 mg, 1.5 mmol) in DMF (10 mL) was added dropwise Fmoc-Arg(Pbf)-NHS ester (1.036 g, 1.6 mmol) in DMF (10 mL) and the reaction was stirred at room temperature 6 h under N_2. The solvent was removed under reduced pressure and the resulting residue was dissolved in EtOAc, transferred in to a separating funnel and washed successively with water and brine. The organic layer was dried over Na_2SO_4, filtered and concentrated under reduced pressure. The crude compound was purified by Combi-Flash using MeOH in DCM (1% to 5%). Yield = 590 mg, 71%. ^{1}H NMR (500 MHz, CD_3OD) δ 7.80 (d, *J* = 7.6 Hz, 2H), 7.73 (d, *J* = 7.9 Hz, 1H), 7.71 – 7.64 (m, 3H), 7.61 (dd, *J* = 7.6, 4.0 Hz, 2H), 7.43 – 7.36 (m, 2H), 7.36 – 7.22 (m, 10H), 5.11 (s, 2H), 4.84 – 4.76 (m, 1H), 4.38 – 4.26 (m, 1H), 4.22 (dd, *J* = 10.6, 6.5 Hz, 1H), 4.12 (t, *J* = 6.9 Hz, 1H), 4.01 (d, *J* = 8.9 Hz, 2H), 3.97 – 3.85 (m, 1H), 3.39 (dd, *J* = 14.1, 5.1 Hz, 1H), 3.08 (dd, *J* = 14.1, 9.7 Hz, 1H), 2.96 (s, 2H), 2.83 (s, 1H), 2.59 (s, 3H), 2.52 (s, 3H), 2.08 (s, 3H), 1.47 (q, *J* = 9.1, 8.6 Hz, 2H), 1.41 (s, 6H), 1.21 (q, *J* = 7.4 Hz, 2H). ^{13}C NMR (126 MHz, CD_3OD) δ 174.4, 173.9, 171.0, 159.8, 158.6, 158.0, 145.3, 145.0, 142.6, 142.5, 139.4, 137.1, 135.9, 134.8, 134.4, 133.8, 133.5, 129.5, 129.3, 129.2, 129.0, 128.8, 128.7, 128.6, 128.5, 128.4, 128.1, 127.0, 126.5, 126.2, 126.0, 120.9, 118.4, 87.6, 67.9, 56.5, 55.3, 43.9, 42.1, 38.5, 29.9, 28.6, 19.6, 18.4, 12.5. HRMS calc. for $C_{56}H_{60}N_6O_9S$ [M+H]$^+$: 993.4221 Found: 993.4213.

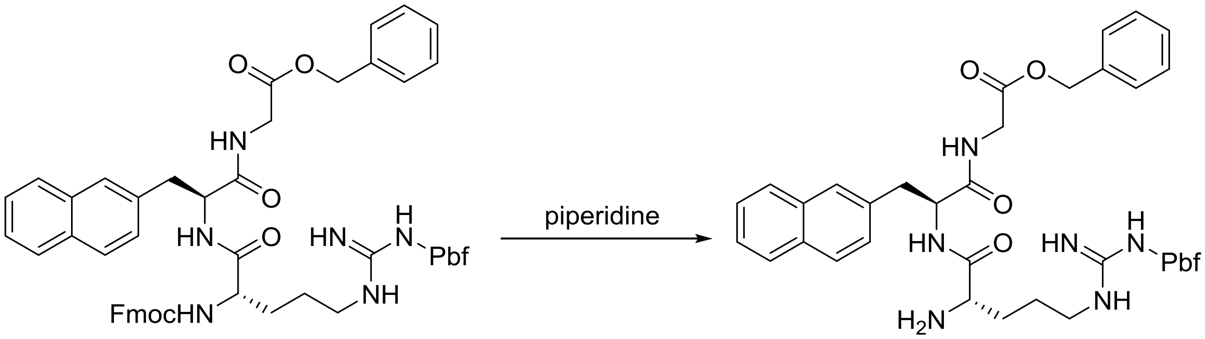

13 **14**

Benzyl ((S)-2-((S)-2-amino-5-(3-((2,2,4,6,7-pentamethyl-2,3-dihydrobenzofuran-5-yl) sulfonyl)guanidino)pentanamido)-3-(naphthalen-2-yl)propanoyl)glycinate **(14):** Compound **13** (993 mg, 1 mmol) was dissolved in dry DCM (20 mL). To the solution was added piperdine (1 mL) and the reaction was stirred at room temperature for 3 h. The solvent was evaporated and the crude product was purified by Combi-Flash using MeOH in DCM (1% to 20%). Yield = 369 mg, 48%. ^{1}H NMR (500 MHz, , CD_3OD) δ 7.78 (t, *J* = 8.3 Hz, 3H), 7.73 (s, 1H), 7.51 – 7.37 (m, 3H), 7.36 – 7.21 (m, 5H), 5.10 (s, 2H), 3.97 (d, *J* = 1.5 Hz, 2H), 3.82 (t, *J* = 6.1 Hz, 1H), 3.39 – 3.31 (m, 1H), 3.10 (m, 3H), 2.97 (s, 2H), 2.55 (s, 3H), 2.49 (s, 3H), 2.06 (s, 3H), 1.85 (q, *J* = 7.6 Hz, 2H), 1.56 (m, 2H), 1.45 (s, 6H). ^{13}C NMR (126 MHz, CD_3OD) δ 173.9, 173.8, 170.8, 170.8, 169.8, 160.0, 158.1, 139.4, 137.1, 135.6, 134.9, 134.0, 133.9, 133.6, 129.5, 129.4, 129.3, 129.1, 128.9, 128.7, 128.6, 128.2, 127.1, 126.7, 126.1, 118.5, 87.7, 67.9, 56.0, 56.02, 53.7, 43.9, 42.2, 42.1, 38.9, 29.6, 28.6, 19.5, 18.3, 12.4. HRMS calc. for $C_{41}H_{50}N_6O_7S$ $[M+H]^+$: 771.3540 Found: 771.3527.

9 + **14**

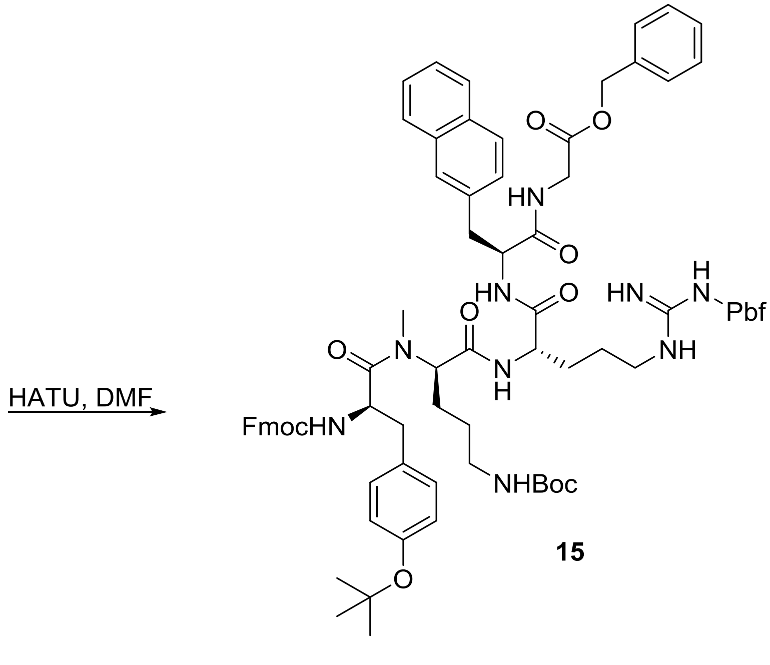

15

Benzyl ((S)-2-((S)-2-((R)-2-((R)-2-((((9H-fluoren-9-yl)methoxy)carbonyl)amino)-3-(4-(tert-butoxy)phenyl)-N-methylpropanamido)-5-((tert-butoxycarbonyl)amino)pentanamido)-5-(3-((2,2,4,6,7-pentamethyl-2,3-

dihydrobenzofuran-5-yl)sulfonyl)guanidino)pentanamido)-3-(naphthalen-2-yl)propanoyl)glycinate **(15):** To a stirred mixture of **9** (343 mg, 0.5 mmol), **14** (385 mg, 0.5 mmol) and HATU (209 mg, 0.55 mmol) in DMF (5 mL) was added DIPEA (0.1 mL, 0.6 mmol) and the mixture was stirred at room temperature overnight under N_2. DMF was removed under reduced pressure and the resulting residue was dissolved in EtOAc, washed with brine, dried over Na_2SO_4, filtered and concentrated under reduced pressure. The crude product was purified by Combi-Flash using MeOH in DCM (1% to 10%). Yield = 374 mg, 52%. ^{1}H NMR (500 MHz, CD_3OD) δ 7.78 – 7.58 (m, 7H), 7.51 (s, 1H), 7.31 (ddt, *J* = 18.9, 14.8, 7.5 Hz, 13H), 7.10 (s, 1H), 6.84 (d, *J* = 7.9 Hz, 2H), 5.11 (q, *J* = 4.1, 3.6 Hz, 2H), 4.75 (dd, *J* = 9.7, 5.3 Hz, 1H), 4.21 (dd, *J* = 30.5, 6.1 Hz, 1H), 4.11 – 3.79 (m, 2H), 3.71 (d, *J* = 6.6 Hz, 3H), 3.34 (s, 2H), 3.20 (d, *J* = 7.5 Hz, 5H), 3.13 (t, *J* = 5.8 Hz, 2H), 2.94 (d, *J* = 25.5 Hz, 8H), 2.84 (s, 4H), 2.70 (s, 1H), 2.56 (d, *J* = 4.2 Hz, 3H), 2.49 (d, *J* = 5.1 Hz, 3H), 2.05 (d, *J* = 7.6 Hz, 3H), 1.77 (q, *J* = 5.9 Hz, 2H), 1.73 – 1.44 (m, 3H), 1.39 (s, 6H), 1.36 (s, 9H), 1.35 (s, 9H). HRMS calc. for $C_{80}H_{97}N_9O_{14}S$ $[M+H]^+$: 1440.6954 Found: 1440.6943.

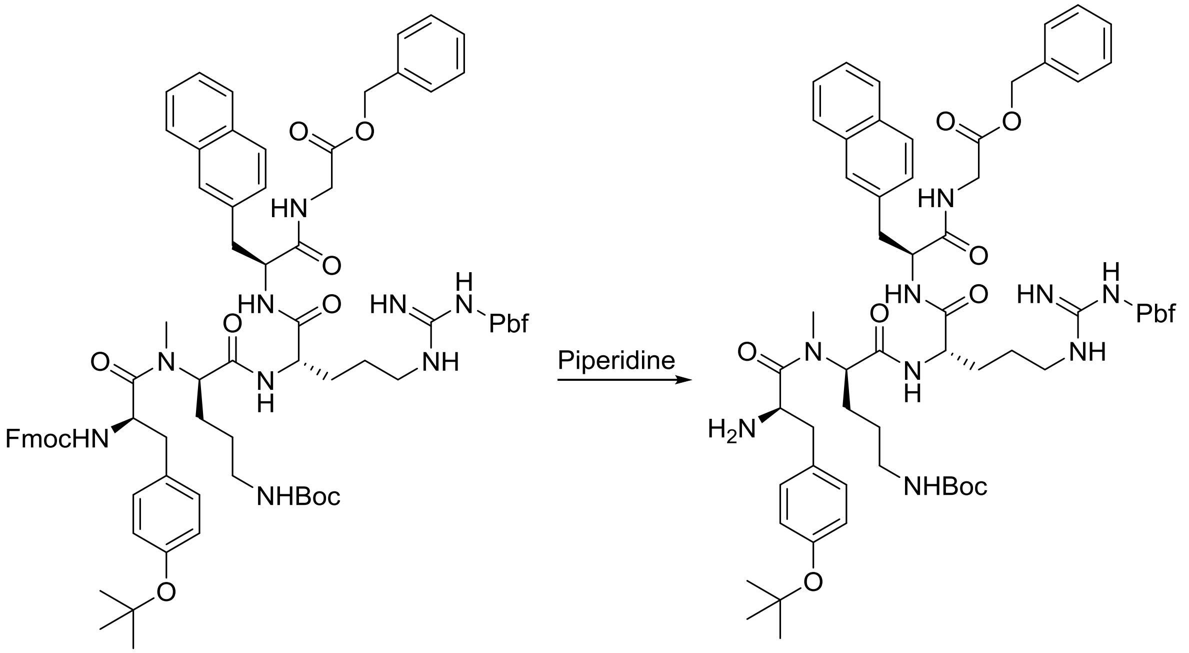

15 **16**

Benzyl((S)-2-((S)-2-((R)-2-((R)-2-amino-3-(4-(tert-butoxy)phenyl)-N-methylpropanamido)-5-((tert-butoxycarbonyl)amino)pentanamido)-5-(3-((2,2,4,6,7-pentamethyl-2,3-dihydrobenzofuran-5-yl)sulfonyl)guanidino)pentanamido)-3-(naphthalen-2-yl)propanoyl)glycinate **(16):** Compound **15** (359 mg, 0.25 mmol) was dissolved in dry DCM (5 mL). To the solution was added piperdine (0.4 mL) and the reaction was stirred at room temperature for 2.5 h. The solvent was evaporated and the crude product was purified by Combi-Flash using MeOH in DCM (1% to 20%). Yield = 121 mg, 40%. ^{1}H NMR (500 MHz, CD_3OD) δ 7.82 – 7.66 (m, 4H), 7.46 – 7.28 (m, 8H), 7.22 – 7.09 (m, 2H), 7.03 – 6.92 (m, 2H), 5.14 (d, *J* = 29.8 Hz, 2H), 4.81 (dd, *J* = 9.0, 5.6 Hz, 1H), 4.42 (ddd, *J* = 56.7, 8.4, 5.5 Hz, 2H), 4.06 – 3.91 (m, 2H), 3.20 – 2.83 (m, 9H), 2.65 (s, 1H), 2.59 (s, 1H), 2.53 (s, 1H), 2.09 (s, 3H), 1.88 – 1.51 (m, 5H), 1.44 (m, 19H), 1.35

(m, 10H). ^{13}C NMR (126 MHz, DMSO) δ 180.7, 180.5, 179.0, 178.7, 178.5, 166.9, 165.5, 165.0, 163.7, 146.7, 145.2, 144.5, 142.3, 141.2, 140.9, 139.6, 138.2, 137.8, 137.6, 137.5, 137.4, 137.4, 137.2, 137.0, 136.8, 136.9, 135.2, 134.8, 133.7, 133.2, 125.7, 95.7, 95.7, 87.4, 87.3, 86.9, 75.4, 75.3, 65.0, 63.0, 61.6, 60.7, 51.9, 50.1, 47.4, 45.0, 39.6, 39.3, 37.9, 37.7, 37.7, 37.6, 35.5, 34.8, 28.4, 27.0, 21.7. HRMS calc. for $C_{65}H_{87}N_9O_{12}S$ $[M+H]^+$:1218.6273 Found: 1218.6267.

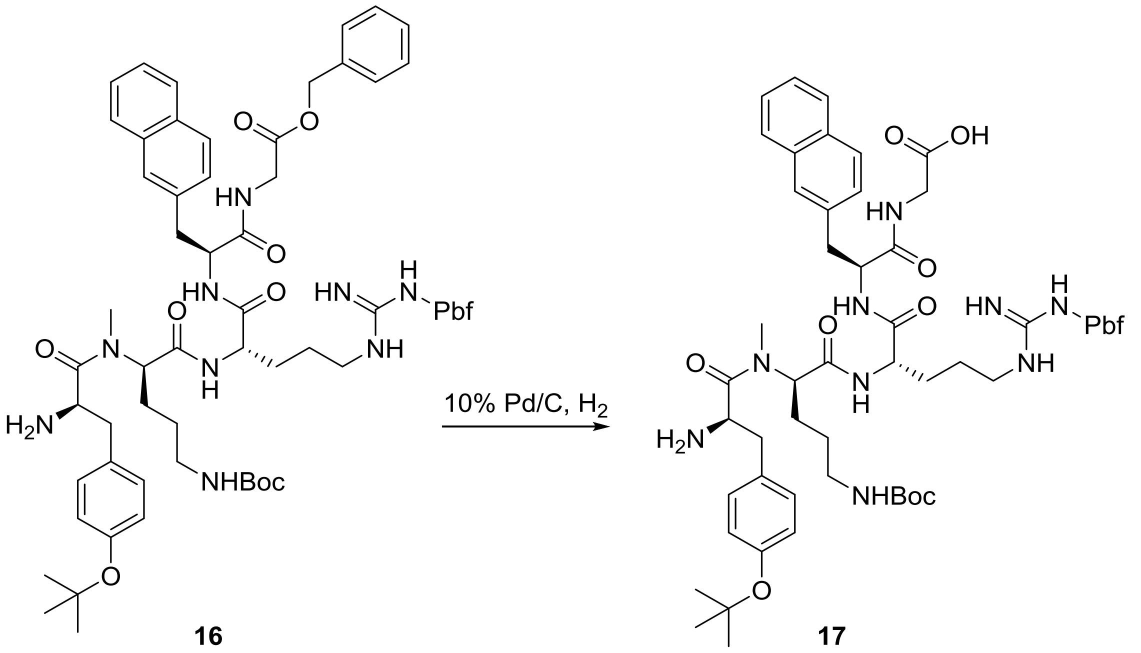

16 17

((S)-2-((S)-2-((R)-2-((R)-2-amino-3-(4-(tert-butoxy)phenyl)-N-methylpropanamido)-5-((tert-butoxycarbonyl)amino)pentanamido)-5-(3-((2,2,4,6,7-pentamethyl-2,3-dihydrobenzofuran-5-yl)sulfonyl)guanidino)pentanamido)-3-(naphthalen-2-yl)propanoyl)glycine **(17):** Compound **16** (244 mg, 0.2 mmol) was dissolved in a mixture of MeOH and THF (10 mL, 7:4). Then 10% Pd/C was added and stirred under H_2 Balloon pressure for 2 h. The reaction was filtered through Celite and the filter cake bed was washed with methanol (3 x 5 mL). The filtrate was evaporated to afford compound **17** as a cream colored solid which was used without further purification. ^1H NMR (500 MHz, CD_3OD) ^1H NMR (500 MHz, Methanol-d_4) δ 7.78 – 7.59 (m, 4H), 7.33 (qd, *J* = 8.3, 3.7 Hz, 3H), 7.10 (dd, *J* = 17.5, 8.1 Hz, 2H), 6.93 (dd, *J* = 8.2, 5.8 Hz, 2H), 4.79 – 4.62 (m, 1H), 4.43 (dd, *J* = 8.2, 5.9 Hz, 1H), 4.29 (dd, *J* = 8.5, 5.4 Hz, 1H), 3.99 – 3.77 (m, 2H), 3.30 (s, 3H), 3.10 – 2.90 (m, 8H), 2.80 (dd, *J* = 14.5, 8.3 Hz, 1H), 2.59 (s, 2H), 2.54 (s, 3H), 2.47 (s, 3H), 2.04 (s, 3H), 1.79 – 1.45 (m, 4H), 1.39 (m, 8H), 1.36 (s, 9H), 1.34 – 1.31 (m, 1H), 1.29 (s, 9H), 1.27 (m, 1H). ^{13}C NMR (126 MHz, CD_3OD) δ 172.2, 171.2, 170.4, 169.3, 157.1, 155.0, 134.4, 133.5, 132.4, 129.8, 128.8, 127.7, 127.6, 127.3, 127.2, 127.1, 125.6, 125.2, 124.3, 124.2, 114.7, 86.3, 78.6, 78.4, 59.3, 57.1, 54.2, 52.8, 52.1, 48.4, 42.5, 40.4, 39.2, 37.8, 35.5, 30.2, 29.0, 27.8, 27.8, 27.4, 27.4, 27.3, 26.0, 24.7, 18.2, 17.0, 11.1. HRMS calc. for $C_{58}H_{81}N_9O_{12}S$ $[M+H]^+$: 1128.5804 Found: 1128.5797.

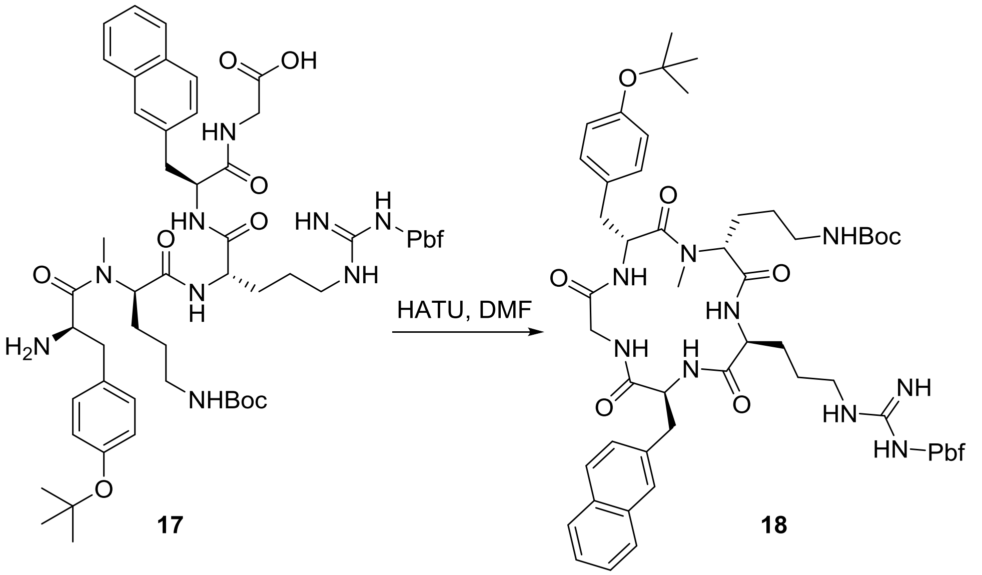

17 18

Cyclo-(D-4-tert-butyl-Tyr-(α-methyl, δ-Boc-D-Orn-R-Nal-Pbf-G)) **(18):** To a solution of HATU (0.070 g, 0.062 mmol) in DMF (3 mL) under inert atmosphere was added a solution of **17** (0.140 g, 0.125 mmol) in DMF (2 mL) followed by slow addition of DIPEA (35 µL) in DMF (3 mL), and the resulting mixture was stirred for 1 h at room temperature. The DMF was removed under reduced pressure and the crude residue was re-dissolved in DMF (3 mL) and loaded onto a preparative HPLC column. Compound **18** was isolated using a linear gradient of 10-90% v/v MeCN/H_2O + 0.05% TFA between 0 to 40 min at a flow rate of 12 mL/min, followed by an isocratic method of 90% MeCN/H_2O + 0.05% TFA from 40 to 50 min and 12 mL/min. The fractions containing the product were collected and lyophilized *in vacuo* to afford **18** as a white powder. ^{1}H NMR (500 MHz, CD_3OD) δ 7.85 – 7.63 (m, 3H), 7.59 (d, *J* = 8.5 Hz, 1H), 7.44 – 7.28 (m, 3H), 7.15 (t, *J* = 8.0 Hz, 2H), 6.92 (dd, *J* = 23.4, 8.2 Hz, 2H), 5.02 (m, 0.36H), 4.95 – 4.87 (m, 1H), 4.77 (dd, *J* = 11.4, 4.2 Hz, 1H), 4.44 – 4.17 (m, 1H), 3.99-3.83 (m, 0.48H), 3.64 – 3.35 (m, 2H), 3.28 – 3.10 (m, 2H), 3.08 – 2.71 (m, 9H), 2.65 (s, 2H), 2.57 (d, *J* = 4.9 Hz, 3H), 2.50 (d, *J* = 9.8 Hz, 3H), 2.07 (d, *J* = 9.8 Hz, 3H), 1.89 (s, 1H), 1.69 – 1.48 (m, 2H), 1.41 (dd, *J* = 17.1, 10.2 Hz, 15H), 1.31 (s, 9H), 1.22 – 0.75 (m, 2H). ^{13}C NMR (126 MHz, CD_3OD) δ 174.16, 174.01, 173.64, 173.45, 173.28, 172.60, 171.52, 170.67, 164.86, 158.54, 158.42, 155.58, 155.32, 136.94, 136.15, 134.88, 134.83, 133.85, 133.79, 133.73, 132.69, 131.40, 131.26, 131.10, 129.18, 129.07, 129.02, 128.99, 128.64, 128.52, 128.48, 128.43, 127.19, 127.12, 126.67, 126.55, 126.21, 125.62, 125.53, 125.24, 118.66, 87.78, 80.10, 79.95, 79.84, 79.56, 66.63, 61.90, 57.45, 57.20, 53.23, 51.97, 44.51, 43.95, 43.90, 43.84, 41.23, 40.72, 39.51, 38.89, 37.96, 37.12, 36.94, 36.54, 31.65, 31.12, 30.83, 30.22, 29.26, 29.23, 29.16, 28.87, 28.85, 28.83, 28.68, 28.66, 28.65, 28.22, 27.76, 27.72, 27.16, 26.34, 19.65, 19.63, 18.43, 12.56, 12.53. HRMS calc. for $C_{58}H_{79}N_9O_{11}S$ $[M+Na]^+$: 1132.5517 Found: 1132.5519.

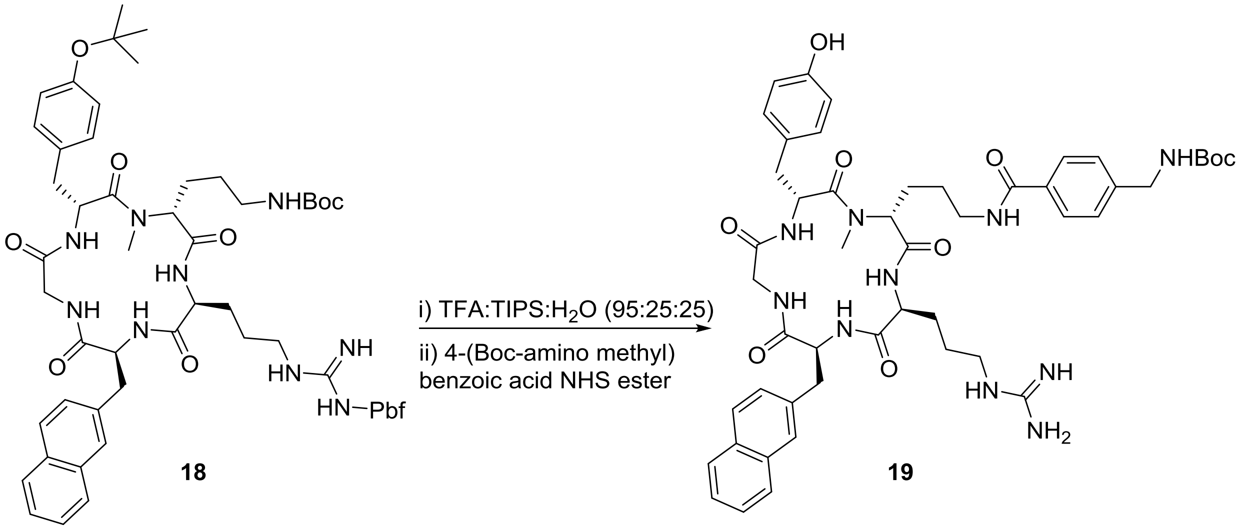

Cyclo(-D-Tyr-(α-methyl, δ-4-(tert-butyl-aminomethyl)-D-Orn-R-Nal-G)) **(19):** Compound **18** was dissolved in TFA, TIPS and H_2O (95:2.5:2.5) and stirred at room temperature for 2 h. The solvents were removed under reduced pressure and the crude product was dried under high vacuum overnight. The amine salt (1 eq) was dissolved in DMF and DIPEA (10 eq) was added, followed by addition of 4-(Boc-amino methyl)benzoic acid NHS ester (2 eq). The resulting reaction mixture was stirred at room temperature for 2.5 h. The DMF was removed under reduced pressure and the crude product was re-dissolved in DMF (3 mL) and loaded onto a preparative HPLC column. The fractions containing compound **19** were collected and lyophilized *in vacuo* to afford **19** as a white powder and a mixture of rotomers. ^{1}H NMR (500 MHz, CD_3OD) δ 7.84 – 7.77 (m, 5H), 7.69 (s, 1H), 7.47 – 7.37 (m, 5H), 7.08 (dd, *J* = 8.2, 4.8 Hz, 2H), 6.72 (dd, *J* = 16.7, 8.1 Hz, 2H), 5.07 – 4.89 (m, 1H), 4.80 – 4.42 (m, 1H), 4.35 – 4.22 (m, 3H), 4.01 – 3.89 (m, 1H), 3.66 – 3.56 (m, 1H), 3.53 – 3.36 (m, 2H), 3.18 (ddd, *J* = 33.6, 13.5, 8.9 Hz, 2H), 2.99 (s, 3H), 2.81 (ddt, *J* = 12.6, 9.8, 4.6 Hz, 2H), 2.72 (m, 3H), 2.09 – 1.85 (m, 1H), 1.61 (m, 16.4, 3H), 1.47 (m, 11H), 1.34 – 0.91 (m, 3H). ^{13}C NMR (126 MHz, CD_3OD) δ 174.68, 174.21, 173.96, 173.59, 173.34, 172.91, 172.41, 171.52, 170.87, 170.45, 170.15, 158.48, 158.36, 157.67, 157.47, 145.25, 145.03, 136.95, 136.18, 135.00, 134.94, 134.22, 133.96, 133.83, 131.74, 131.66, 129.33, 129.25, 129.07, 129.01, 128.81, 128.72, 128.66, 128.52, 128.49, 128.18, 127.28, 127.16, 126.79, 126.63, 116.51, 116.36, 80.45, 66.51, 61.76, 57.45, 56.73, 55.99, 54.79, 53.41, 52.41, 44.73, 44.45, 43.84, 41.79, 41.42, 40.74, 39.98, 39.47, 38.65, 37.87, 37.20, 36.66, 30.65, 30.46, 29.32, 28.80, 27.82, 27.63, 26.45, 25.96, 25.91. HRMS calc. for $C_{49}H_{62}N_{10}O_9$ $[M+H]^+$: 935.4779 Found: 935.4774.

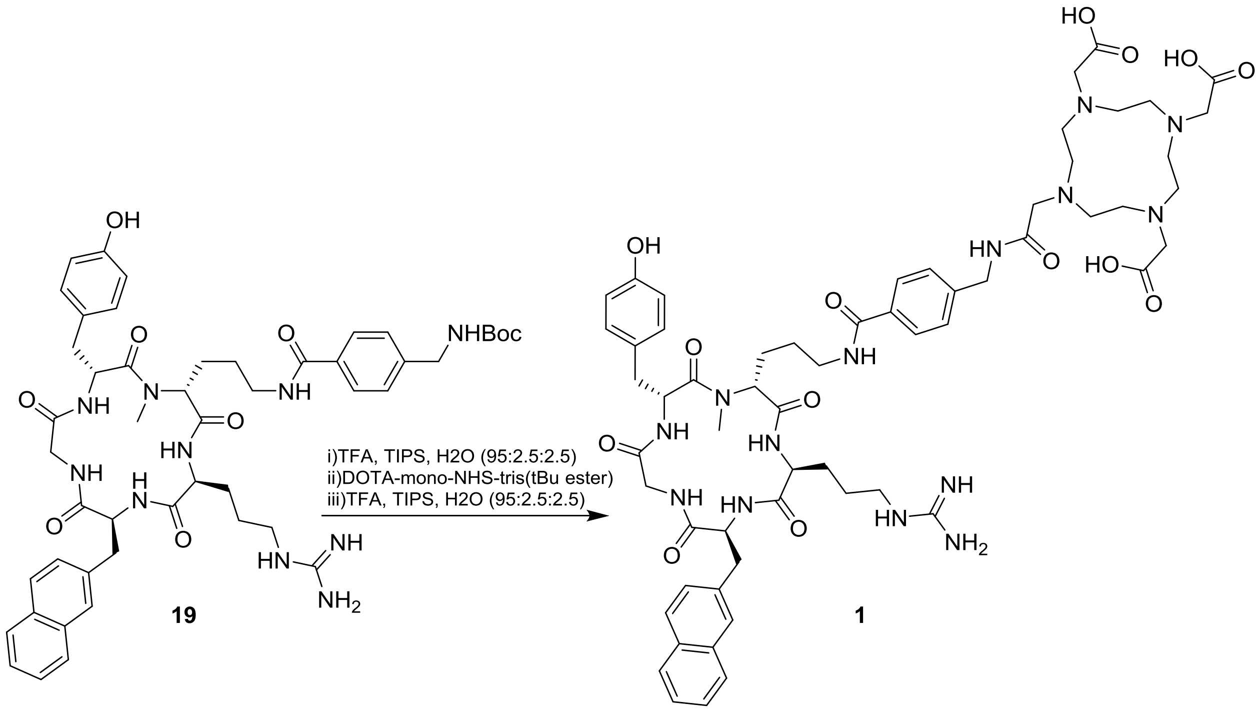

Cyclo(-D-Tyr-(α-methyl, δ-4-(aminomethyl)benzoic acid, DOTA)-D-Orn-R-Nal-G) **(1; pentixafor)**: Compound **19** was dissolved in H_2O, TIPS and TFA (2.5:2.5:95) and stirred at room temperature for 2 h. The solvents were removed under reduced pressure and the residue was dried overnight under high vacuum and used without further purification. To a mixture of amine (1 eq) and DIPEA (10 eq) in DMF was added DOTA-mono-NHS-tris(*t*Bu ester) (2 eq) and the reaction was stirred for 2.5 h. The DMF was removed and the crude product was treated with H_2O (0.025 mL), TIPS (0.025 mL) and TFA (0.95 mL). The resulting mixture was stirred for 2 h. The DMF was removed under reduce pressure and the residue was re-dissolved in DMF (3 mL) and loaded onto a preparative HPLC column. The fractions containing **1** were collected and lyophilized in vacuo to afford pentixafor as a white powder. ^{1}H NMR (500 MHz, CD_3OD) δ 7.80 (td, *J* = 10.0, 9.2, 5.1 Hz, 5H), 7.69 (s, 1H), 7.44 (qd, *J* = 7.1, 6.4, 1.9 Hz, 5H), 7.07 (d, *J* = 8.0 Hz, 2H), 6.77 – 6.67 (m, 2H), 5.04 – 4.93 (m, 1H), 4.47 (t, *J* = 9.0 Hz, 2H), 4.37 – 4.26 (m, 1H), 4.06 – 3.51 (m, 10H), 3.41 (ddt, *J* = 28.9, 11.3, 6.5 Hz, 10H), 3.31 – 3.11 (m, 11H), 3.00 (d, *J* = 14.3 Hz, 3H), 2.85 (d, *J* = 28.9 Hz, 1H), 2.74 (s, 2H), 2.09 – 1.90 (m, 1H), 1.73 – 1.55 (m, 2H), 1.54 – 1.24 (m, 2H), 1.21 (s, 1H), 1.09 (d, *J* = 46.1 Hz, 2H). ^{13}C NMR (126 MHz, CD_3OD) δ 173.23, 172.74, 172.64, 172.20, 171.96, 171.34, 170.94, 170.08, 169.42, 168.92, 168.64, 161.56, 161.28, 161.00, 160.72, 157.05, 156.93, 156.18, 155.98, 135.50, 134.69, 133.54, 133.49, 132.51, 132.38, 130.31, 130.24, 127.99, 127.81, 127.64, 127.56, 127.48, 127.36, 127.27, 127.21, 127.17, 127.06, 127.03, 125.84, 125.70, 125.18, 120.07, 117.75, 115.43, 115.06, 114.93, 64.97, 60.27, 55.99, 55.38, 54.77, 54.56, 53.32, 51.96, 51.00, 49.86, 48.22, 48.11, 48.05, 47.94, 47.88, 47.77, 47.71, 47.60, 47.54, 47.43, 47.25, 47.12, 47.08, 43.04, 42.46, 40.31, 40.13, 39.27, 38.47, 37.99, 37.22, 36.35, 35.81, 35.16, 29.19, 29.12, 27.97, 26.41, 26.05, 25.79, 25.02, 24.56, 24.41. HRMS calc. for $C_{60}H_{80}N_{14}O_{14}$ $[M+H]^+$: 1221.6052 Found: 1221.6057.

S003. Inhibition Binding Assays

Figure S7. Determination of IC_{50} in a competition assay vs [^{68}Ga]Pentixafor in PC3-CXCR4 cells. The concentration of [^{68}Ga]Pentixafor was 100 nM.

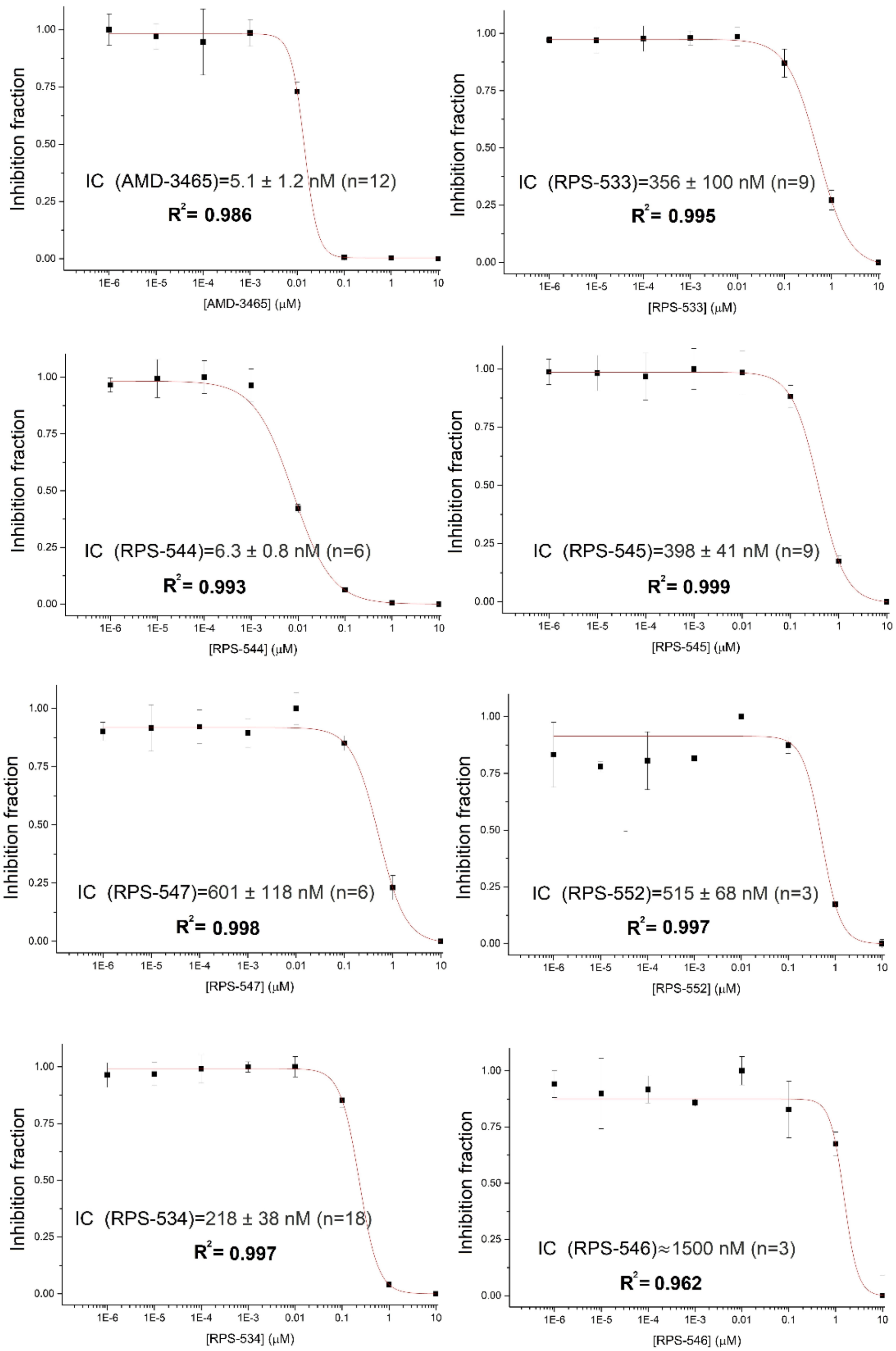

S004. Saturation Binding Assays

Figure S8. Determination of dissociation constants (Kd) in PC3-CXCR4 cells. Radiolabeled ligands were added at a concentration of approximately 5 pM. **A.** Saturation binding curves plotted using nonlinear curve fit (one sit binding). **B.** Curves plotted using a Hill plot.

A.

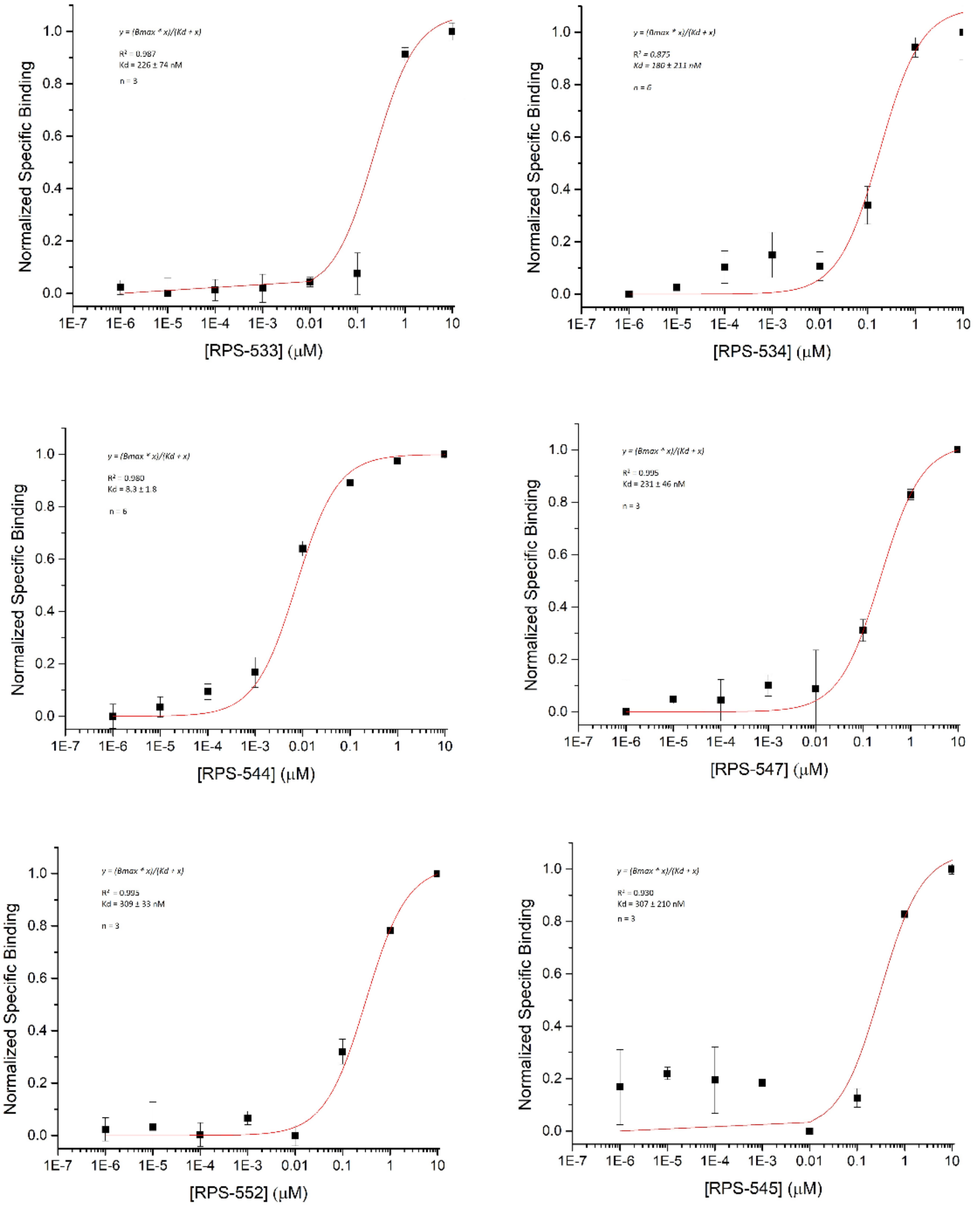

B.

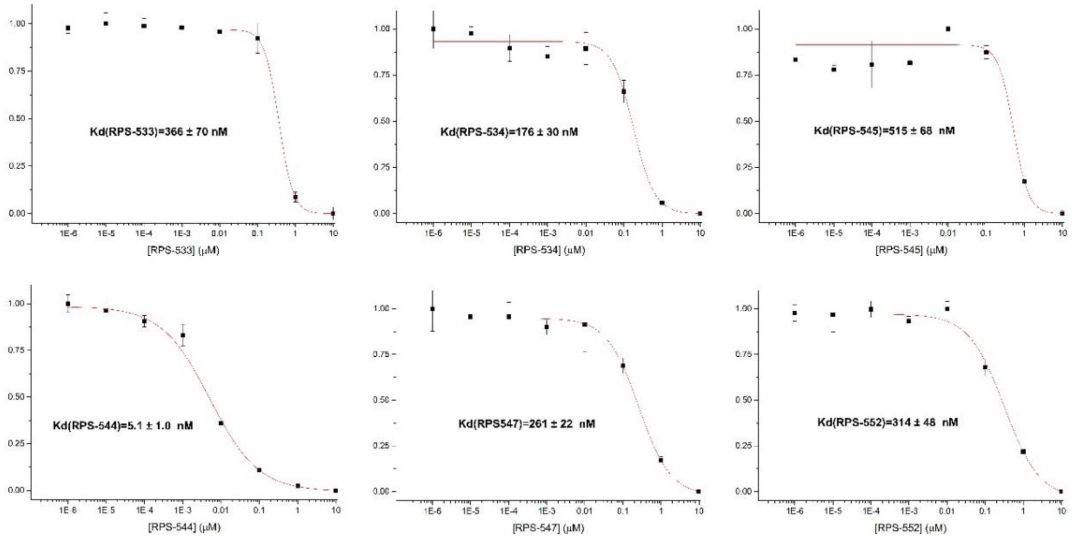

Table S2. Hill slopes determined from the competition assay vs [^{68}Ga]Pentixafor in PC3-CXCR4 cells and the saturation binding assays in PC3-CXCR4 cells. Slopes are expressed as value ± SD. AMD-3465 was not included in the saturation binding assays.

**Compound**	**Hill Slope**	
	Competitive Binding Experiment	*Saturation Binding Experiment*
AMD-3465	1.01 ± 0.30	n.d.
RPS-544	1.13 ± 0.47	1.06 ± 0.11
RPS-533	1.07 ± 0.36	1.12 ± 0.05
RPS-534	1.15 ± 0.34	1.05 ± 0.15
RPS-545	1.75 ± 0.33	2.01 ± 0.22
RPS-546	0.96 ± 0.10	0.98 ± 0.19
RPS-547	0.97 ± 0.14	1.03 ± 0.08
RPS-552	1.18 ± 0.07	0.93 ± 0.14

S005. Internalization Experiments

Figure S9. Internalization of [^{18}F]RPS-534 and [^{18}F]RPS-547 in PC3-CXCR4 cells. The ligands were incubated at 4°C or 37°C for the corresponding time. Surface-bound activity was removed by successive washes with 50 mM glycine (pH = 2.8), and internalized activity was collected by detaching the cells with 1 M NaOH.

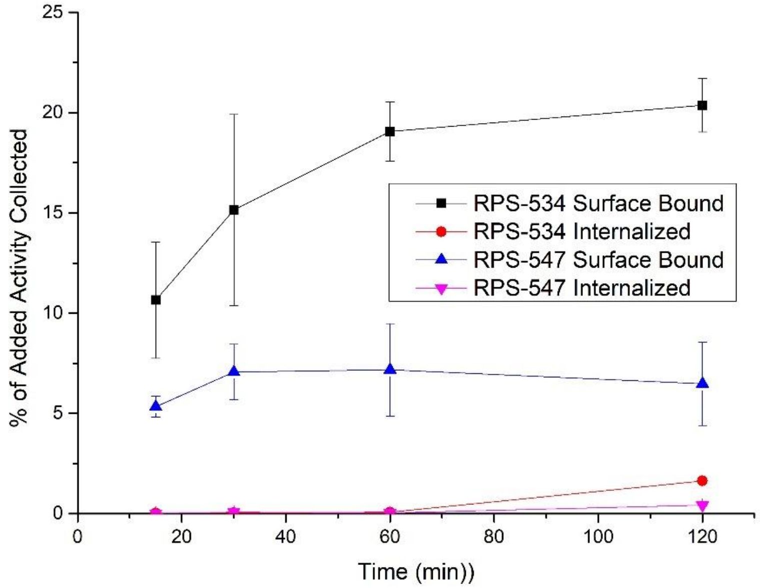

S006. Tissue Biodistribution

Table S2. Table of tissue activity values from biodistribution studies conducted in male BALB/c mice bearing bilateral PC3-WT/PC3-CXCR4 tumors.

Tissue	RPS-533 1h p.i.	RPS-545 1h p.i.	RPS-534 1h p.i.	RPS-534 1h p.i. Blocked	RPS-534 2h p.i.
Blood	0.30±0.06	0.20±0.03	0.30±0.08	0.26±0.15	0.08±0.02
Heart	0.57±0.05	0.14±0.02	0.25±0.01	0.13±0.04	0.14±0.02
Lungs	0.60±0.08	0.77±0.01	1.01±0.09	0.48±0.08	0.59±0.03
Liver	3.69±0.21	23.90±1.79	19.14±0.42	12.01±4.67	18.60±1.74
Small Intestine	2.19±0.05	0.32±0.02	3.67±0.44	5.35±1.74	1.53±0.25
Large Intestine	0.23±0.02	0.08±0.00	0.29±0.06	0.40±0.58	4.60±0.57
Stomach	0.17±0.04	0.09±0.01	0.16±0.03	0.14±0.11	0.12±0.05
Spleen	0.52±0.02	4.70±0.80	2.49±0.04	0.12±0.03	1.90±0.23
Pancreas	0.44±0.02	0.13±0.02	0.28±0.02	0.32±0.01	0.18±0.06
Kidneys	12.17±0.78	3.04±0.15	7.43±0.96	5.10±0.33	5.05±0.80
Muscle	0.67±0.08	0.12±0.08	0.19±0.03	0.17±0.16	0.11±0.03
Bone	1.22±0.03	2.43±0.20	1.44±0.04	0.44±0.15	1.26±0.06
PC3-WT	0.91±0.19	0.73±0.06	2.85±0.22	1.21±0.47	1.66±0.08
PC3-CXCR4	1.93±0.18	1.41±0.13	7.20±0.30	2.01±0.93	4.31±0.47
**Tissue**	**RPS-547 1h p.i.**	**RPS-547 1h p.i. Blocked**	**RPS-547 2h p.i.**	**RPS-552 1h p.i.**	**RPS-552 1h p.i. Blocked**
Blood	0.28±0.06	0.41±0.40	0.08±0.01	0.21±0.07	0.27±0.06
Heart	0.18±0.04	0.14±0.19	0.08±0.01	0.13±0.03	0.18±0.08
Lungs	0.48±0.05	0.35±0.15	0.24±0.02	0.50±0.04	0.49±0.09
Liver	6.59±1.00	4.13±0.50	5.18±0.27	4.36±0.24	3.15±0.70
Small Intestine	0.95±0.10	0.51±0.18	0.80±0.01	0.54±0.06	0.80±0.11
Large Intestine	0.20±0.02	0.23±0.06	0.19±0.12	0.43±0.10	0.22±0.03
Stomach	0.17±0.03	0.10±0.03	0.11±0.02	0.14±0.07	0.20±0.20
Spleen	0.53±0.02	0.12±0.03	0.36±0.04	0.92±0.08	0.70±0.11
Pancreas	0.20±0.01	0.22±0.04	0.11±0.00	0.26±0.05	0.25±0.04
Kidneys	10.73±1.10	4.08±1.10	5.60±0.56	7.23±2.66	4.41±1.01
Muscle	0.14±0.01	0.18±0.35	0.09±0.02	0.15±0.04	0.45±0.67
Bone	0.52±0.04	0.22±0.12	0.31±0.02	0.64±0.14	0.31±0.07
PC3-WT	1.54±0.18	0.86±0.10	0.86±0.13	1.00±0.23	0.85±0.21
PC3-CXCR4	3.09±0.52	1.16±0.12	2.02±0.10	2.52±0.11	1.20±0.43

S007. Correlation Between Docking Score and IC_{50}

Figure S10. Linear plot of docking score and IC_{50}. Docking score was determined by an extra precision screen against human CXCR4 (PDB ID: 3ODU) using Schrodinger, and IC_{50} was determined by competitive binding assay vs. [^{68}Ga]Pentixafor in PC3-CXCR4 cells.

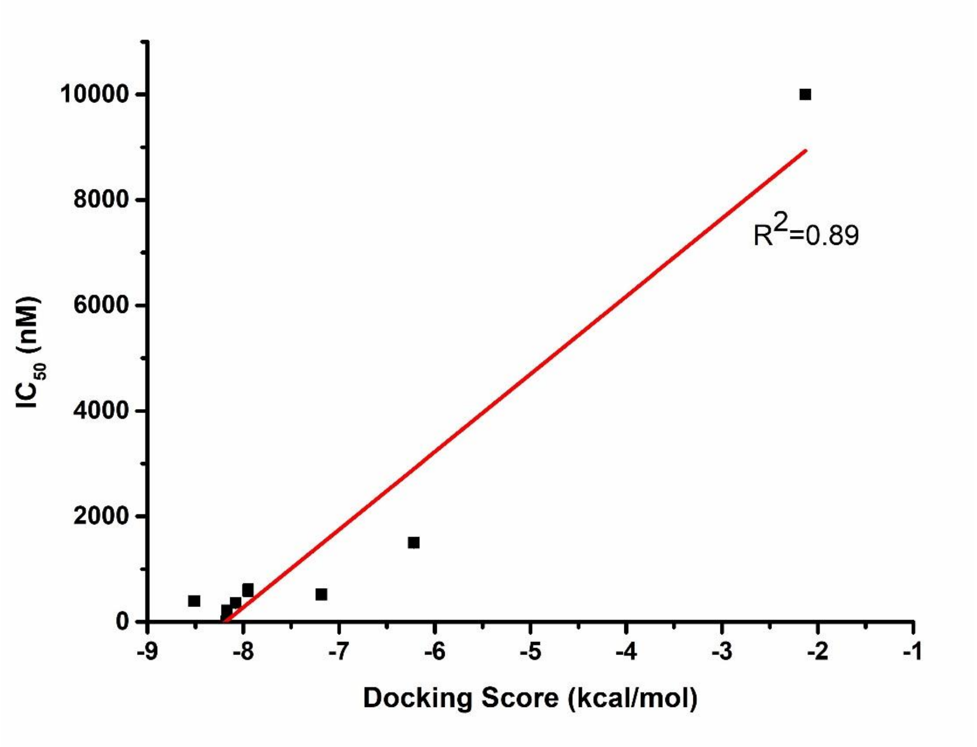

S008. NMR Spectroscopic Data

NMR spectroscopic data are provided for the following compounds:

RPS-533 (1H, 13C, 19F)

RPS-545 (1H, 13C, 19F)

RPS-534 (1H, 13C, 19F)

RPS-547 (1H, 13C, 19F)

RPS-552 (1H, 13C, 19F)

RPS-546 (1H, 13C, 19F)

Pentixafor (1H, 13C)

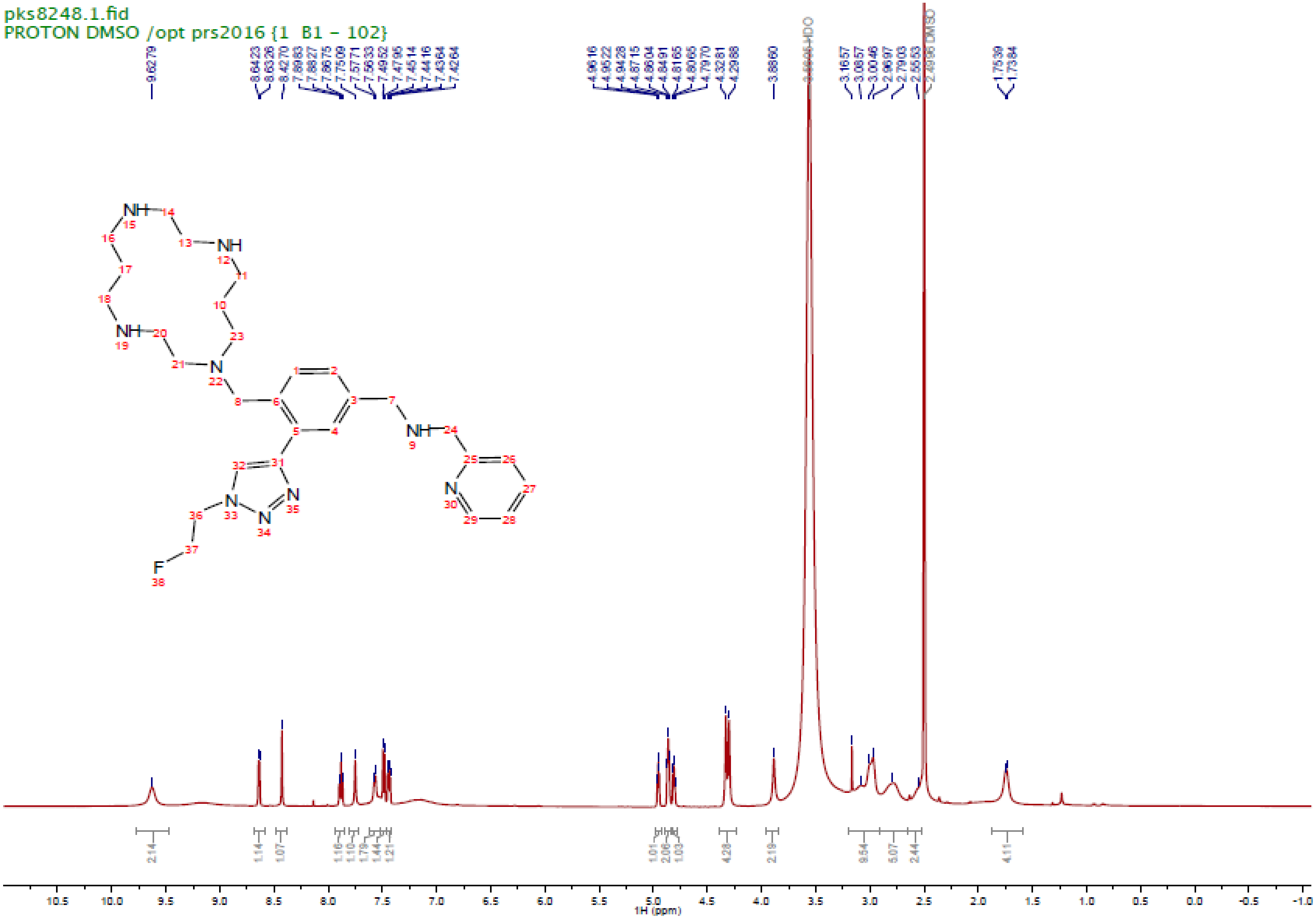
pks8248.1.fid
PROTON DMSO /opt prs2016 {1 B1 - 102}
1H (ppm)

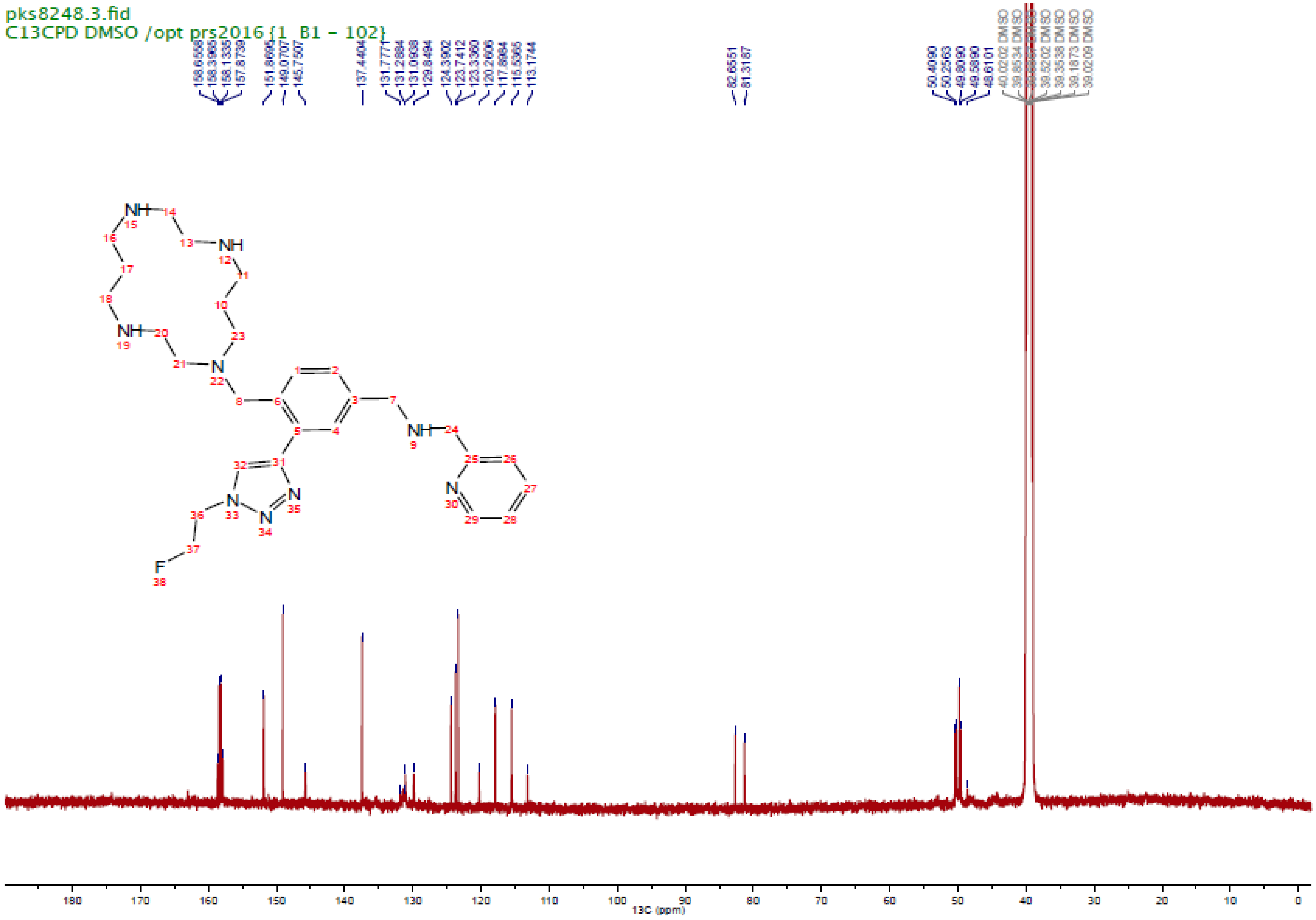
pks8248.3.fid
C13CPD DMSO /opt prs2016 {1 B1 - 102}
158.6558
158.3965
158.1335
157.8739
151.8695
149.0707
145.7507
137.4404
131.7771
131.2884
131.0938
129.8494
124.3902
123.7412
123.3360
120.2606
117.8684
115.5365
113.1744
82.6551
81.3187
50.4090
50.2563
49.8090
49.5890
48.6101
40.0202 DMSO
39.8534 DMSO
39.5202 DMSO
39.3538 DMSO
39.1873 DMSO
39.0209 DMSO
13C (ppm)

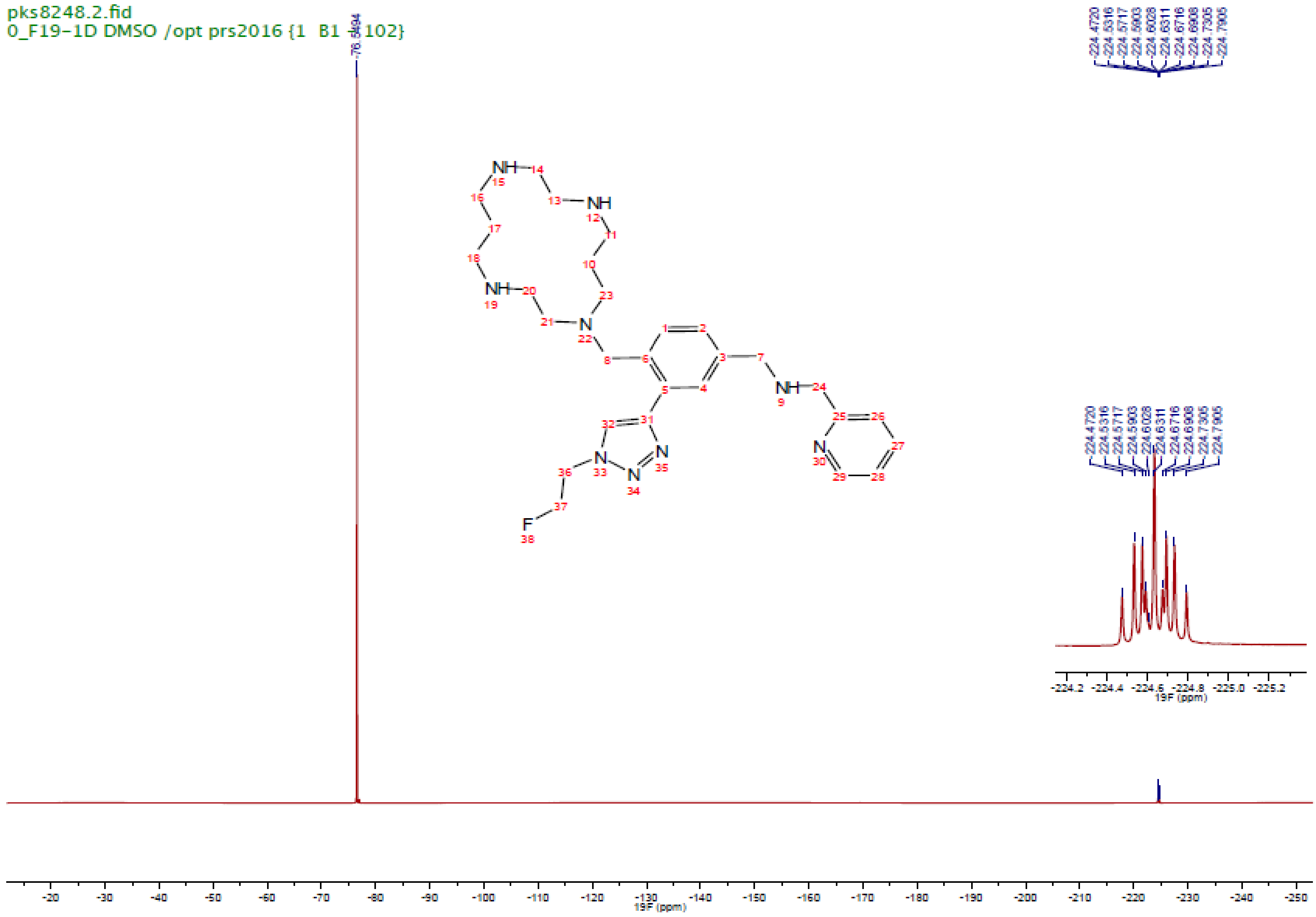

pks8248.2.fid
0_F19-1D DMSO /opt prs2016 {1 B1 102}
-76.5494
224.4720
224.5316
224.5717
224.5903
224.6028
224.6311
224.6716
224.6908
224.7305
224.7905
NH 15
NH 12
NH 19
N 22
NH 9
N 30
N 33
N 34
N 35
F 38
19F (ppm)

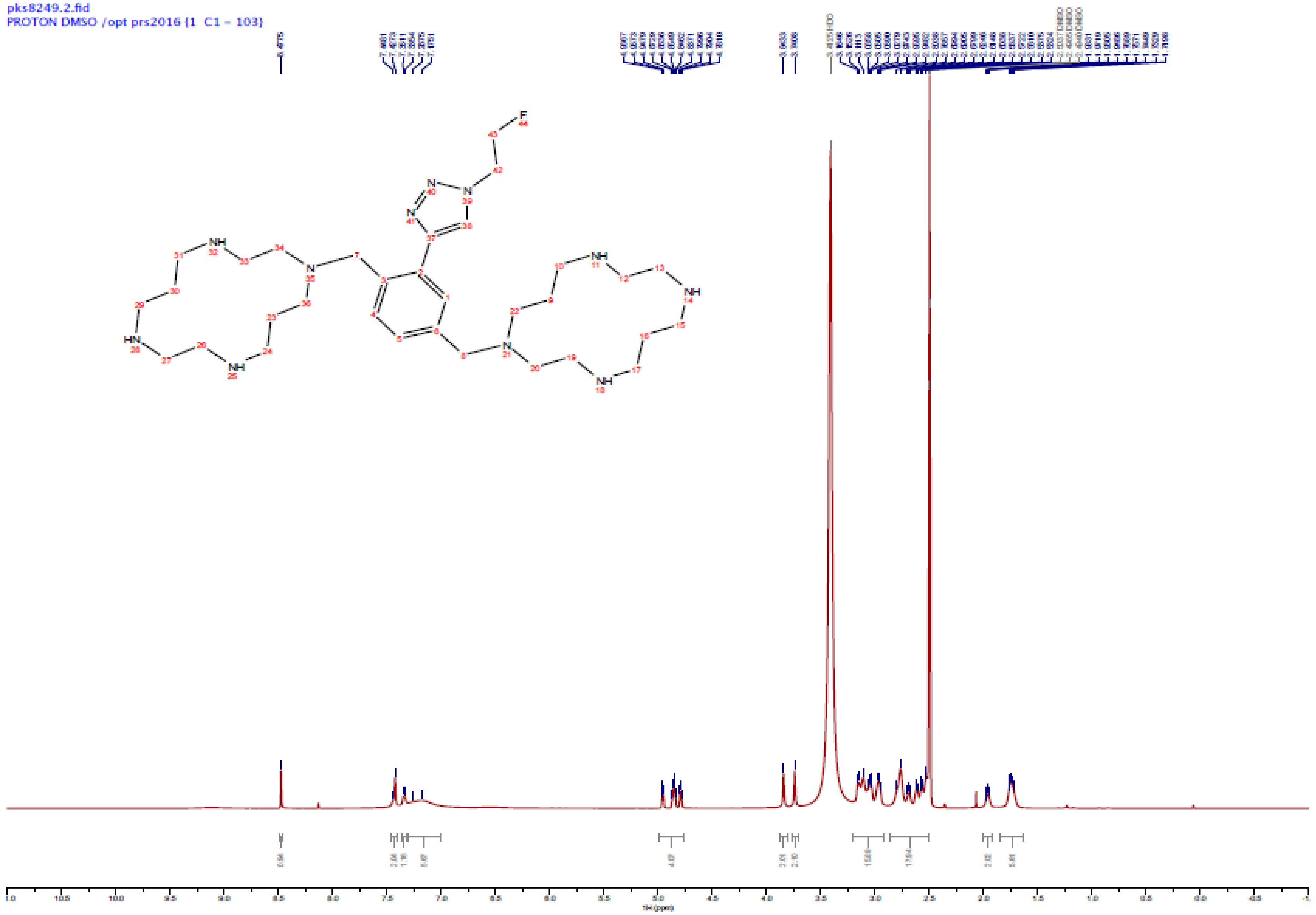
pks8249.2.fid
PROTON DMSO /opt prs2016 {1 C1 - 103}

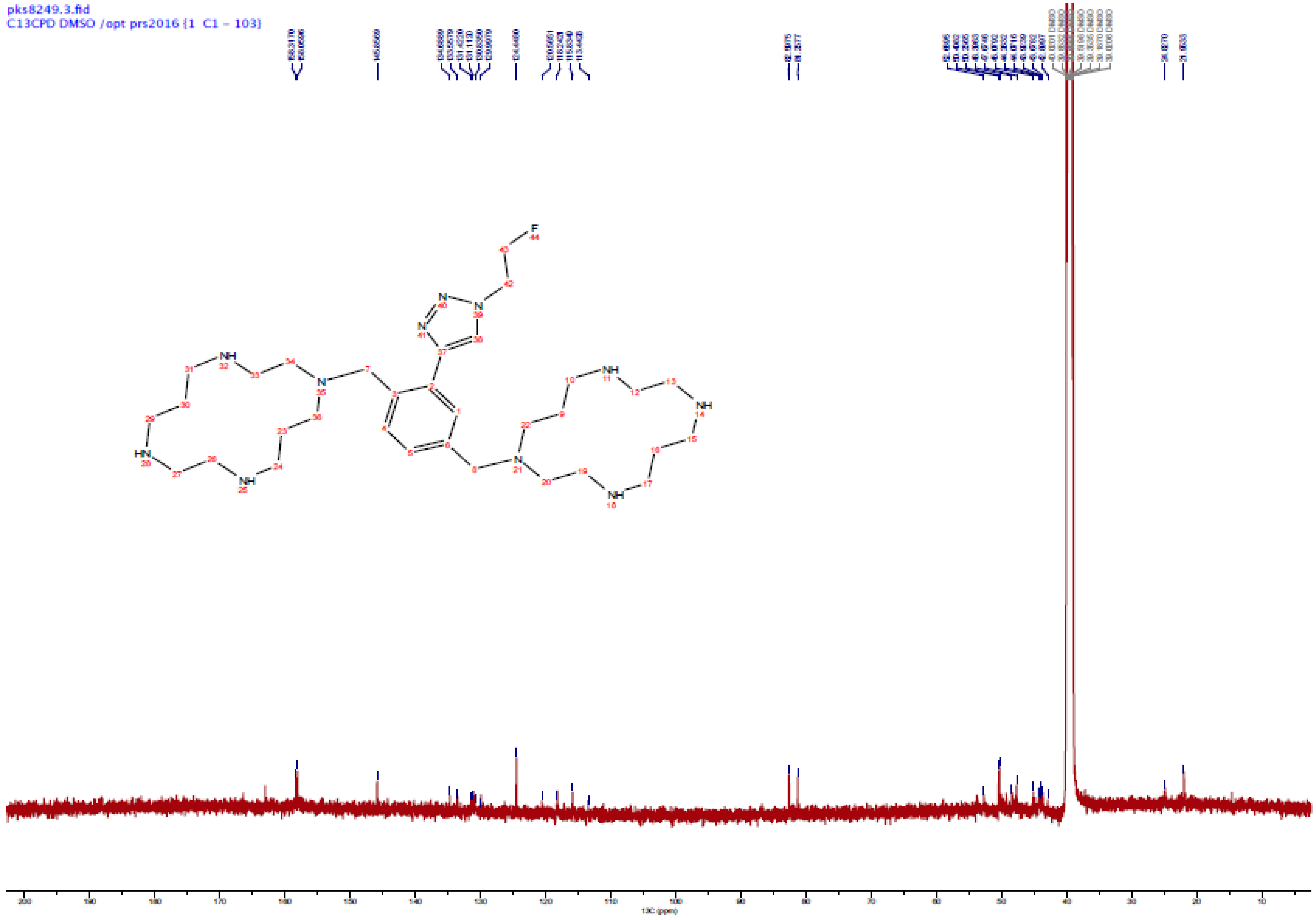
pks8249.3.fid
C13CPD DMSO /opt prs2016 {1 C1 - 103}

pks8249.1.fid
0_F19-1D DMSO /opt prs2016 {1 C1 - 103}

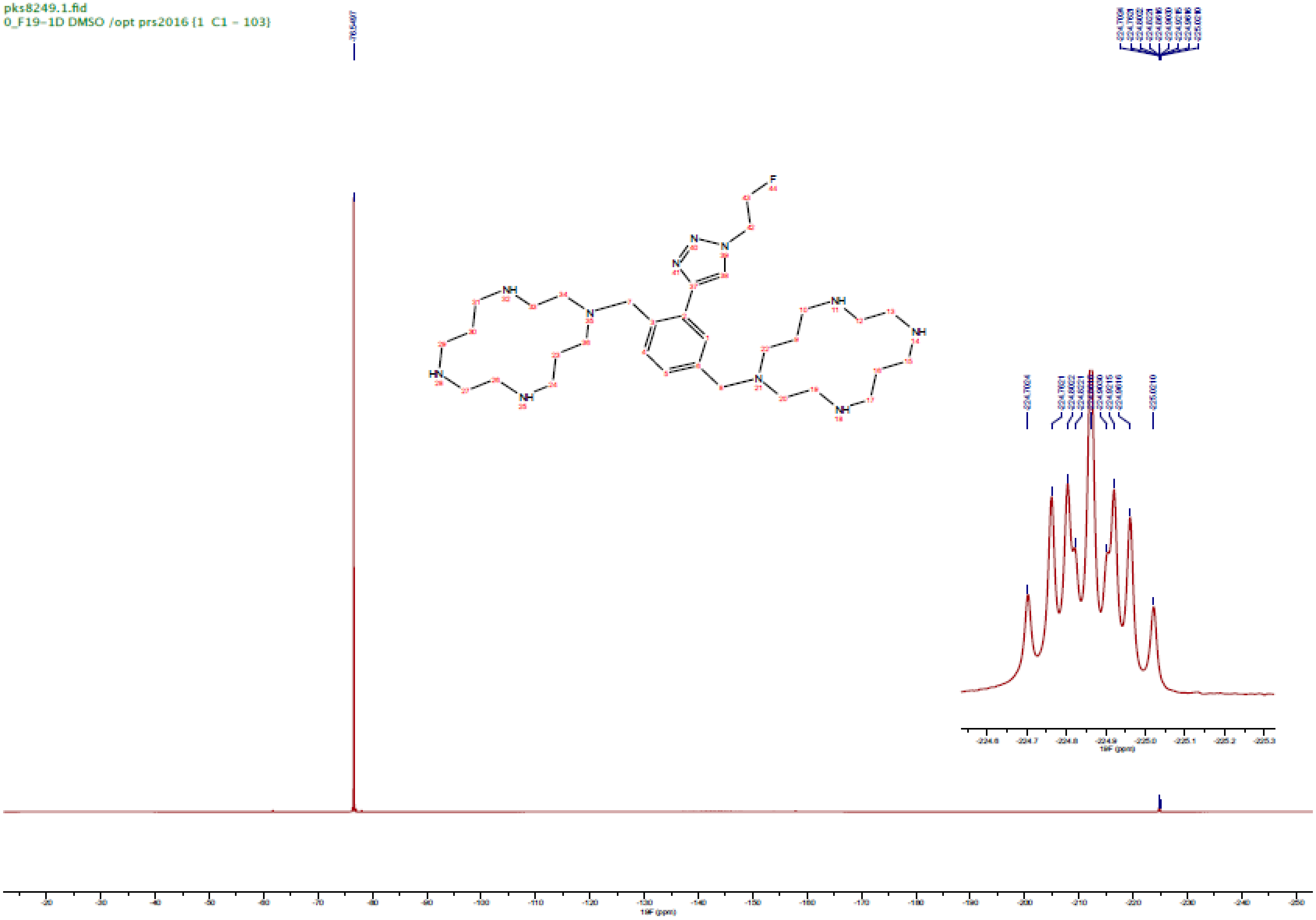

pks8246.1.fid
PROTON DMSO /opt prs2016 {1 A1 - 101}

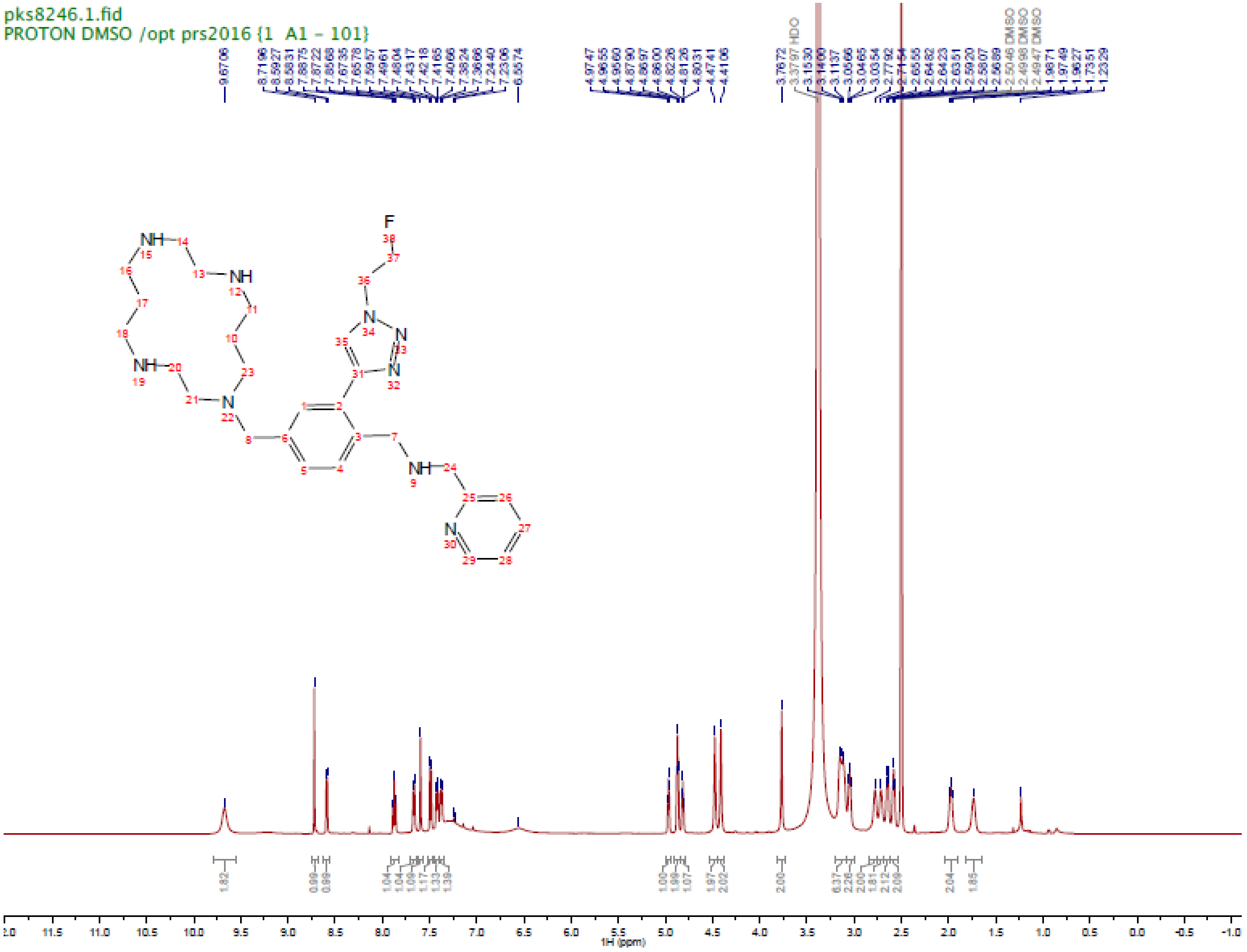

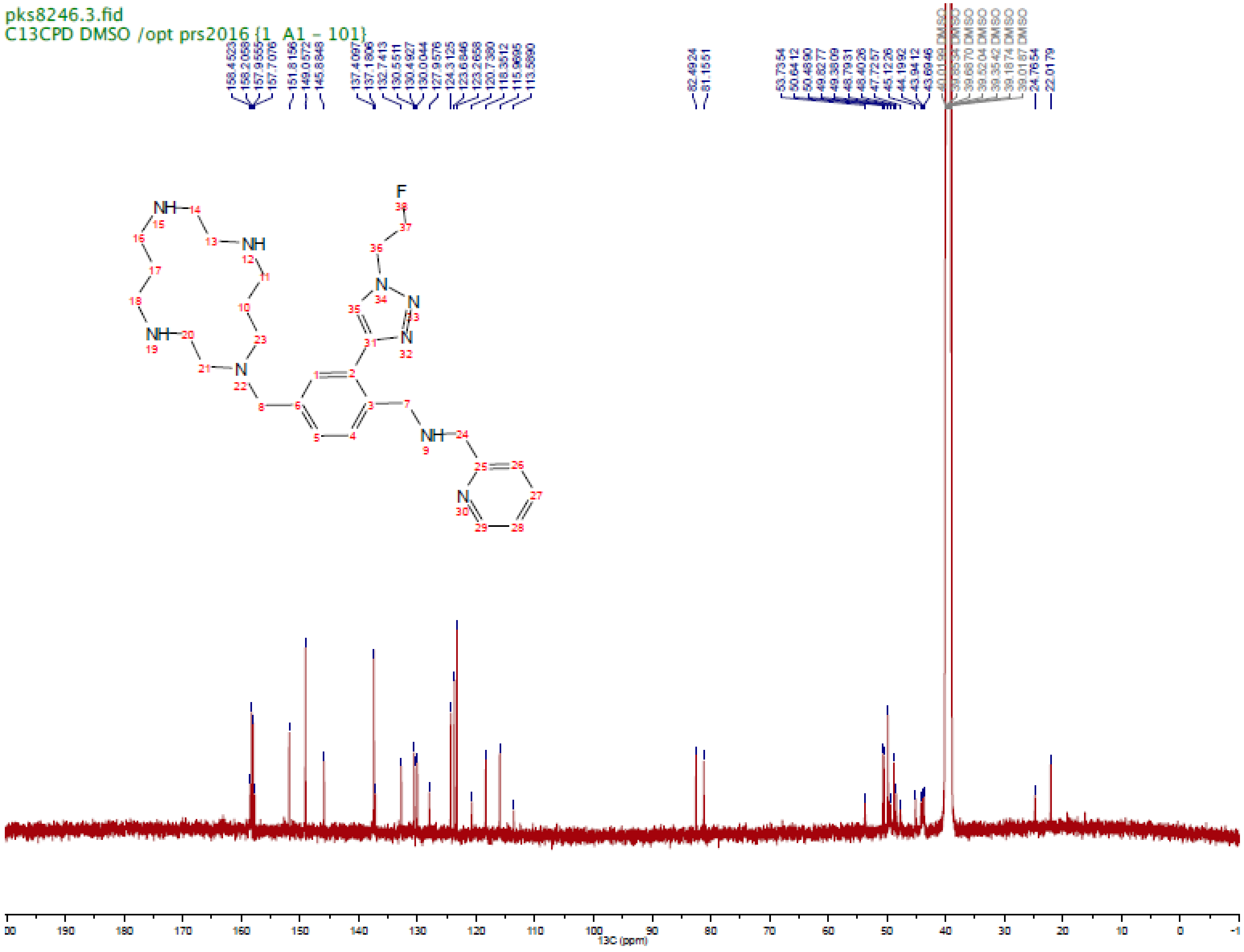
pks8246.3.fid
C13CPD DMSO /opt prs2016 {1 A1 - 101}
13C (ppm)

pks8246.2.fid
0_F19-1D DMSO /opt prs2016 {1 A1 } 101}

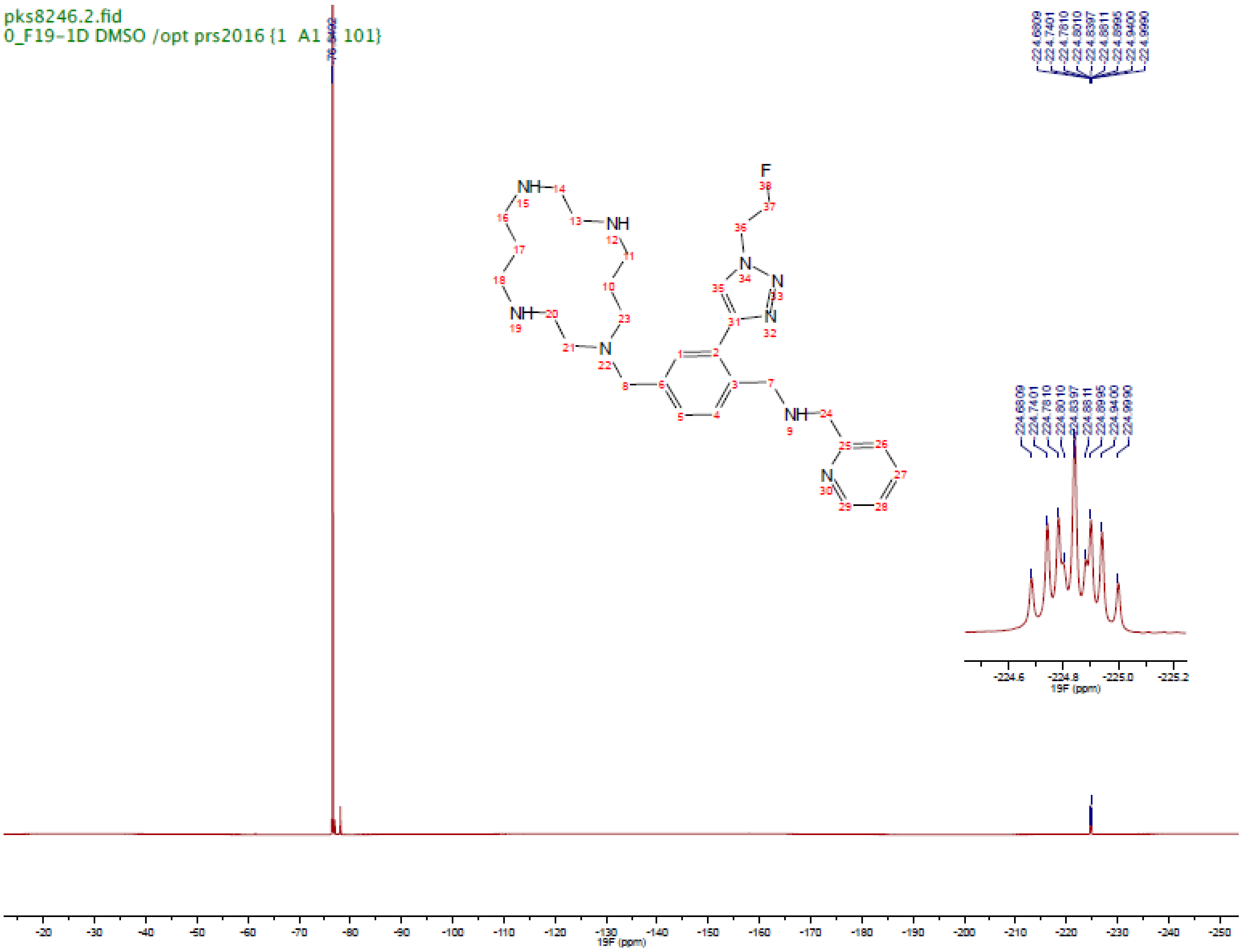

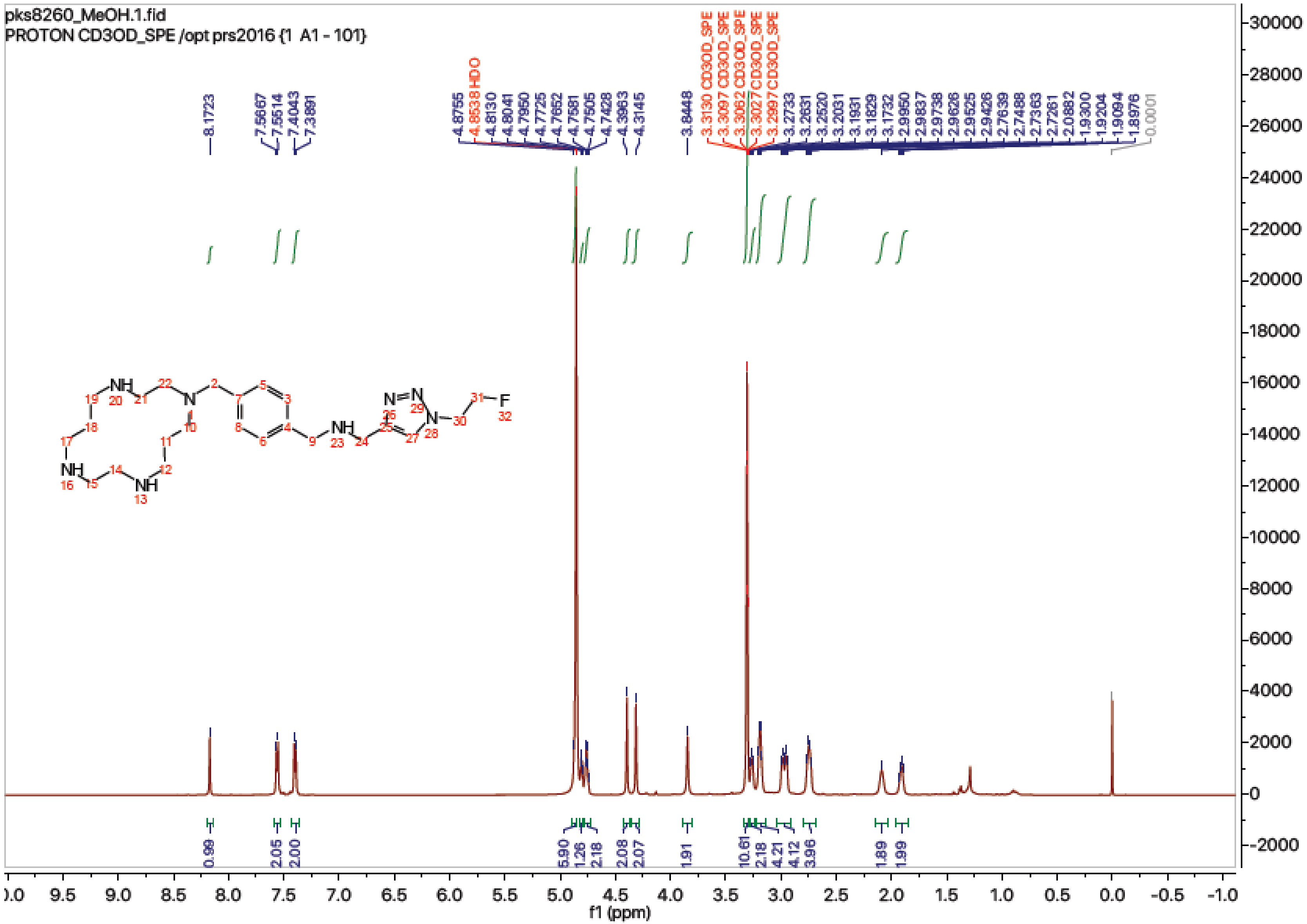
pks8260_MeOH.1.fid
PROTON CD3OD_SPE /opt prs2016 {1 A1 - 101}
f1 (ppm)

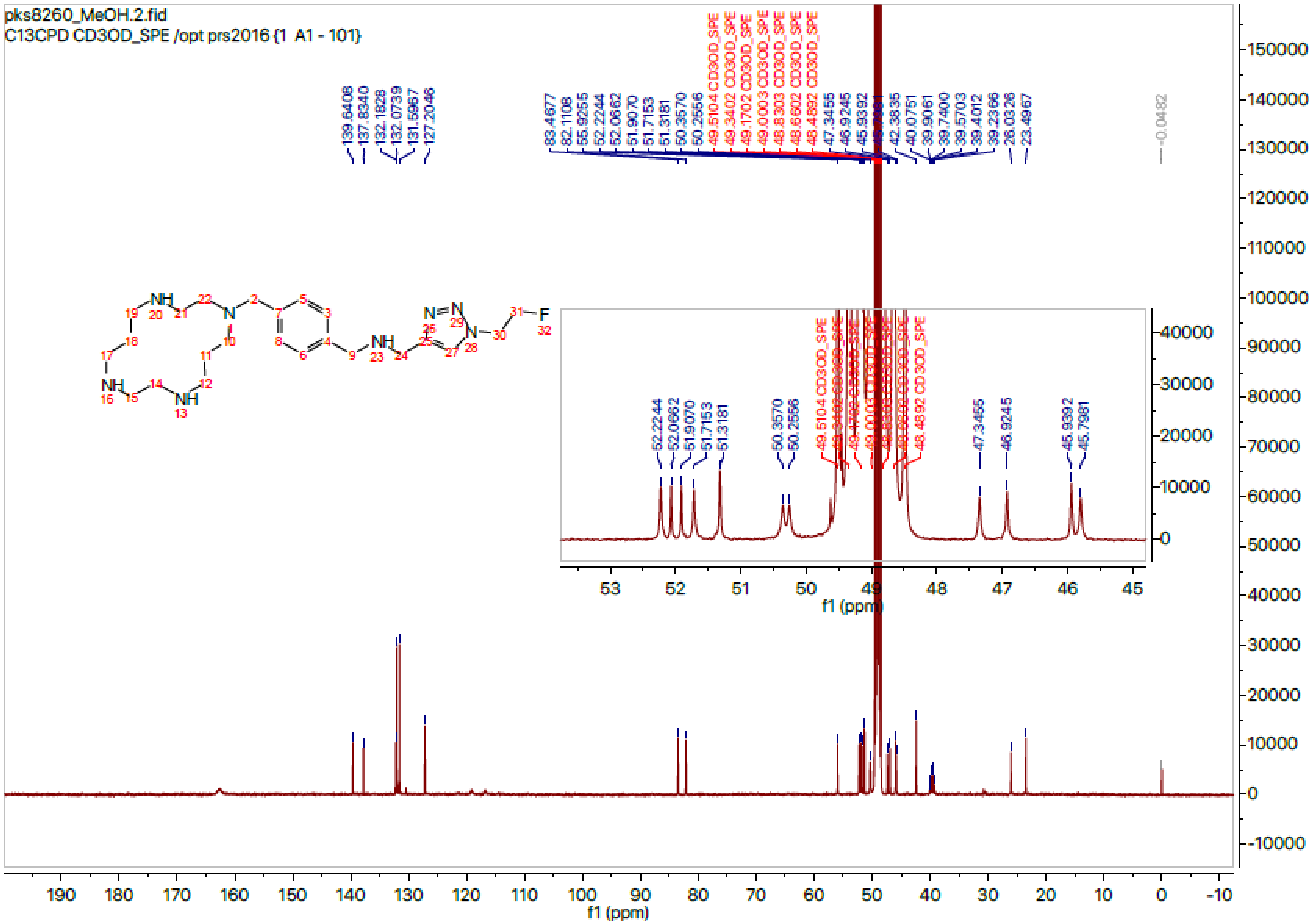
pks8260_MeOH.2.fid
C13CPD CD3OD_SPE /opt prs2016 {1 A1 - 101}
139.6408
137.8340
132.1828
132.0739
131.5967
127.2046
83.4677
82.1108
55.9255
52.2244
52.0662
51.9070
51.7153
51.3181
50.3570
50.2556
49.5104 CD3OD_SPE
49.3402 CD3OD_SPE
49.1702 CD3OD_SPE
49.0003 CD3OD_SPE
48.8303 CD3OD_SPE
48.6602 CD3OD_SPE
48.4892 CD3OD_SPE
47.3455
46.9245
45.9392
42.3835
40.0751
39.9061
39.7400
39.5703
39.4012
39.2366
26.0326
23.4967
-0.0482
f1 (ppm)

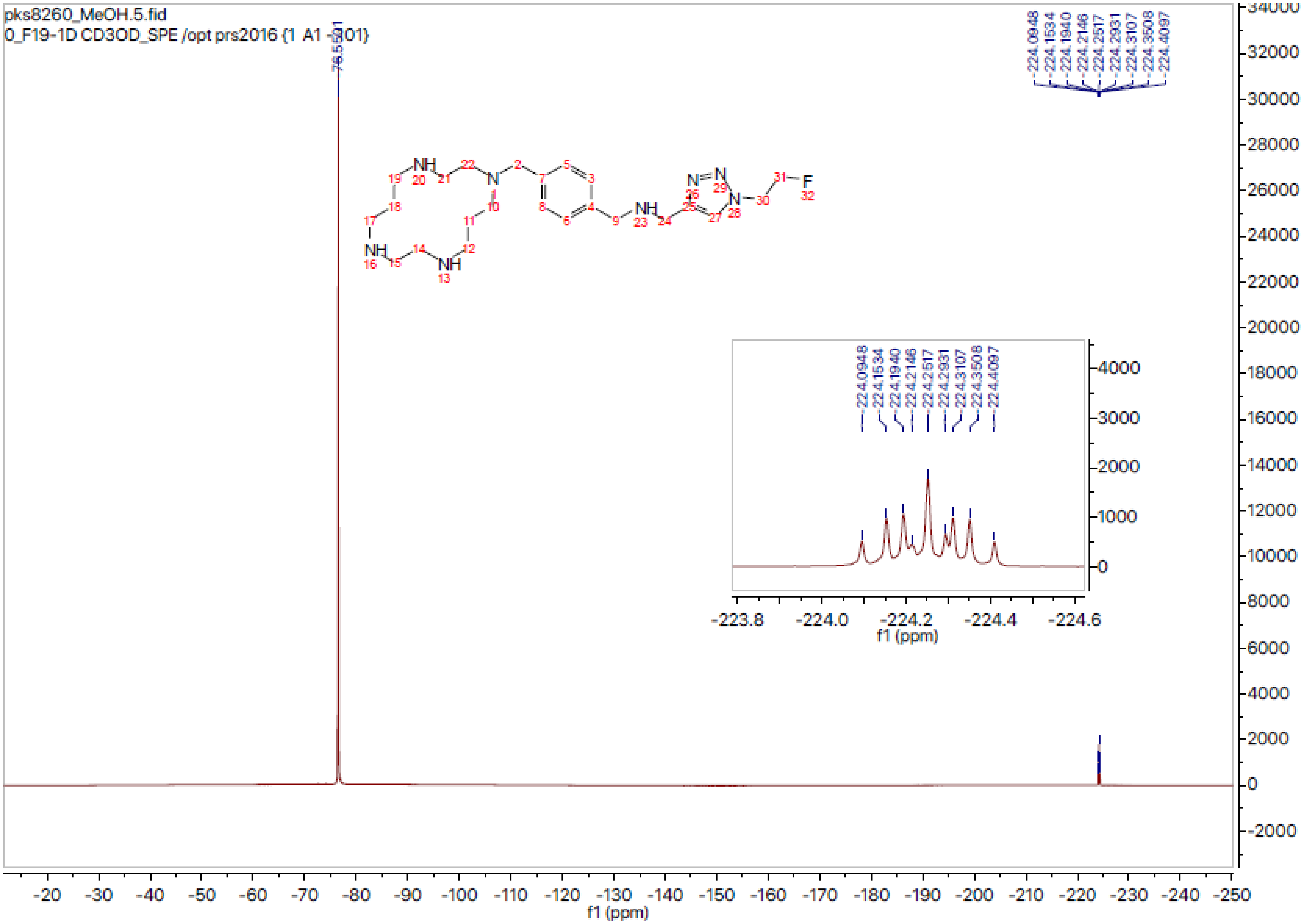
pks8260_MeOH.5.fid
0_F19-1D CD3OD_SPE /opt prs2016 {1 A1 -201}
-224.0948
-224.1534
-224.1940
-224.2146
-224.2517
-224.2931
-224.3107
-224.3508
-224.4097
f1 (ppm)

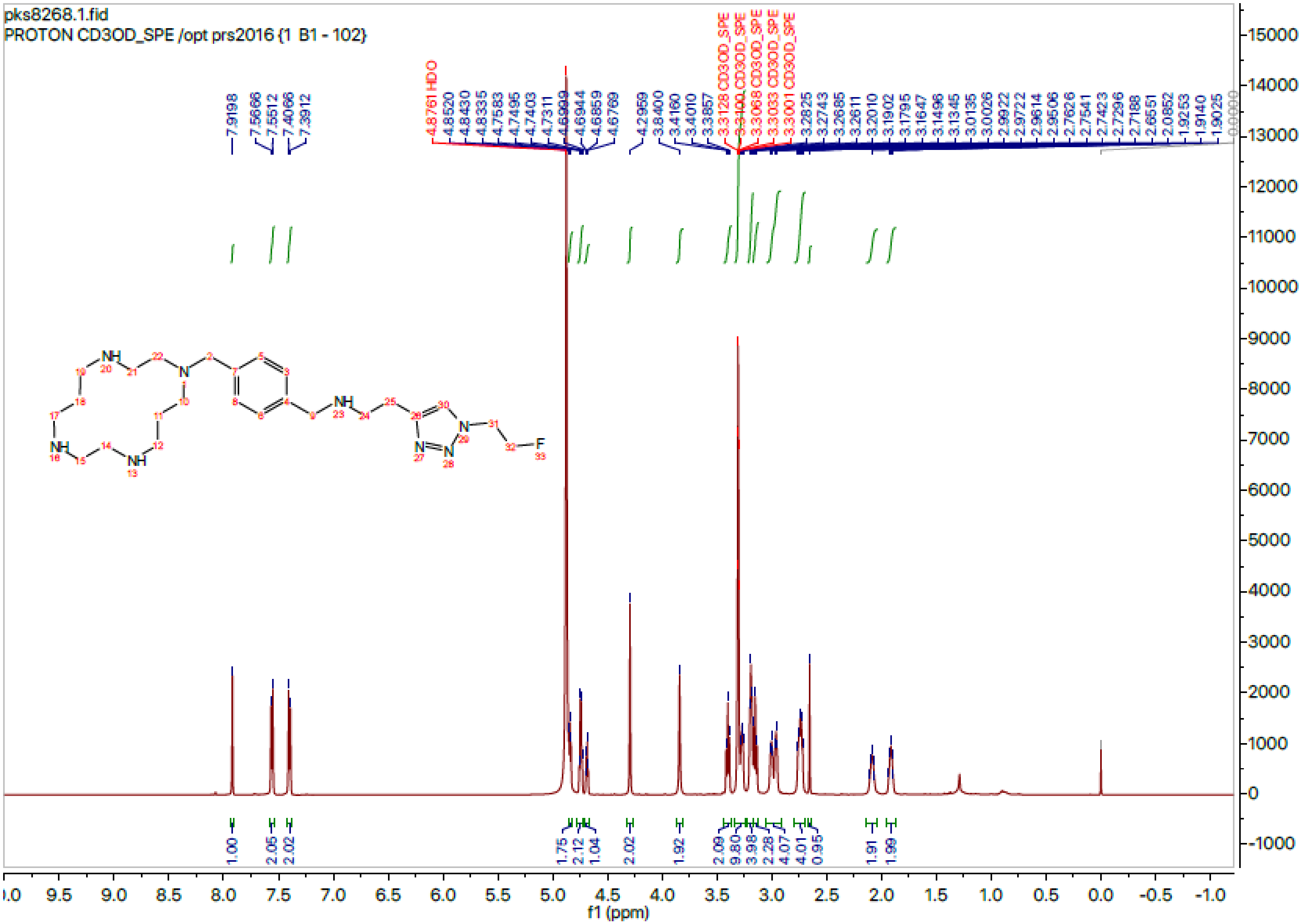

pks8268.1.fid
PROTON CD3OD_SPE /opt prs2016 {1 B1 - 102}
f1 (ppm)

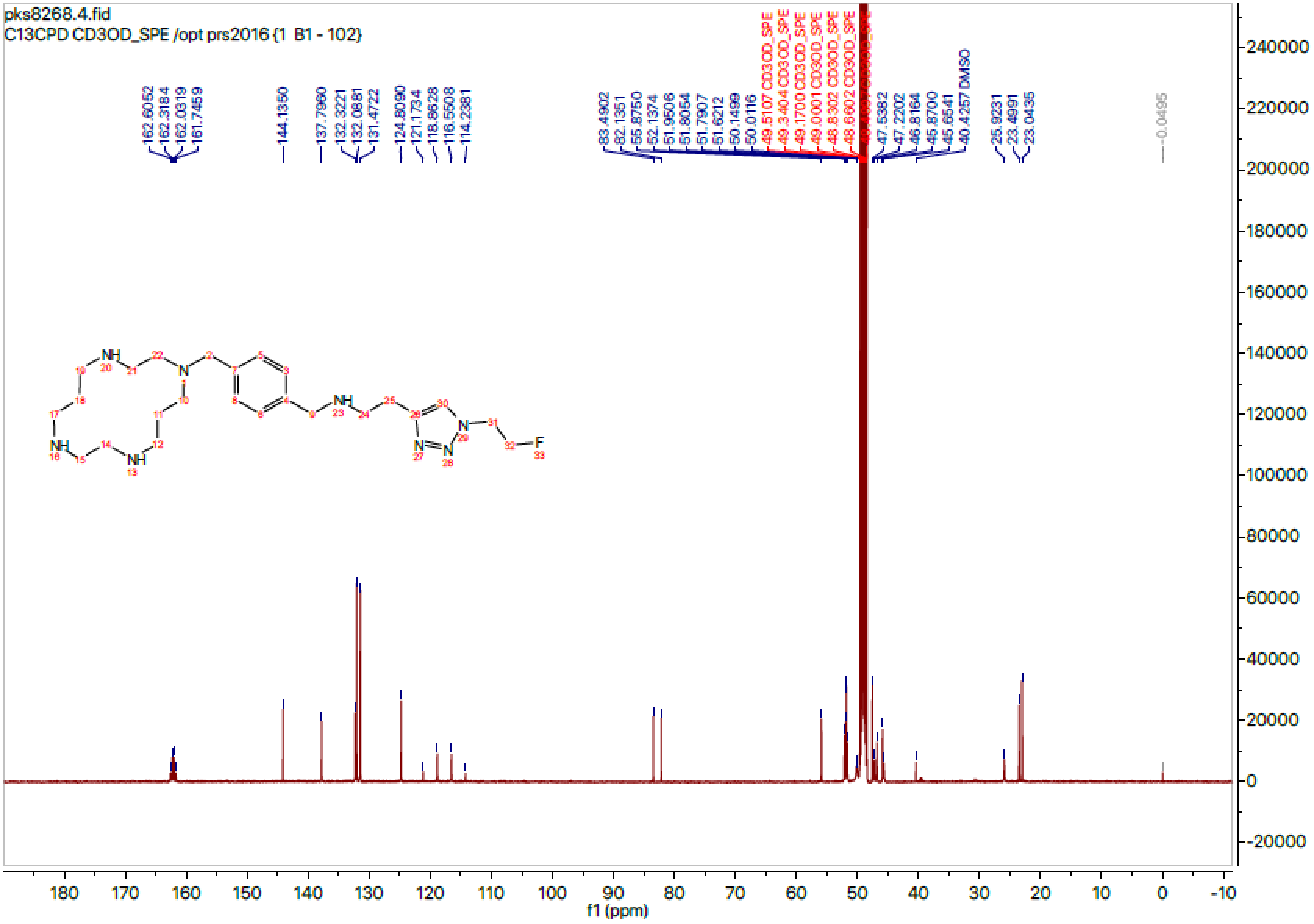
pks8268.4.fid
C13CPD CD3OD_SPE /opt prs2016 {1 B1 - 102}
162.6052
162.3184
162.0319
161.7459
144.1350
137.7960
132.3221
132.0881
131.4722
124.8090
121.1734
118.8628
116.5508
114.2381
83.4902
82.1351
55.8750
52.1374
51.9506
51.8054
51.7907
51.6212
50.1499
50.0116
49.5107 CD3OD_SPE
49.3404 CD3OD_SPE
49.1700 CD3OD_SPE
49.0001 CD3OD_SPE
48.8302 CD3OD_SPE
48.6602 CD3OD_SPE
47.5382
47.2202
46.8164
45.8700
45.6541
40.4257 DMSO
25.9231
23.4991
23.0435
-0.0495
f1 (ppm)

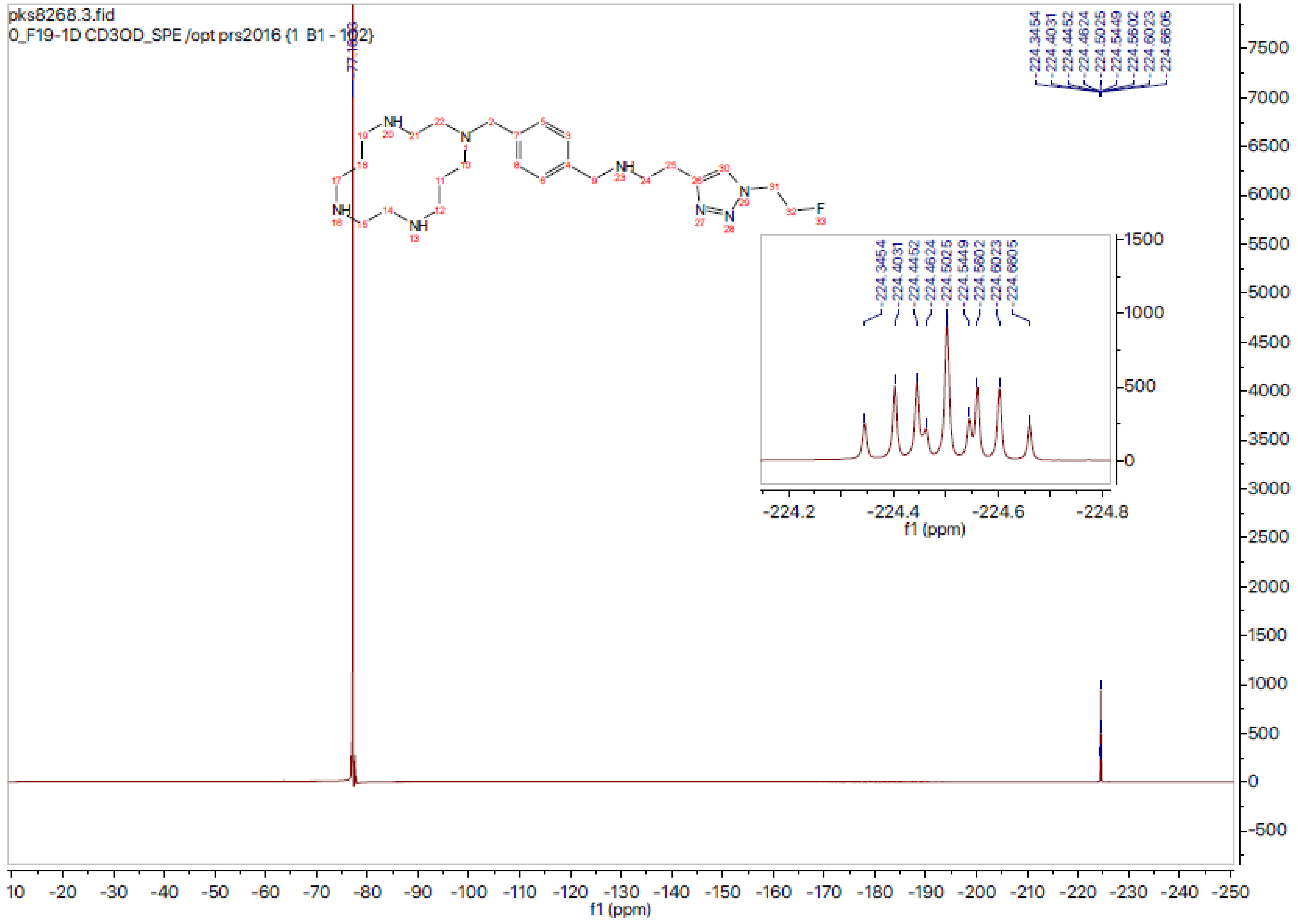
pks8268.3.fid
0_F19-1D CD3OD_SPE /opt prs2016 {1 B1 - 102}
f1 (ppm)

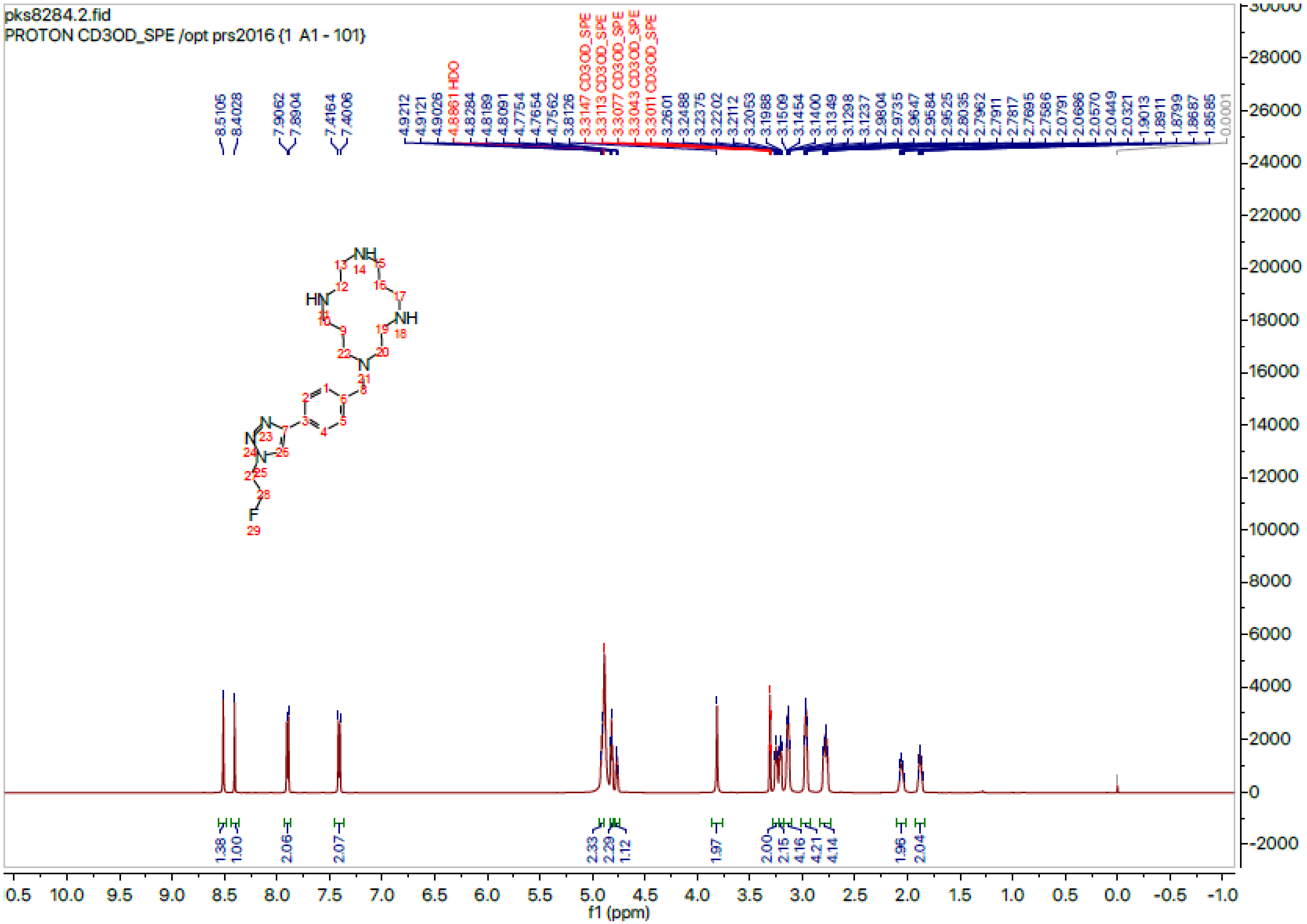
pks8284.2.fid
PROTON CD3OD_SPE /opt prs2016 {1 A1 - 101}
8.5105
8.4028
7.9062
7.8904
7.4164
7.4006
4.9212
4.9121
4.9026
4.8861 HDO
4.8284
4.8189
4.8091
4.7754
4.7654
4.7562
3.8126
3.3147 CD3OD_SPE
3.3113 CD3OD_SPE
3.3077 CD3OD_SPE
3.3043 CD3OD_SPE
3.3011 CD3OD_SPE
3.2601
3.2488
3.2375
3.2202
3.2112
3.2053
3.1988
3.1509
3.1454
3.1400
3.1349
3.1298
3.1237
2.9804
2.9735
2.9647
2.9584
2.9525
2.8035
2.7962
2.7911
2.7817
2.7695
2.7586
2.0791
2.0686
2.0570
2.0449
2.0321
1.9013
1.8911
1.8799
1.8687
1.8585
0.0001
1.38
1.00
2.06
2.07
2.33
2.29
1.12
1.97
2.00
2.15
4.16
4.21
4.14
1.96
2.04
f1 (ppm)

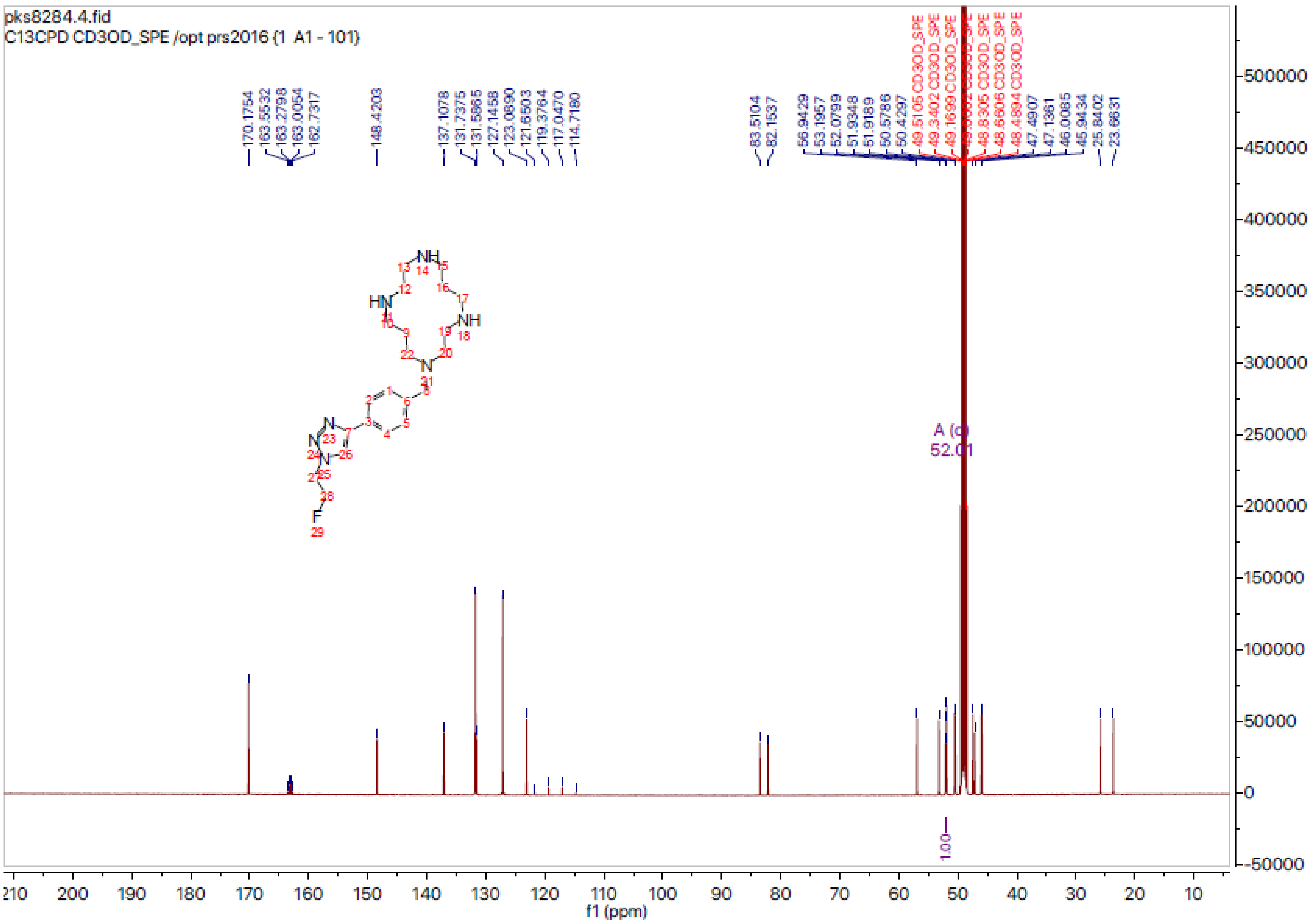
pks8284.4.fid
C13CPD CD3OD_SPE /opt prs2016 {1 A1 - 101}
f1 (ppm)

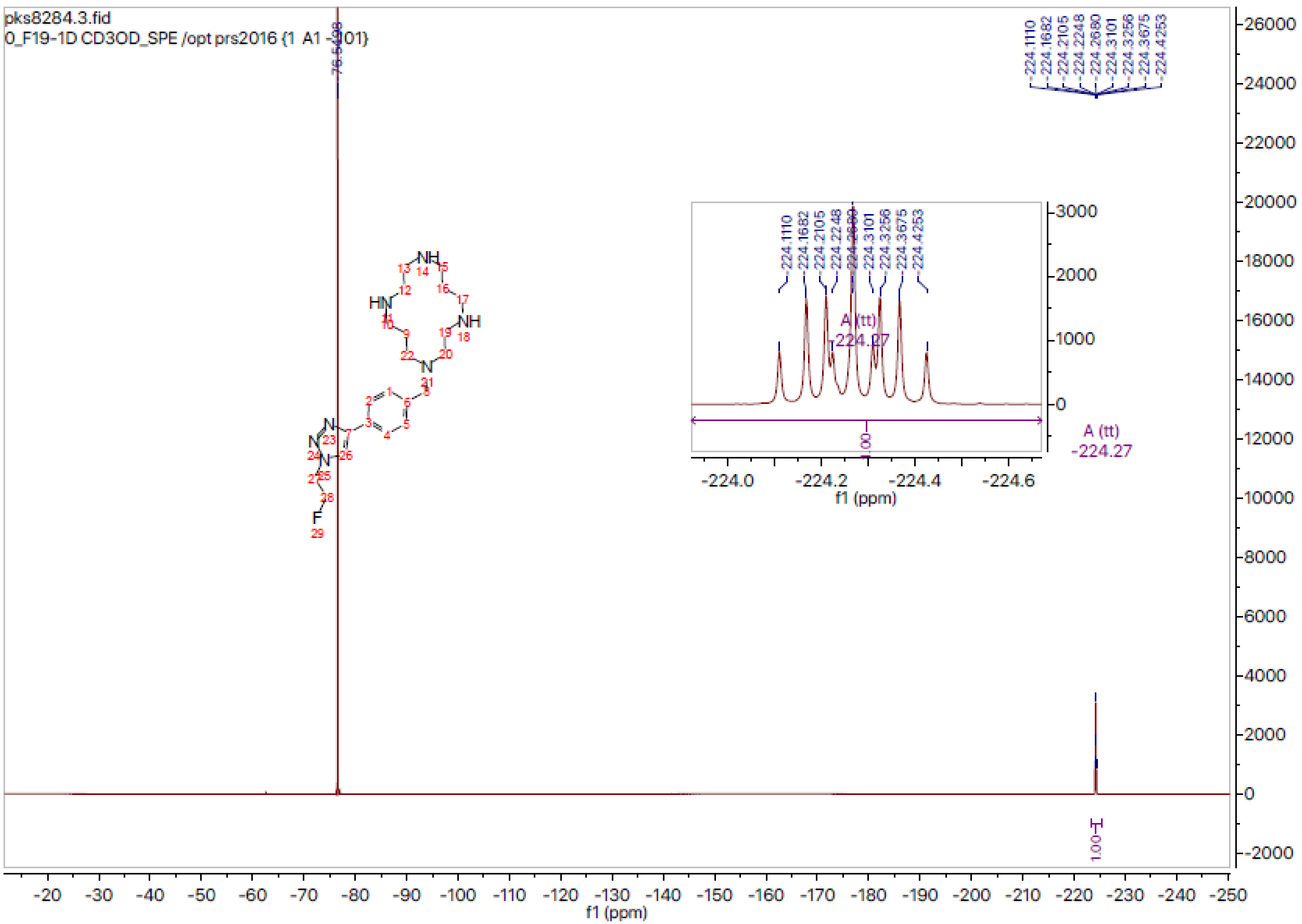

pks8284.3.fid
0_F19-1D CD3OD_SPE /opt prs2016 {1 A1 -301}
-76.5498
-224.1110
-224.1682
-224.2105
-224.2248
-224.2680
-224.3101
-224.3256
-224.3675
-224.4253
A (tt)
-224.27
1.00
f1 (ppm)

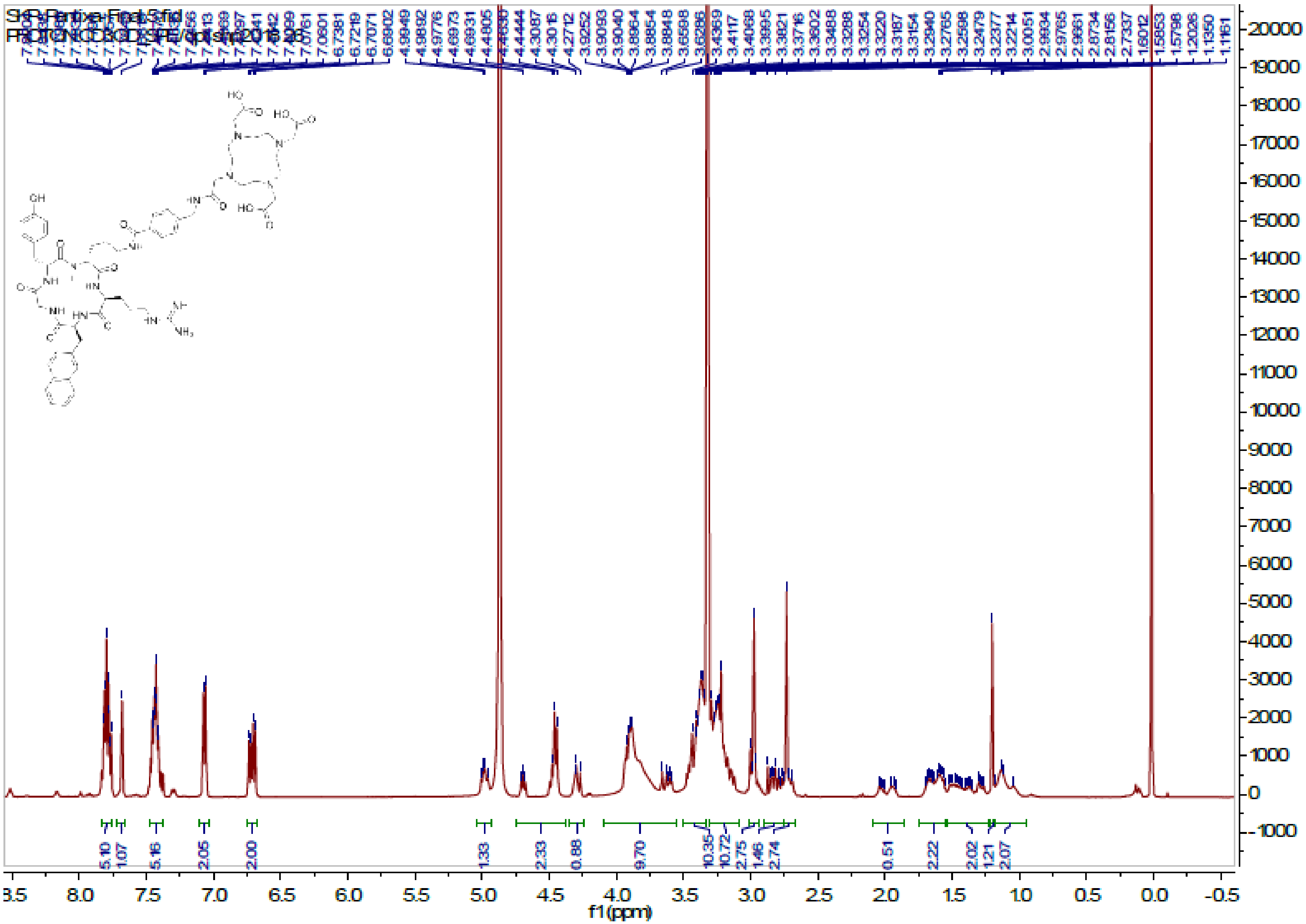

f1 (ppm)

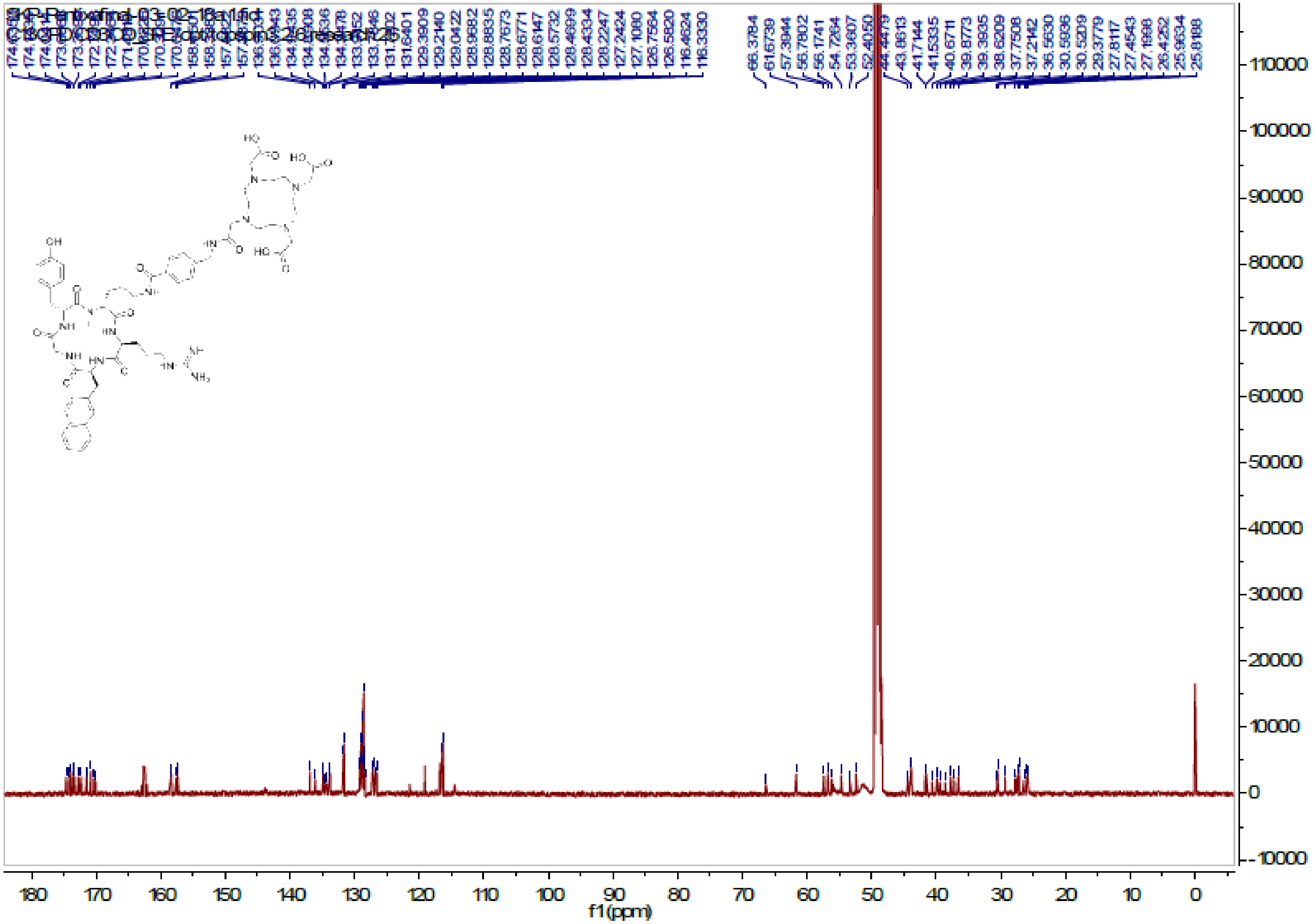

Supplement: Supplementary file 1 [file molecules-24-01612-s001.pdf]
